# Likelihood-free nested sampling for parameter inference of biochemical reaction networks

**DOI:** 10.1371/journal.pcbi.1008264

**Published:** 2020-10-09

**Authors:** Jan Mikelson, Mustafa Khammash

**Affiliations:** D-BSSE, ETH-Zurich, Zurich, Switzerland; University of Southern California, UNITED STATES

## Abstract

The development of mechanistic models of biological systems is a central part of Systems Biology. One major challenge in developing these models is the accurate inference of model parameters. In recent years, nested sampling methods have gained increased attention in the Systems Biology community due to the fact that they are parallelizable and provide error estimates with no additional computations. One drawback that severely limits the usability of these methods, however, is that they require the likelihood function to be available, and thus cannot be applied to systems with intractable likelihoods, such as stochastic models. Here we present a likelihood-free nested sampling method for parameter inference which overcomes these drawbacks. This method gives an unbiased estimator of the Bayesian evidence as well as samples from the posterior. We derive a lower bound on the estimators variance which we use to formulate a novel termination criterion for nested sampling. The presented method enables not only the reliable inference of the posterior of parameters for stochastic systems of a size and complexity that is challenging for traditional methods, but it also provides an estimate of the obtained variance. We illustrate our approach by applying it to several realistically sized models with simulated data as well as recently published biological data. We also compare our developed method with the two most popular other likeliood-free approaches: pMCMC and ABC-SMC. The C++ code of the proposed methods, together with test data, is available at the github web page https://github.com/Mijan/LFNS_paper.

## Author summary

The behaviour of mathematical models of biochemical reactions is governed by model parameters encoding for various reaction rates, molecule concentrations and other biochemical quantities. As the general purpose of these models is to reproduce and predict the true biological response to different stimuli, the inference of these parameters, given experimental observations, is a crucial part of Systems Biology. While plenty of methods have been published for the inference of model parameters, most of them require the availability of the likelihood function and thus cannot be applied to models that do not allow for the computation of the likelihood. Further, most established methods do not provide an estimate of the variance of the obtained estimator. In this paper, we present a novel inference method that accurately approximates the posterior distribution of parameters and does not require the evaluation of the likelihood function. Our method is based on the nested sampling algorithm and approximates the likelihood with a particle filter. We show that the resulting posterior estimates are unbiased and provide a way to estimate not just the posterior distribution, but also an error estimate of the final estimator. We illustrate our method on several stochastic models with simulated data as well as one model of transcription with real biological data.

This is a *PLOS Computational Biology* Methods paper.

## Introduction

The accurate modelling and simulation of biological processes such as gene expression or signalling has gained a lot of interest over the last years, resulting in a large body of literature addressing various types of models along with the means for their identification and simulation. The main purpose of these models is to qualitatively or quantitatively describe observed biological dynamics while giving insights into the underlying bio-molecular mechanisms.

One important aspect in the design of these models is the determination of the model parameters. Often there exists a mechanistic model of the cellular processes, but their parameters (e.g. reaction rates or initial molecule concentrations) are largely unknown. Since the same network topology may result in different behaviour depending on the chosen parameters ([1]), this presents a major challenge for modelling and underscores the need for effective parameter estimation techniques.

The models used in Systems Biology can be coarsely classified into two groups: deterministic and stochastic models. Deterministic models usually rely on ordinary differential equations which, given the parameters and initial conditions, can describe the time evolution of the biological system in a deterministic manner. However, many cellular processes like gene expression are subject to random fluctuations ([2, 3]), which can have important biological functions ([4–6]) as well as contain useful information about the underlying molecular mechanisms ([7]). The important role of stochastic fluctuations in biological systems has lead to increased interest in stochastic models and methods for their parameter inference ([8–12]). Such stochastic models are usually described in the framework of stochastic chemical reaction networks that can be simulated using Gillespie’s Stochastic Simulation Algorithm (SSA) [13]). In recent years, the availability of single-cell trajectory data has drastically increased, providing detailed information about the (potentially stochastic) development of single cells throughout time.

Despite the increasing interest in stochastic systems, performing inference on them is still challenging and the available methods are computationally very demanding (see for instance [8, 14, 15]). Several algorithms have been put forward to deal with such problems, such as various kinds of sequential Monte Carlo methods (SMC) ([16, 17]), Markov Chain Monte Carlo (MCMC) methods ([8, 18, 19]), approximate Bayesian computation (ABC) methods ([20, 21]), iterative filtering ([22]) and nested sampling (NS) approaches ([23–25]). However, for the problem of likelihood-free Bayesian inference the two most widely used methods are particle MCMC (pMCMC) ([26]) and ABC-SMC ([20]), as discussed in various recent review papers [27–29]. Furthermore, to reduce computational complexity, several of these inference methods rely on approximating the model dynamics (for instance using the diffusion approximation ([30]) or linear noise approximation ([31]). However, these approximations may not always be justifiable (in the case of low copy numbers of the reactants for example) and might obscure crucial system behaviour.

In this paper, we focus on nested sampling methods and investigate its applicability to stochastic systems. Coming originally from the cosmology community, NS has gained increasing popularity and found also applications in Systems Biology (see for instance [32–35]). Several implementations of NS are available ([36, 37]) and in [38] the authors even provide a NS implementation specifically for a Systems Biology context. Even though the original purpose of NS was to efficiently compute the Bayesian evidence, it has more and more become a viable alternative to MCMC methods for the approximation of the posterior (see for instance [39, 40]).

There are various reasons for the interest in NS which are discussed in detail in [41, 42] and the references within. Some of the rather appealing features of NS is that it performs well for multimodal distributions ([37, 39]), is easily parallelizable ([33, 43]) and provides a natural means to compute error bars on all of its results without needing multiple runs of the algorithm ([41, 44]). For a comparison of MCMC and NS see for instance [35, 41], for a discussion of other methods to compute the Bayesian evidence using MCMC see [41, 45]. Like standard MCMC methods, NS requires the availability of the likelihood which limits its use to models that allow for the computation of the likelihood such as deterministic models and simple stochastic models. In this paper, we consider an extension to the original NS framework that, similarly to the particle MCMC method ([46]) and particle SMC ([47]), allows the use of approximated likelihoods instead of the actual likelihood to be used for NS. In the following we introduce the notation and problem formulation, the “Materials and methods” section is dedicated to the likelihood-free NS formulation and in the section “Results” we demonstrate its performance on several chosen examples.

### Chemical reaction networks

We are considering a *n*_*x*_-dimensional Markov Process *X*(*t*) depending on a *d*-dimensional parameter vector *θ*. We denote with *X*_*i*_(*t*) the *i*^th^ entry of the state vector at time *t* and with $X\left(t\right)={\left\{X_{i}\left(t\right)\right\}}_{i=1,\ldots ,n_{x}}$ the state vector at time *t*. We will write *X*_*τ*_ = *X*(*t*_*τ*_) when talking about the state vector at a timepoint *t*_*τ*_ indexed with *τ*.

In the context of stochastic chemical reaction networks this Markov process describes the abundances of *n*_*x*_ species $X_{1},X_{2},\ldots ,X_{n_{x}}$, reacting through *n*_*R*_ reactions $R_{1},R_{2},\ldots ,R_{n_{R}}$ written as

$$\begin{array}{c} R_{j}:\;\sum_{i=1}^{n_{x}}p_{ji}X_{i}\to \sum_{i=1}^{n_{x}}q_{ji}X_{i}, \end{array}$$

where *p*_*ji*_ is the numbers of molecules of species $X_{i}$ involved in reaction $R_{j}$, and *q*_*ji*_ is the number of molecules of species $X_{i}$ produced by that reaction. The random variable *X*_*i*_(*t*) corresponds to the number of molecules of species $X_{i}$ at time *t*. Each reaction $R_{j}$ has an associated propensity. The reaction propensities at a given time *t* depend on the current state *X*(*t*) and on the parameter vector *θ*.

### General task

The process *X*(*t*) is usually not directly observable but can only be observed indirectly through a *n*_*y*_-dimensional observation vector

$$\begin{array}{c} Y_{\tau }∼p\left(\cdot |X_{\tau },\theta \right), \end{array}$$

which depends on the state *X*_*τ*_ and on the parameter vector *θ* ∈ Ω, where $\Omega \subseteq R^{d}$ denotes the parameter space. We use the notation *p*(⋅|*X*_*τ*_, *θ*) to emphasize that we think of the measurement *Y*_*τ*_ as a random variable sample from a conditional distribution *p*(⋅|*X*_*τ*_, *θ*). We shall assume that the variable *Y* is not observed at all times but only on *T* timepoints *t*_1_, …, *t*_*T*_ and only for *M* different trajectories. With ***y*** we denote the collection of observations at all time points. In the Bayesian approach the parameter vector *θ* is treated as a random variable with associated prior *π*(*θ*). The goal is not to find just one set of parameters, but rather to compute the posterior distribution P(θ|y) of *θ*

$$\begin{array}{c} P\left(\theta |y\right)=\frac{1}{Z}l\left(y|\theta \right)\pi \left(\theta \right), \end{array}$$

where *l*(***y***|*θ*) (we will also write *l*(*θ*) if the dependence on ***y*** is clear from the context) is the likelihood of *θ* for the particular observation ***y*** and *Z* is the Bayesian evidence

$$\begin{array}{c} Z=\int_{\Omega }l\left(y|\theta \right)d\pi \left(\theta \right). \end{array}$$ (1)

This has several advantages over a single point estimate as it gives insight into the areas of the parameter space resulting in model behaviour similar to the observations as well as about their relevance for the simulation outcome (a wide posterior indicates non-identifiability for example). The notation *dπ*(*θ*) above indicates that the integral is taken over the prior distribution. For a detailed discussion of Bayesian approaches see for instance [45]. In this paper we follow the Bayesian approach and aim to recover the posterior P(θ|y). In the following we briefly outline the basic nested sampling approach.

### Nested sampling (NS)

Nested sampling is a Bayesian inference technique that was originally introduced in [23] to compute the Bayesian evidence (1). NS can be viewed as an importance sampling technique (as for instance discussed in [48]) as it approximates the evidence by generating samples *θ*_*i*_, weights *w*_*i*_ and likelihoods *l*_*i*_ = *l*(*θ*_*i*_) such that the weighted samples can be used to obtain numerical approximations $\hat{Z}$ of the evidence (1)

$$\begin{array}{c} \hat{Z}=\sum_{i}w_{i}l_{i}\approx \int l\left(\theta \right)d\pi \left(\theta \right). \end{array}$$ (2)

NS samples parameter vectors (particles) from the prior distribution constrained to super-level sets of the likelihood

$$\begin{array}{c} \pi (\theta |l\left(\theta \right)>\epsilon_{i}) \end{array}$$ (3)

corresponding to an increasing sequence of thresholds *ϵ*_*i*_. The NS sampling scheme iteratively removes at each iteration *i* the particle *θ*_*i*_ with the lowest likelihood *l*(*θ*_*i*_) = *ϵ*_*i*_ from the current set of particles $L_{i}$ (called “live” set) and replaces it with a newly sampled particle with a likelihood higher than *ϵ*_*i*_ to obtain the next set $L_{i+1}$. This way, while still suffering from the “curse of dimensionality”, NS exponentially shrinks the sample space to regions of high likelihood and allows one to reach these regions in a reasonable number of iterations. Nested sampling exploits the fact that the Bayesian evidence (1) can also be written (see [23]) as a one-dimensional integral

$$\begin{array}{c} Z=\int_{0}^{1}L\left(x\right)dx, \end{array}$$

over the prior volume

$$\begin{array}{c} x\left(\epsilon \right)≔\pi \left(l\left(\theta \right)>\epsilon \right)=\int_{l(\theta )>\epsilon }d\pi \left(\theta \right), \end{array}$$

where *L*(*x*) denotes the likelihood corresponding to the constrained prior with volume *x*

$$\begin{array}{c} L\left(x\right)=\text{arg}\;\underset{\epsilon }{\text{inf}}\;\{x(\epsilon )\geq x\}. \end{array}$$ (4)

**Fig 1:**
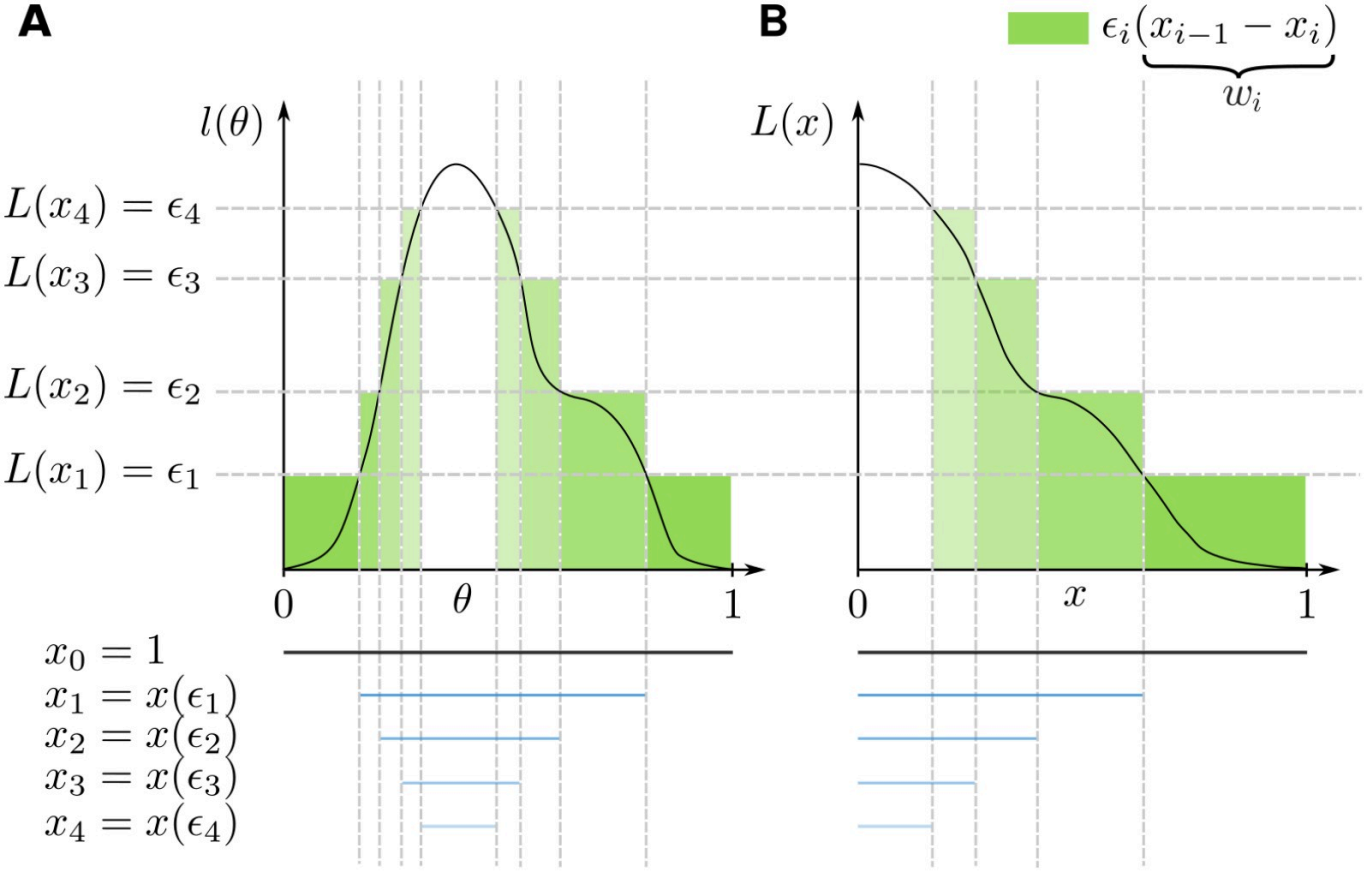
Illustration of the nested sampling approximation with a uniform prior on [0, 1]. **A**: The integral over the parameter space ∫_Ω_ *l*(*θ*)*dθ*. **B**: The transformed integral $\int_{0}^{1}L\left(x\right)dx$ over the prior volume *x*.

For this reformulation to hold some weak conditions have to be satisfied, see for details [49] and [25]. For an illustration of the above quantities see Fig 1A and 1B. The NS sampling scheme approximates the prior volumes *x*_*i*_ through ${\hat{x}}_{i}=t^{\left(i\right)}x_{i-1}$, where *t*^(*i*)^ denotes the *i*^th^ sample from a Beta distribution. These approximated prior volumes allow to compute the weights as $w_{i}={\hat{x}}_{i+1}-{\hat{x}}_{i}$ in (2). One can also use the weights *l*_*i*_ × *w*_*i*_ instead of *w*_*i*_ to approximate functions over the posterior P(θ)

$$\begin{array}{c} \frac{1}{\hat{Z}}\sum f\left(\theta_{i}\right)l_{i}w_{i}\approx \int f\left(\theta \right)dP\left(\theta \right). \end{array}$$

For a more detailed description of NS see S1 Appendix.

## Materials and methods

In many cases (such as most of the above mentioned stochastic models) the likelihood *l*(*θ*) cannot be directly computed, making approaches like MCMC methods or nested sampling not applicable. Fortunately, many variations of MCMC have been described circumventing this problem by introducing likelihood-free MCMC methods such as [50] or [46] as well as other likelihood-free methods such as ABC ([20]) or likelihood-free SMC methods ([51]). These approaches usually rely on forward simulation of a given parameter vector *θ* to obtain a simulated data set that can then be compared to the real data or can be used to compute a likelihood approximation $\hat{l}\left(\theta \right)\approx l\left(\theta \right)$. In the following we briefly illustrate one such likelihood approximation.

### Likelihood approximation using particle filters

A common way to approximate the likelihood through forward simulation is using a particle filter (see for instance [52]), which iteratively simulates the stochastic system with *H* particles and then resamples these particles. The main idea behind particle filters is to exploit the recursive relationship

$$\begin{array}{c} p\left(y_{1},\ldots ,y_{t}|\theta \right)=p\left(y_{1},\ldots ,y_{t-1}|\theta \right)\int p\left(y_{t}|X_{t}\right)p\left(X_{t}|y_{1},\ldots ,y_{t-1},\theta \right)dX_{t} \end{array}$$

where *y*_1_, …, *y*_*t*_ denotes all observations until timepoint *t* and *X*_*t*_ the (possibly unobserved) system state at time *t*. The above integral can be approximated by creating samples *x*_*t*,*i*_ from the distribution *p*(*X*_*t*_|*y*_1_, …, *y*_*t*−1_, *θ*), through forward simulation of of the system and resampling the simulated paths weighted by the likelihood at each timepoint. Then the likelihood for all observations up until timepoint *t* can be approximated by

$$\begin{array}{c} \int p\left(y_{t}|X_{t}\right)p\left(X_{t}|y_{1},\ldots ,y_{t-1},\theta \right)dX_{t}\approx \frac{1}{H}\sum_{h=1}^{H}p\left(y_{t}|x_{t,i}\right). \end{array}$$

**Fig 2:**
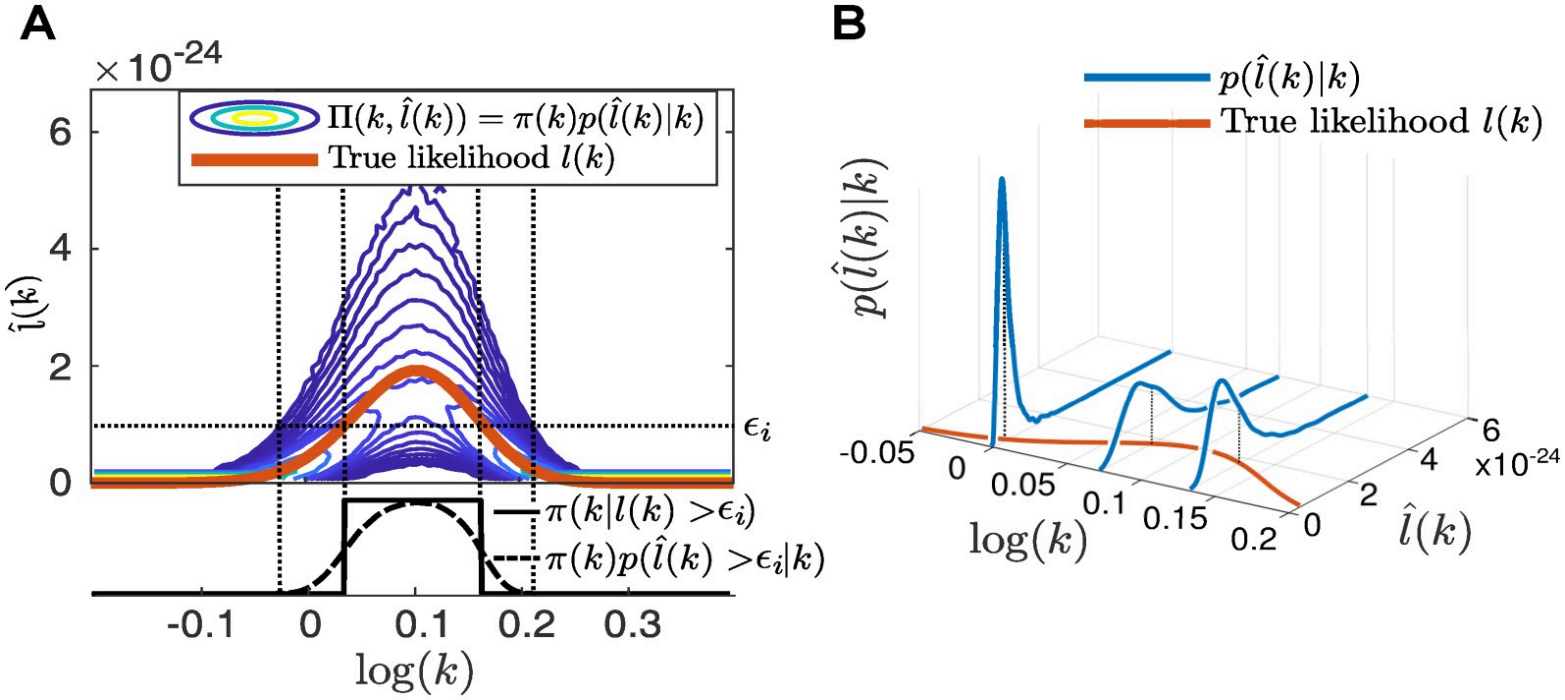
Illustration of likelihood approximation with a particle filter. **A**: Top: Likelihood for different parameters k (red) and contour lines of the joint distribution Π(*k*, log(*k*) of the parameter *k* and its likelihood approximation $\hat{l}\left(k\right)$, based on 10^6^ samples of the likelihood approximation obtained with a particle filter with 100 particles. Bottom: The constrained priors *π*(*k*|*l*(*k*) > *ϵ*) and $\pi \left(k\right)p(\hat{l}\left(k\right)>\epsilon |k)$ for *ϵ* = 1*e* − 24. **B**: Example distributions $p(\hat{l}\left(k\right)|k)$ (blue) for *k* = 1, 1.2 and 1.4 and the true likelihood *l*(*k*) red.

As the above approximation is unbiased, the resulting particle filter approximation of the likelihood is unbiased as well. In the following we illustrate such a particle filter likelihood approximation on a simple birth death model, where one species (mRNA) is produced at rate *k* = 1 and degrades at rate *γ* = 0.1. We simulated one trajectory of this system using SSA and, using the finite state projection (FSP [53]), computed the likelihood *l*(*k*) for different values of *k* while keeping *γ* fixed to 0.1. The true likelihood for different *k* is shown as the solid red line in Fig 2A and 2B. We also illustrated the likelihood approximation $\hat{l}\left(k\right)$ using a particle filter with *H* = 100 particles for three values of *k*. For each of the values for *k* we computed 1000 realizations of $\hat{l}\left(k\right)$ and plotted the empirical distributions in Fig 2B. Note that $\hat{l}\left(k\right)$ is itself a random variable with distribution $p(\hat{l}\left(k\right)|k)$ and has a mean equal to the true likelihood $E(\hat{l}\left(k\right))=l\left(k\right)$ (see for instance [52]). We also sampled 10^6^ values of *k* from a log uniform prior and approximate for each *k* its likelihood with the same particle filter with *H* = 100 particles. We plotted the contour lines of this joint distribution

$$\begin{array}{c} \Pi \left(k,\hat{l}\left(k\right)\right)=\pi \left(k\right)p\left(\hat{l}\left(k\right)|k\right) \end{array}$$

in Fig 2A.

In the following we discuss how to utilize such a likelihood approximation to apply the above described NS procedure to cases where the likelihood is not available. Throughout the paper we assume that the likelihood approximation $\hat{l}\left(\theta \right)$ is obtained using a particle filter, but our result hold for any unbiased likelihood estimator.

### The LF-NS scheme

For NS, the constraint prior *π*(*θ*|*l*(*θ*) > *ϵ*_*i*_) needs to be sampled. Since in the likelihood-free case, the likelihood *l*(*θ*) is not available and $\hat{l}\left(\theta \right)$ is itself a random variable, the set $\left\{\theta \in \Omega |\hat{l}\left(\theta \right)>\epsilon_{i}\right\}$ (which is the support of the constrained prior) is not defined. To apply the NS idea to the likelihood-free case, we propose to perform the NS procedure on the joint prior

$$\begin{array}{c} \Pi \left(\theta ,\hat{l}\left(\theta \right)\right)=\pi \left(\theta \right)p\left(\hat{l}\left(\theta \right)|\theta \right) \end{array}$$ (5)

on the set $\Omega \times R_{>0}$. This joint prior can be sampled by drawing a sample *θ*^⋆^ from the prior *π*(*θ*) and then drawing one sample ${\hat{l}}^{⋆}$ from the distribution of likelihood approximations $p(\hat{l}\left(\theta^{⋆}\right)|\theta^{⋆})$. With such a sampling scheme we perform the NS steps of constructing the set of “dead” particles D on the joint prior (5). As in standard NS, we sample a set of *N* “live” particles $\left\{\theta ,\hat{l}\right\}$ from $\Pi (\theta ,\hat{l}\left(\theta \right))$, then we iteratively remove the particle $\left\{\theta ,\hat{l}\right\}$ with the lowest likelihood sample $\hat{l}$ from the set of live points and add it to the dead points. The LF-NS algorithm is shown in Algorithm 1.

**Algorithm 1**: Likelihood-free nested sampling algorithm

1: Given observations ***y***, a prior *π*(*θ*) for *θ* and a likelihood approximation $p(\hat{l}\left(\theta \right)|\theta )$.

2: Sample *N* particles $\left\{\theta^{k},{\hat{l}}^{k}\right\}$ from the prior $\Pi (\theta ,\hat{l}\left(\theta \right))$ and save it in the set $L_{0}$, set D={∅}

3: **for** i = 1, 2, …, m **do**

4:  Find $i^{\prime }=\text{arg}\;\underset{k}{\text{min}}({\hat{l}}^{k}|\left\{\theta^{k},{\hat{l}}^{k}\right\}\in L_{i-1})$ and set $\theta_{i}=\theta^{i^{\prime }}$ and $\epsilon_{i}={\hat{l}}^{i^{\prime }}$

5:  Add {*θ*_*i*_, *ϵ*_*i*_} to D

6:  Set $L_{i}=L_{i-1}\backslash \left\{\theta_{i},\epsilon_{i}\right\}$

7:  Sample $\left\{\theta^{⋆},{\hat{l}}^{⋆}\right\}∼\Pi \left(\theta ,\hat{l}\left(\theta \right)|\hat{l}\left(\theta \right)>\epsilon_{i}\right)$ and add it to $L_{i}$

8: **end for**

The parallel version of LF-NS with *r* parallel processes is analogous to the parallelization of the standard NS algorithm as described in S2 Appendix.

### LF-NS is unbiased

As for standard NS, the sampling procedure for LF-NS guarantees that each set of live points $L_{i}$ contains *N* samples uniformly distributed according to the constrained joint prior $\Pi (\theta ,\hat{l}\left(\theta \right)|\hat{l}\left(\theta \right)>\epsilon_{i})$, thus removing the sample with the lowest likelihood approximation ${\hat{l}}^{k}$ results in the same shrinkage of prior volume as the standard NS scheme. The prior volumes *x*_*i*_ = *t*^(*i*)^ *x*_*i*−1_ now correspond to the volumes of the constraint joint priors $\Pi (\theta ,\hat{l}\left(\theta \right)|\hat{l}\left(\theta \right)>\epsilon_{i})$ and the resulting weights $w_{i}={\hat{x}}_{i-1}-{\hat{x}}_{i}$ can be used, similarly as in Eq (2), to integrate the likelihood *l* over the constrained prior. Writing the density functions of *π*(*θ*) and $p(\hat{l}\left(\theta \right)|\theta )$ as *f*_*π*_(*θ*) and $f_{p}\left(\hat{l}\left(\theta \right)|\theta \right)$ respectively we have

$$\begin{array}{c} \begin{array}{l} \sum_{i}^{m}w_{i}\epsilon_{i}\approx \int \hat{l}(\theta )d\Pi (\theta ,\hat{l}(\theta ))\\ =\underset{\Omega }{\int }\overset{\infty }{\underset{0}{\int }}\hat{l}(\theta )f_{\pi }(\theta )f_{p}(\hat{l}(\theta )|\theta )d\hat{l}(\theta )d\theta =\underset{\Omega }{\int }f_{\pi }(\theta )\overset{\infty }{\underset{0}{\int }}\hat{l}(\theta )f_{p}(\hat{l}(\theta )|\theta )d\hat{l}(\theta )d\theta \\ =\underset{\Omega }{\int }l(\theta )f_{\pi }(\theta )d\theta =Z, \end{array} \end{array}$$

where the last equality relies on the unbiasedness of $\hat{l}\left(\theta \right)$. While the procedure for LF-NS is very similar to the standard NS algorithm, the new samples *θ*^⋆^ have to be drawn from the constraint joint prior $\Pi (\theta ,\hat{l}\left(\theta \right)|\hat{l}\left(\theta \right)>\epsilon )$ instead from the constrained prior *π*(*θ*|*l*(*θ*) > *ϵ*). In the following we discuss the resulting difficulties and show how to overcome them.

### Sampling from the super-level sets of the likelihood

One of the main challenges ([54, 55]) in the classical NS algorithm is the sampling from the prior constrained to higher likelihood regions *π*(*θ*|*l*(*θ*) > *ϵ*). A lot of effort has been dedicated to finding ways to sample from the constrained prior efficiently, the most popular approaches include slice sampling ([37]) and ellipsoid based sampling ([39]).

In the case of LF-NS, at the *i*^th^ iteration we are sampling not just a new parameter vector *θ*^⋆^ but also a realization of its likelihood approximation ${\hat{l}}^{⋆}$ from

$$\begin{array}{c} \Pi \left(\theta ,\hat{l}\left(\theta \right)|\hat{l}\left(\theta \right)>\epsilon_{i}\right)=\pi \left(\theta \right)p\left(\hat{l}\left(\theta \right)|\theta ,\hat{l}\left(\theta \right)>\epsilon_{i}\right). \end{array}$$ (6)

Since it is in general not possible to sample ${\hat{l}}^{⋆}$ from the constraint distribution $p(\hat{l}\left(\theta {}^{⋆}\right)|\theta {}^{⋆},\hat{l}\left(\theta {}^{⋆}\right)>\epsilon_{i})$ directly, we use rejection sampling. We sample *θ*^⋆^ from the prior *π*(*θ*), then sample ${\hat{l}}^{⋆}$ from the unconstrained distribution $p(\hat{l}\left(\theta^{⋆}\right)|\theta^{⋆})$ and accept the pair $\left(\theta^{⋆},{\hat{l}}^{⋆}\right)$ only if ${\hat{l}}^{⋆}>\epsilon_{i}$. While this procedure guarantees that the resulting samples are drawn from (6), the acceptance rate might become very low. Each live set $L_{i}$ consists of *N* pairs $\left(\theta^{k},{\hat{l}}^{k}\right)$ distributed according to (6), thus the parameter vectors *θ*^*k*^ in $L_{i}$ are distributed according to

$$\begin{array}{c} \theta ∼\int \Pi \left(\theta ,\hat{l}\left(\theta \right)|\hat{l}\left(\theta \right)>\epsilon_{i}\right)d\hat{l}\left(\theta \right)=\pi \left(\theta \right)p\left(\hat{l}\left(\theta \right)>\epsilon_{i}\right). \end{array}$$ (7)

We plotted an example of the distributions (3) and (7) for the example of the birth-death process in Fig 2A. The distribution (7) has usually an infinite support, although in practice the density function of (7) will be close to zero for large areas of the parameter space Ω. Similarly to NS, we propose to use the set $L_{i}$ to drawn from the areas where (7) is non-zero. Slice sampling methods ([32, 37]) are unfortunately not applicable for LF-NS since they require a way to evaluate its target distribution at each of its samples. We can still use ellipsoid sampling schemes, but unlike in the case of NS where the target distribution *π*(*θ*|*l*(*θ*) > *ϵ*) has compact support, the target distribution (7) has potentially infinite support framing ellipsoid based sampling approaches rather unfitting. Sampling using MCMC methods (as suggsted in [23]) is expected to work even for target distributions with infinite support, but suffers from the known MCMC drawbacks, as they produce correlated samples and might get stuck in disconnected regions.

To account for the smooth shape of (7) we propose to employ a density estimation approach. At each iteration *i*, we estimate the density $\pi \left(\theta \right)p(\hat{l}\left(\theta \right)>\epsilon_{i})$ from the live points and employ a rejection sampling approach to sample uniformly from the prior on the domain of this approximation. As density estimation technique, we use Dirichlet Process Gaussian Mixture Model (DP-GMM) ([56]). For further details and an illustration of the different sampling schemes see S3 Appendix.

Even though for the presented examples we employ DP-GMM, we note that in theory any sampling scheme that samples uniformly from the prior *π*(*θ*) over the support of $\pi \left(\theta \right)p(\hat{l}\left(\theta \right)>\epsilon_{i})$ will work.

### A lower bound on the estimator variance

Unlike for NS, for LF-NS, even if at each iteration the proposal particle *θ*^⋆^ is sampled from the support of $\pi \left(\theta \right)p(\hat{l}\left(\theta \right)>\epsilon_{i})$, it will only be accepted with probability $p(\hat{l}\left(\theta^{⋆}\right)>\epsilon_{i})$. This means that depending on the variance of the likelihood estimation $p(\hat{l}\left(\theta \right)|\theta )$ and the current likelihood threshold *ϵ*_*i*_, the acceptance rate for LF-NS will change and with it the computational cost. For an illustration see S4 Appendix.

Due to this possible increase in computational time, it is important to terminate the LF-NS algorithm as soon as possible. We propose to use for the Bayesian evidence estimation not only the dead particles D, but also the current live points $L_{m}$. This possibility has been already mentioned in other places (for instance in [40, 49, 57]) but is usually not applied, since the contribution of the live particles decreases exponentially with the number of iterations. Since for standard NS each iteration is expected to take the same amount of time, most approaches simply increase the number of iterations to make the contribution of the live particles negligibly small.

The Bayesian evidence can be decomposed as

$$\begin{array}{c} Z=\underset{≕Z_{L}^{m}}{\underset{︸}{\int_{0}^{x_{m}}L\left(x\right)dx}}+\underset{≕Z_{D}^{m}}{\underset{︸}{\int_{x_{m}}^{1}L\left(x\right)dx}} \end{array}$$ (8)

where *x*_*m*_ is the prior volume for iteration *m*. The first integral $Z_{L}^{m}$ is the part that can be approximated through the *N* live samples at any given iteration, while the integral $Z_{D}^{m}$ is approximated through the dead samples.

Writing

$$\begin{array}{c} {\bar{L}}_{m}≔\frac{1}{N}\sum_{\left\{\theta ,\hat{l}\right\}\in L_{m}}\hat{l}\approx \underset{≕L_{m}}{\underset{︸}{\int \hat{l}\left(\theta \right)d\Pi \left(\theta ,\hat{l}\left(\theta \right)|\hat{l}\left(\theta \right)>\epsilon_{m}\right)}} \end{array}$$

for the estimator of the integral of the likelihoods in the live set, we propose the following estimator for *Z*

$$\begin{array}{c} {\hat{Z}}_{\text{tot}}^{m}=\underset{={\hat{Z}}_{L}^{m}\approx Z_{L}^{m}}{\underset{︸}{{\hat{x}}_{m}{\bar{L}}_{m}}}+\underset{={\hat{Z}}_{D}^{m}\approx Z_{D}^{m}}{\underset{︸}{\sum_{i=1}^{m}\epsilon_{i}w_{i}}}, \end{array}$$ (9)

where ${\hat{Z}}_{D}^{m}$ approximates the finite sum ${\tilde{Z}}_{D}^{m}=\sum_{i=1}^{m}\epsilon_{i}\left(x_{i-1}-x_{i}\right)$ by replacing the random variables *x*_*i*_ with their means ${\hat{x}}_{i}$. Since ${\hat{x}}_{m}{\bar{L}}_{m}$ is an unbiased estimator of $Z_{L}^{m}$ and ${\hat{Z}}_{D}^{m}$ is an unbiased estimator of $Z_{D}^{m}$, the estimator ${\hat{Z}}_{\text{tot}}^{m}$ is an unbiased estimator of the Bayesian evidence *Z* for any *m*. In particular, this implies that terminating the LF-NS algorithm at any iteration *m* will result in an unbiased estimate for *Z*. However, terminating the LF-NS algorithm early on will still result in a very high variance of the estimator. This variance is a result of the variances in *x*_*i*_ and the variance in the Monte Carlo estimate ${\bar{L}}_{m}$. As pointed out in [40], when using nested sampling approximations to approximate the integral of arbitrary functions *f* over the posterior, an additional error is introduced by approximating the average value of *f*(*θ*) on the contour line of *l*(*θ*) = *ϵ*_*i*_ with the value *f*(*θ*_*i*_). In the following we formulate a lower bound $\sigma_{\text{min}}^{2m}$ on the estimator variance $\sigma_{\text{tot}}^{2m}=\text{Var}({\tilde{Z}}_{D}^{m}+{\hat{Z}}_{L}^{m})$ at iteration *m*, show that this lower bound is monotonically increasing in *m* and propose to terminate the LF-NS algorithm as soon as the current estimator variance differs from this lower bound by no more than a predefined threshold *δ*.

Treating the prior volumes *x*_*i*_ and the Monte Carlo estimate ${\bar{L}}_{m}$ as random variables, the variance $\sigma_{\text{tot}}^{2m}$ of the NS estimator at iteration *m* can be estimated at each iteration without additional computational effort (see S5 Appendix and [57]). This variance depends on the variance $\mathrm{Var}({\bar{L}}_{m})$ of the Monte Carlo estimate ${\bar{L}}_{m}$ and is monotonically increasing in $\mathrm{Var}({\bar{L}}_{m})$ (see S5 Appendix). We define the term $\sigma_{\text{min}}^{2m}$ which is the same variance $\mathrm{Var}({\tilde{Z}}_{D}^{m}+{\hat{Z}}_{L}^{m})$ but with the term $\text{Var}({\bar{L}}_{m})$ set to 0. Clearly we have for any *m* (see S6 Appendix)

$$\begin{array}{c} \sigma_{\text{tot}}^{2m}\geq \sigma_{\text{min}}^{2m}. \end{array}$$

More importantly, as we show in S6.2 Appendix, $\sigma_{\text{min}}^{2}$ is monotonically increasing in *m*

$$\begin{array}{c} \sigma_{\text{min}}^{2m^{\prime }}\geq \sigma_{\text{min}}^{2m},\;\forall m^{\prime }\geq m. \end{array}$$

This allows us to bound the lowest achievable estimator variance $\sigma_{\text{min}}^{2}=\underset{m\to \infty }{\text{sup}}\sigma_{\text{min}}^{2m}$ from below

$$\begin{array}{c} \sigma_{\text{min}}^{2}\geq \sigma_{\text{min}}^{2m}. \end{array}$$

The terms for $\sigma_{\text{tot}}^{2m}$ and $\sigma_{\text{min}}^{2m}$ both contain the unknown value *L*_*m*_ which can be approximated using its Monte Carlo estimate ${\bar{L}}_{m}$ giving us the estimations of the above variances ${\hat{\sigma }}_{\text{tot}}^{2m}$ and ${\hat{\sigma }}_{\text{min}}^{2m}$. We use these variance estimates to formulate a termination criteria by defining

$$\begin{array}{c} \Delta_{LFNS}^{m}≔\frac{\sqrt{{\hat{\sigma }}_{\text{tot}}^{2m}}-\sqrt{{\hat{\sigma }}_{\text{min}}^{2m}}}{{\hat{Z}}_{\text{tot}}^{m}} \end{array}$$ (10)

and terminate the algorithm as soon as $\Delta_{LFNS}^{m}<\delta$ for some predefined *δ*. The term in the numerator in (10) is the difference between the current estimator standard deviation ${\hat{\sigma }}_{\text{tot}}^{m}$ and the lowest standard deviation ${\hat{\sigma }}_{\text{min}}^{m}$ that can possibly be achieved by continuing to run the LF-NS algorithm beyond the current iteration *m*. The denominator is used to normalized this possible reduction in standard deviation by the current evidence estimate ${\hat{Z}}_{\text{tot}}^{m}$. This termination criteria seems intuitive since it terminates the LF-NS algorithm as soon as a continuation of the algorithm is not expected to make the final estimator significantly more accurate. As a final remark we note that the final estimator ${\hat{Z}}_{\text{tot}}^{m}$ as well as the termination criteria using $\Delta_{LFNS}^{m}$ can of course also be applied in the standard NS case.

## Results

We test our proposed LF-NS algorithm on three examples for stochastic reaction kinetic models. The first example is the birth death model, already introduced above, the second example is the Lac-Gfp model used for benchmarking in [10] and the third example is a transcriptional model from [58] with corresponding real data. We also compare the performance of the LF-NS algorithm with two of the most widely used likelihood-free inference methods pMCMC and ABC-SMC. We point out that for deterministic systems with available likelihoods, our LF-NS algorithm reduces to the standard NS method and has been discussed in several other places (see for instance [41]). In the following examples all priors are chosen as uniform or log-uniform in the bounds indicated in the posterior plots.

### The stochastic birth-death model

**Fig 3:**
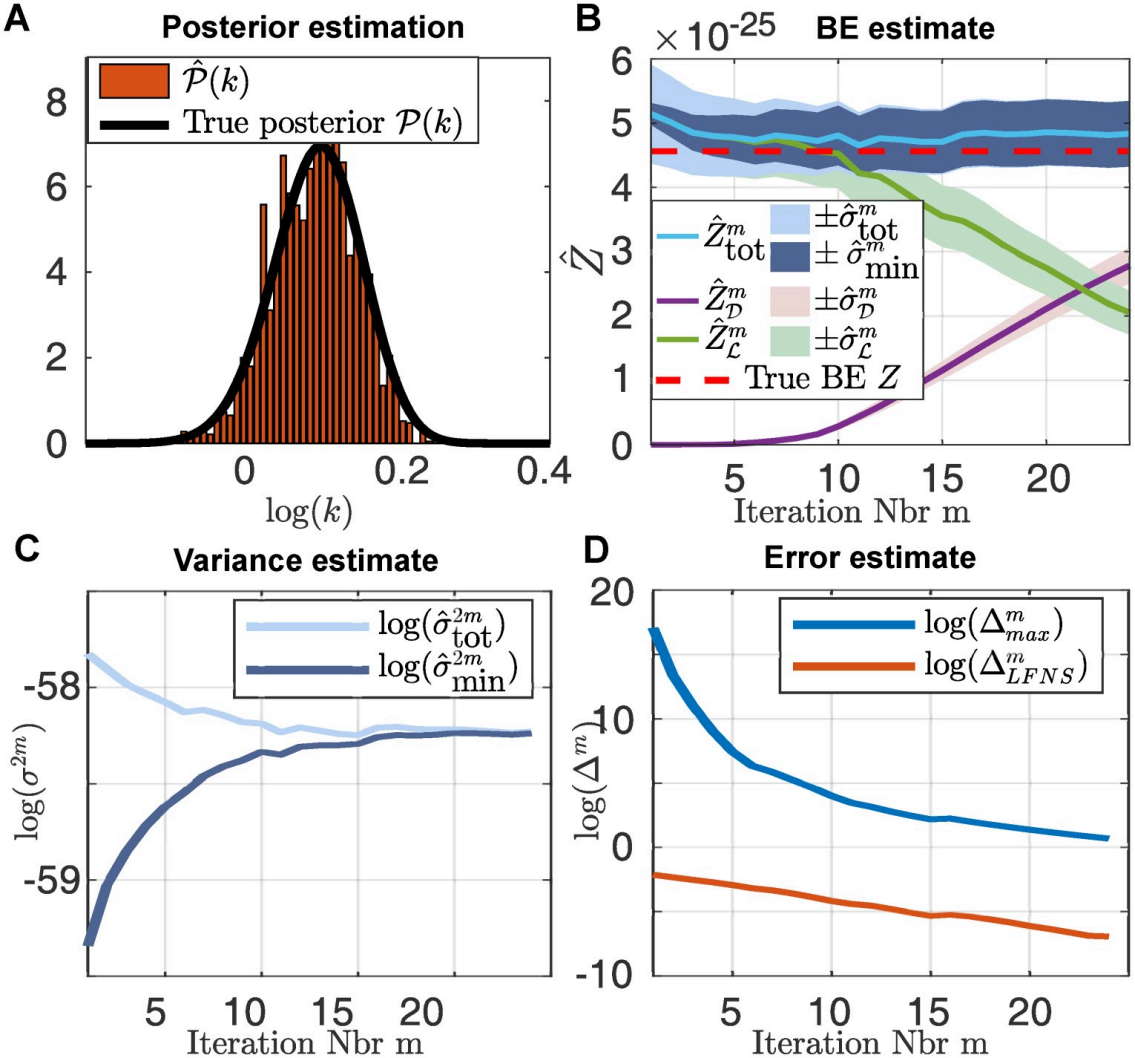
Inference on the birth-death model. **A**: Histogram of the posterior P(k) estimate obtained with LF-NS using *N* = 100 and *H* = 100. The true posterior is indicated in black. **B**: Development of the estimation of the Bayesian evidence using the estimation based solely on the dead points ${\hat{Z}}_{D}$, the estimate approximation from the live points ${\hat{Z}}_{L}$ and the estimation based on both ${\hat{Z}}_{\text{tot}}$. The corresponding standard errors are indicated as the shaded areas. The true Bayesian evidence is indicated with the dashed red line. **C**: Estimate of the current variance estimate ${\hat{\sigma }}_{\text{tot}}^{2m}$ and the lower bounds for the lowest achievable variance ${\hat{\sigma }}_{\text{min}}^{2}$. **D**: Developments of the different error estimations for each iteration.

We first revisit the birth-death example from above to compare our inference results to the solution obtained by FSP. We use the same data as above and use the same log-uniform prior. We ran our LF-NS algorithm as described above using DP-GMM for the sampling. We used *N* = 100 LF-NS particles, *H* = 100 particle filter particles and sample at each iteration *r* = 10 particles. We ran the LF-NS algorithm until $\Delta_{LFNS}^{m}$ is smaller than 0.001. We show the obtained posterior in Fig 3A. Fig 3B shows the obtained estimates of the Bayesian evidence, where the shaded areas indicate the standard error at each iteration. The dashed red line indicates the true BE computed from 10^6^ samples from $\Pi (\theta ,\hat{l}\left(\theta \right))$. We observe that due to the simplicity of the birth-death model, the BE is already well estimated in the very first iteration. However, the estimates of the lower and upper bound of the variance ${\hat{\sigma }}_{\text{min}}^{2m}$ and ${\hat{\sigma }}_{\text{tot}}^{2m}$ indicate that a continuation of LF-NS scheme may result in a lower estimator variance. The development of these upper and lower bound estimators are shown in Fig 3C and we can clearly see how they converge to the same value. For our termination criteria we show the quantities $\Delta_{\text{max}}^{m}=x_{m}\text{max}\left(\hat{l}\in L_{m}\right)$, that is frequently used as a termination criteria for regular nested sampling (see S1.3 Appendix) and $\Delta_{LFNS}^{m}$ in Fig 3D.

### The Lac-Gfp model

We demonstrate how our algorithm deals with a realistic sized stochastic model, by inferring the posterior for the parameters of the Lac-Gfp model illustrated in S5 Fig. This model has been already used in [10] as a benchmark, although with distribution-data. Here we use the model to simulate a number of trajectories and illustrate how our approach infers the posterior of the used parameters. This model is particularly challenging in two aspects. First, the number of parameters is 18, making it a fairly large model to infer. Secondly, the model exhibits switch-like behaviour which makes it very hard to approximate the likelihood of such a switching trajectory (see S7.2 Appendix and particular S6 Fig for further details). We used *N* = 500 LF-NS particles, *H* = 500 particle filter particles and sample at each iteration *r* = 50 particles.

**Fig 4:**
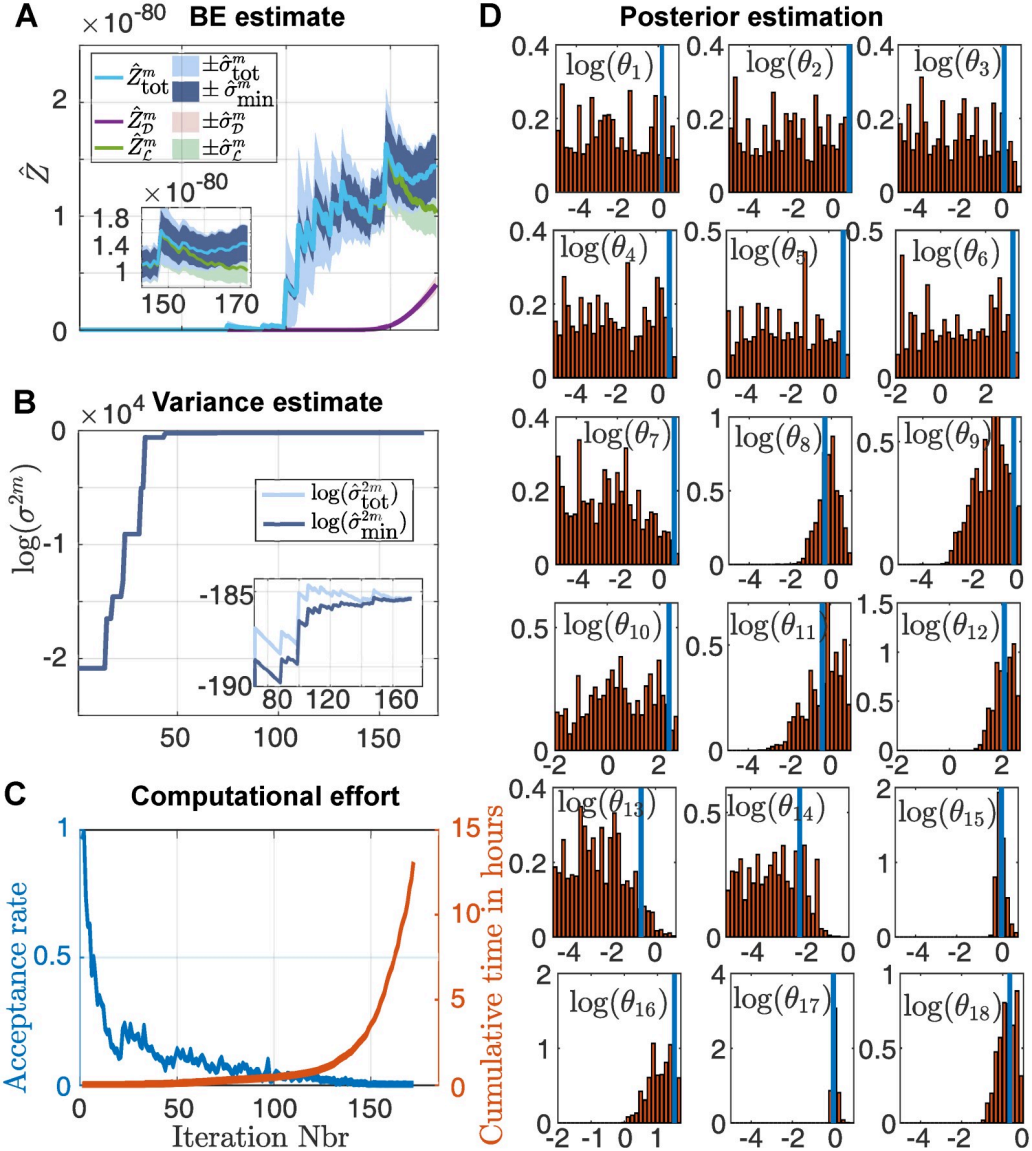
Inference on the Lac-Gfp model. **A**: Development of the estimation of the Bayesian evidence using the estimation based solely on the dead points ${\hat{Z}}_{D}$, the estimate approximation from the live points ${\hat{Z}}_{L}$ and the estimation that uses both ${\hat{Z}}_{\text{tot}}$. The corresponding standard errors are indicated as the shaded areas. **B**: Estimate of the current variance estimate ${\hat{\sigma }}_{\text{tot}}^{2m}$ and the lower bounds for the lowest achievable variance ${\hat{\sigma }}_{\text{min}}^{2}$. **C**: The acceptance rate of the LF-NS algorithm for each iteration (blue) and the cumulative time needed for each iteration in hours (red). The computation was performed on 48 cores in parallel on the Euler cluster of the ETH Zurich. **D**: Marginals of the inferred posterior distributions of the parameters based on one simulated trajectory. The blue lines indicate the parameters used for the simulation of the data.

The measured species in this example is fluorescent Gfp (mGFP) where it is assumed that each Gfp-molecule emits fluorescence according to a normal distribution. We used one trajectory to infer the posterior, whose marginals are shown in Fig 4D. The posterior covers the true parameters (indicated in blue) and we can observe that while several parameters (particularly *θ*_1_—*θ*_7_) cannot be well identified by the posterior, the remaining parameters seem to have been identified well. Fig 4A shows the estimated Bayesian evidence with corresponding standard errors for each iteration. Fig 4B shows the corresponding estimations of the bounds of the lowest achievable variance. As we see, the estimated Bayesian evidence, as well as the estimated variance bounds, do several jumps in the process of the LF-NS run. These jumps are due to the exploration of the parameter space and correspond to iterations in which previously unsampled areas of the parameter space got sampled with a new maximal likelihood. In Fig 4C we plotted the acceptance rate of the LF-NS algorithm for each iteration as well as the cumulative computational time. The computation was performed on 48 cores of the Euler cluster of the ETH Zurich. The inference for this model took well over 12 hours and as we see, the computational time for each iteration seems to increases exponentially as the acceptance rate decreases. The low acceptance rate is expected, since the number of particle filter particles *H* = 500 results in a very high variance of the particle filter estimate (see S6B Fig). From Fig 4A we can clearly see that using only the dead points for the BE estimation would result in a very strong underestimation of the true BE. The use of the live sets and our developed termination criteria is what allows us to terminate the algorithm in a reasonable time. We can also see from Fig 4A that our LF-NS algorithm seems to approximated the BE already quite well after around 110 iterations. To reach this iteration the LF-NS needed only approximately 35 minutes (as can be seen in Fig 4C), and we conclude that the majority of the computational time is used not to find new regions of high likelihood, but rather to reduce the variance of the final BE estimator. We point out that we could have terminated the algorithm after the first hour and would have had already a good estimate of the BE, where the final variance (as seen in Fig 4B) would still be in an acceptable range. The runtime of around 13 hours is therefore mainly due to our very high standards of accuracy.

### A stochastic transcription model

**Fig 5:**
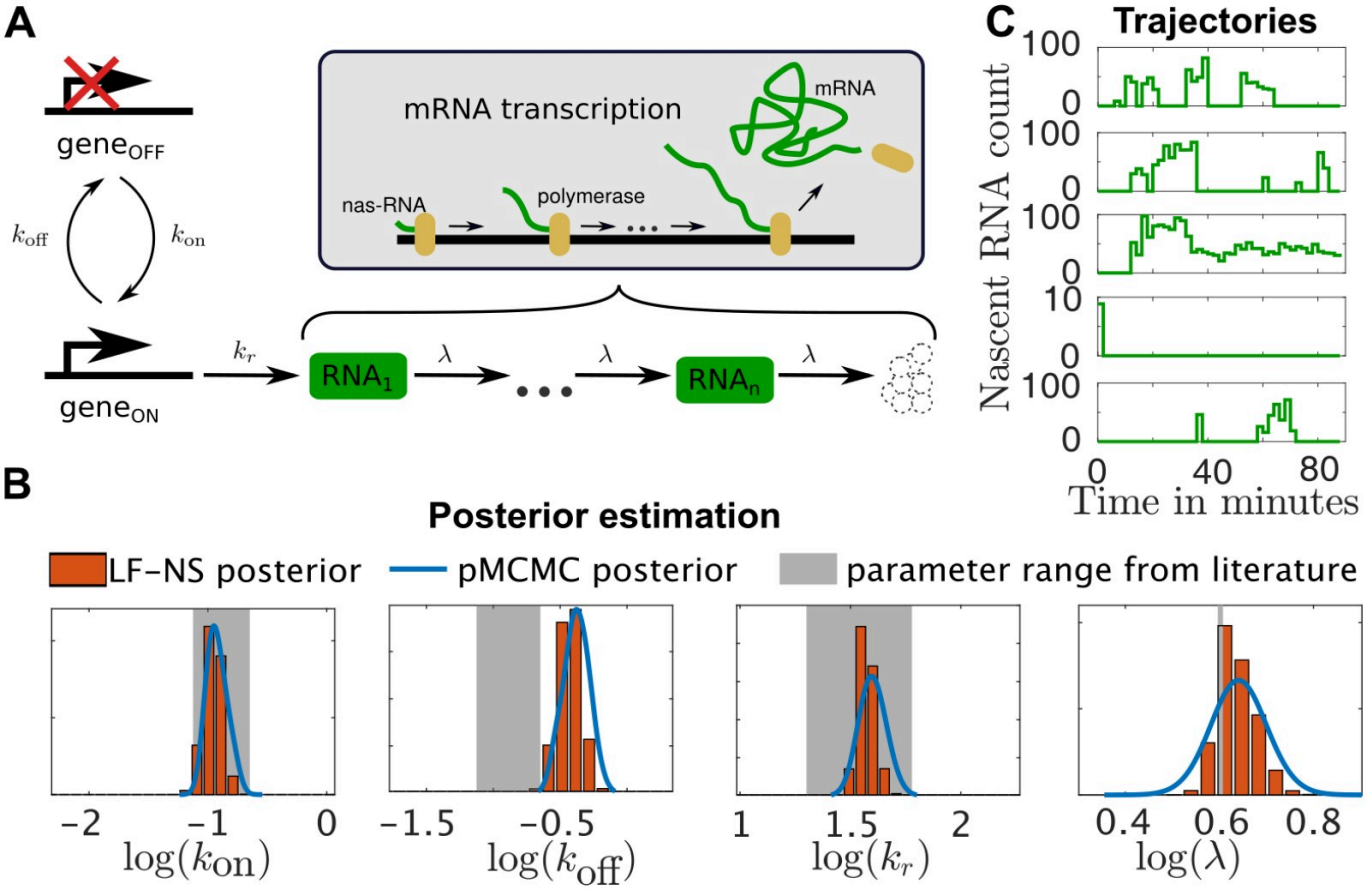
Inference on the transcription model. **A**: Schematic representation of the gene expression model. The model consists of a gene that switches between an “on” and an “off” state with rates *k*_on_ and *k*_off_. When “on” the gene is getting transcribed at rate *k*_*r*_. The transcription process is modelled through *n* RNA species that sequentially transform from one to the next at rate λ. The observed species are all of the intermediate *RNA*_*i*_ species. **B**: The marginal posterior distribution of the parameters of the system. The histogram indicates the posterior obtained through LF-NS, the blue line indicates the posterior obtained from a long pMCMC run and the shaded areas indicate the parameter ranges that were considered in [58]. The scale of the x-axis does not represent the range of the prior and has been chosen for presentational purposes. **C**: The five trajectories used for the parameter inference.

As a third example we use a transcription model recently used in [58], where an optogenetically inducible transcription system is used to obtain live readouts of nascent RNA counts. The model consists of a gene that can take two configurations “on” and “off”. In the “on” configuration mRNA is transcribed from this gene and can be individually measured during this transcription process (see [58] for details). We modelled the transcription through *n* = 8 subsequent RNA species that change from one to the next at a rate λ. This is done to account for the observed time of 2 minutes that one transcription event takes. An illustration of the model is shown in Fig 5A. For the inference of the model parameters we chose five trajectories of real biological data, shown in Fig 5C. Clearly, the system is inherently stochastic and requires corresponding inference methods. We ran the LF-NS algorithm for *N* = 500 and *H* = 500 on these five example trajectories. The resulting marginal posteriors are shown in Fig 5B, we also indicated the parameter ranges considered in [58]. These ranges were chosen in [58] in an ad-hoc manner but, apart from the values for *k*_off_, seem to fit very well with our inferred results. To make sure that our approach inferred the right posterior, we also ran a pMCMC algorithm (see S8.1 Appendix) on the problem. pMCMC methods have been shown to target the true posterior distribution, which is why we chose the obtained pMCMC posterior as ground truth. To make sure that the pMCMC run converged in an acceptable time, we used the posterior obtained from the LF-NS run to pick the initial sample of pMCMC. We also picked the perturbation kernel *q* for pMCMC to be a Gaussian with covariance equal to the covariance matrix of the obtained LF-NS posterior. We ran the pMCMC algorithm for 24 hours and indicated the obtained marginal posteriors in blue. As can be seen, our obtained LF-NS posterior fits very well with the posterior obtained through pMCMC. In S7 Fig we show the development of the evidence approximation as well as the corresponding standard errors and the development of the upper and lower bound estimation for the lowest achievable variance $\sigma_{\text{min}}^{2}$.

### Comparison with other likelihood-free approaches

To further evaluate and compare our LF-NS algorithm with other state-of-the-art likelihood-free approaches, we compare it to two of the most popular current methods in this field, particle MCMC (pMCMC) and Approximate Bayesian Computation—Sequential Monte Carlo (ABC-SMC), that have both been the subject of several recent review papers [27–29].

The pMCMC method (as presented for instance in [46]) is in its core a Metrpolis-Hastings MCMC (MH-MCMC) method, where the likelihood in the acceptance ratio is replaced with its particle filter approximation (for more details see S8.1 Appendix). It therefore inherits many of the strengths and weaknesses of the MH-MCMC method. Among its strengths is that it targets the true posterior P(θ), is easy to implement and has been very well studied. The downsides of pMCMC include that for realistic problems its performance depends on the initial sample *θ*_0_ of the Markov Chain as well as the choice of the perturbation kernel *q* that is used to create the Markov Chain. Further, as pMCMC produces a sequence of dependent samples it is rather difficult to parallelize and can get stuck in local regions of high-likelihood. Like our LF-NS method, the pMCMC method is the result of using an established inference method (for pMCMC it is MH-MCMC and in the case of LF-NS it is nested sampling) and replacing the likelihood computation with a particle filter approximation. Like the LF-NS method, pMCMC requires the unbiasedness of the particle filter approximation to guarantee the convergence to the true posterior.

Unlike pMCMC, ABC methods do not target the true posterior *p*(*θ*|*y*), but instead aim at obtaining an approximate posterior *p*(*θ*|*d*(*y*_*θ*_, *y*) < *ϵ*), where *y*_*θ*_ is the simulated dataset using parameter *θ*, *y* is the experimental data, *d* is some chosen distance and *ϵ* > 0 is some predefined threshold. ABC-SMC ([51]) further improves on this idea, as it creates the distribution *p*(*θ*|*d*(*y*_*θ*_, *y*) < *ϵ*) by constructing a sequence of intermediate distributions *p*(*θ*|*d*(*y*_*θ*_, *y*) < *ϵ*_*i*_), corresponding to a decreasing sequence of *ϵ*_0_ > … > *ϵ*_*F*_ = *ϵ*. In each iteration *i*, ABC-SMC samples a set of *N* particles from the distribution obtained in the previous iteration and accepts the new particles if *d*(*y*_*θ*_, *y*) < *ϵ*_*i*_ (see for details [20, 51] and S8.2). This sequential approach is very similar to our LF-NS algorithm and differs from it mainly in the way the intermediate distributions are defined. The ABC-SMC methods create them so that the distance *d*(*y*_*θ*_, *y*) lies under a certain threshold *ϵ*_*i*_, while the LF-NS algorithm requires the likelihood approximation $\hat{l}\left(\theta \right)$ to be above a certain threshold *ϵ*_*i*_.

The advantages of ABC-SMC include that, unlike pMCMC methods they create uncorrelated samples and can therefore be easily parallelized, are not in danger of getting stuck in isolated parameter regions and do not require the specification of a good initial sample. Additionally, the computation of the distance *d*(*y*_*θ*_, *y*) is usually much cheaper than running a particle filter. The obvious downsides of ABC-SMC methods is that they do not target the true posterior and depend on the definition of the distance *d* as well as a suitable sequence of thresholds *ϵ*_1_, > … >*ϵ*_*F*_. For further discussions on pMCMC and ABC methods we refer the reader to the above mentioned review papers [27–29] and S8 appendix in the supplementary.

To give an idea of the performance of these algorithms compared to our LF-NS algorithm, we implemented both the pMCMC algorithm as well as the ABC-SMC method and used them to infer the posterior distribution of the parameters of the Lac-Gfp model. As discussed earlier, this model is particularly challenging due to its large parameter space and its switch-like behavior. We ran the pMCMC method for 48 hours on the same machine as the LF-NS algorithm. To make the comparison as favorable as possible to the pMCMC method, we used the previously obtained LF-NS posterior for the Lac-Gfp model to tune the parameters of the pMCMC run. We chose as perturbation kernel *q*, a Gaussian distribution with the covariance being the sample covariance matrix of the posterior distribution of the previously obtained posterior. We used the same number of particle filter particles *H* = 500 as for the LF-NS run. We performed a total of two runs, one where the initial sample *θ*_0_ was chosen randomly from the prior *π*(*θ*) and one where it was sampled from the posterior P(θ|y) previously obtained with LF-NS. The resulting posteriors, as well as the detailed pMCMC runs are shown in S8 and S9 Figs. We observed that when choosing the initial sample *θ*_0_ from the true posterior, the pMCMC method converges to the true posterior. While we ran the pMCMC algorithm sequentially for 48 hours, this runtime can be improved by combining various approaches from the literature such as running several parallel MCMC or parallelizing the particle filter approximation. However, these modifications take a significant effort to implement and tune, and a thorough discussion of all possible option is beyond the scope of this simple comparison. When sampling *θ*_0_ from the prior distribution *π*(*θ*) (and not from the previously obtained posterior P(θ|y)), we observed that even after 48 hour the pMCMC method did not converge to the parameter region of the posterior and thus failed to give any meaningful parameter estimate (see S8 and S9 Figs). We conclude that the pMCMC method is generally capable of inferring the parameter posterior for models of size and complexity comparabvle to the Lac-Gfp model, but requires extensive tuning and an initial sample that is already in a high likelihood region. We also point out that in this example we used as the perturbation kernel the covariance of the actual posterior distribution. In a realistic setting, this perturbation kernel and the initial sample need to be determined without any knowledge of the true posterior, which proves to be considerably more difficult. Further, it is difficult to know when the pMCMC algorithm reaches the posterior distribution without the knowledge of the true distribution. This can be seen from S9 Fig, where the pMCMC with initial sample taken from the prior *θ*_0_ ∼ *π*(*θ*) seems to reach a region of high likelihood, but is in truth far away from true posterior. This issue of tuning the pMCMC method is well known and discussed in the literature. For a further discussion on the tuning problem see for instance [27, 28].

We also ran the ABC-SMC method for the Lac-Gfp model, using as distance metric the euclidean distance between the simulated dataset *y*_*θ*_ and experimental data *y*. As in [27] we created the sequence of *ϵ*_*i*_ by picking it to be the 30% quantile of the distances computed in the previous iteration. Due to its easy parallelization we ran the ABC-SMC algorithm in parallel on 48 cores for 24 hours. The resulting marginal posteriors are shown in S10 Fig. We observe that for many of the parameters (*θ*_1_-*θ*_7_, *θ*_10_, *θ*_15_–*θ*_18_), the inferred marginal posteriors correspond to the marginal posteriors obtained with the LF-NS method. For several other parameters (*θ*_8_, *θ*_9_, *θ*_11_–*θ*_14_) the inferred distributions do not seem to match the posterior distribution inferred with LF-NS. This can be due to several reasons. The ABC-SMC method may not have been run for a sufficiently long time or the final *ϵ*_*F*_ was not small enough. This result is not surprising, as the ABC-SMC method is not expected to return the true posterior. However, if the true posterior is not of interest and the modeler wishes to only get an idea about the likely location of the model parameters, the ABC-SMC method provides a viable option.

We also compared our LF-NS method with previously published results on the inference for the Lotka-Voltera model (see S7.4 Appendix) that was performed in [27]. There, the authors compared the development of the pMCMC and ABC-SMC methods on this example. We used our LF-NS algorithm to infer the posterior for the same problem. First, we compare the obtained posteriors for the three methods. As ground truth we take the posterior obtained from a very long (12 hours) pMCMC run. We ran all three algorithms sequentially for the same amount of time (12 minutes). The obtained posteriors are shown in S11 Fig. We can clearly see that the LF-NS and the pMCMC algorithms target the true posterior distribution. The ABC-SMC algorithm, while still identifying the true parameters θ*, seem to infer a distribution slightly different than the posterior distribution.

**Fig 6:**
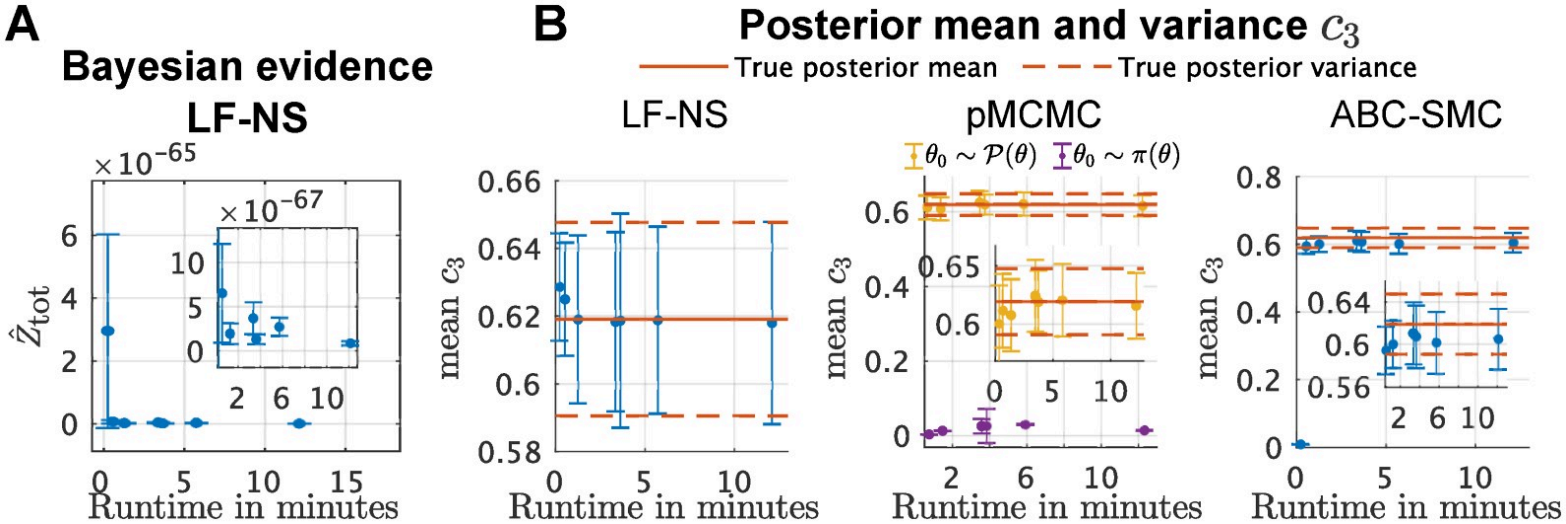
Runtime comparison between LF-NS, pMCMC and ABC. **A**: The estimated Bayesian evidence for the different LF-NS runs. The error bars indicate the standard deviation of the final BE estimate. **B**: The estimated mean and standard deviation of the marginal posterior for *c*_3_ for the different algorithm runs. The solid red line indicates the mean and standard deviation of the true marginal posterior for *c*_3_.

To compare the computational efficiency of the three algorithms, we followed [27] and used the LF-NS, the pMCMC and the ABC-SMC algorithm to infer the posteriors for the Lotka-Voltera model while limiting ourselves to comparable computational effort. We first ran the LF-NS algorithm with different number of live particles (*N* = 10, 20, 40, 60, 80, 100, 200) using as termination criteria $\Delta_{LFNS}^{m}=0.01$. The runs were performed sequentially (rather than in parallel) to make the results more comparable between the three algorithms. We ran the ABC-SMC algorithm for the same problem using the same number of particles as in the LF-NS runs. As the ABC-SMC algorithm does not provide any termination criteria, we terminate it after the same runtime as the LF-NS algorithm. We also performed several pMCMC runs, one with initial sample from the posterior distribution and one with the initial sample from the prior distribution. We ran each of the pMCMC runs for the same amount of time as the LF-NS runs took and plotted the obtained results in Fig 6.

In Fig 6A we show the development of the Bayesian evidence estimate for each of the LF-NS runs, as well as the estimator variance. Fig 6B shows the estimated marginal posterior means and variance for the parameter *c*_3_. The only algorithm that clearly failed at obtaining the true parameter mean and variance is the pMCMC run where *θ*_0_ was picked from the prior. We also see that the ABC-SMC algorithm gets very close to the true parameter mean, but consistently underestimates it, which is a strong indicator that the targeted distribution is not the true posterior. For the LF-NS algorithm as well as the pMCMC algorithm with *θ*_0_ picked from the posterior, we see that every run results in an estimate very close to the true mean and variance. For the Lotka-Voltera model, the runtime of the pMCMC as well as the infered posterior distribution depends on the initial sample. When *θ*_0_ is picked close enough to high posterior regions, the convergence is very quick, while when picked from the prior, the convergence can take very long. The speed of the converges will in general also depend on the used perturbation kernel *q* and can be greatly improved by tuning the pMCMC algorithm further. The LF-NS algorithm captures the posterior for all runtimes reliably, where larger runtimes result in a lower estimator variance. Unlike pMCMC, the LF-NS algorithm as well as the ABC-SMC algorithm both explore the full parameter space and then converge to the (approximate) posterior. Further LF-NS and ABC-SMC can easily be parallelized which will greatly improve the runtime in practical problems.

## Discussion

We have introduced a likelihood-free formulation of the well known nested sampling algorithm and have shown that it is unbiased for any unbiased likelihood estimator. While the utilization of NS for systems without an available likelihood is straight forward, one has to take precautions to avoid infeasibly high computational times. Unlike for standard NS, it is crucial to include the estimation of the live samples to the final BE estimation as well as terminate the algorithm as soon as possible. We have shown how using a Monte Carlo estimate over the live points not only results in an unbiased estimator of the Bayesian evidence *Z*, but also allows us to derive a formulation for a lower bound on the achievable variance in each iteration. This lower bound at each iteration has been shown to be a lower bound for the best achievable variance and has allowed us to formulate a novel termination criterion that stops the algorithm as soon as a continuation can at best result in an insignificant improvement in accuracy. While the formulation of the variances and its lower bound were derived having a parallel LF-NS scheme in mind, they can equally well be used in the standard NS case and can be added effortlessly to already available toolboxes such as [36] or [37]. We emphasize that the lower variance bound approximation ${\hat{\sigma }}_{\text{min}}^{2m}$ is neither a strict error term, as it only gives information of the variance of the estimator, nor a strict lower bound of the estimator variance since it contains the unknown term *L*_*m*_. Instead, it gives an estimate of the lowest achievable estimator variance that depends on the Monte Carlo estimate of the likelihood average over the live points ${\bar{L}}_{m}$. This can be seen Fig 4A and S7A Fig, where the lower bound estimate ${\hat{\sigma }}_{\text{min}}^{2m}$ does not only make jumps, but also decreases after each jump (the actual lower bound estimate $\sigma_{\text{min}}^{2m}$ is monotonically increasing in *m* as shown in S6.2 Appendix). Our suggested LF-NS scheme has three different parameters that govern the algorithm behaviour. The number of LF-NS particles *N* determines how low the minimal variance of the estimator can get, where low values for *N* result in a rather high variance and high values for *N* result in a lower variance. The number of particles for the particle filter *H* determines how wide or narrow the likelihood estimation is and thus determines the development of the acceptance rate of the LF-NS run, while the number of LF-NS iterations determines how close the variance of the final estimate comes to the minimal variance. We have demonstrated the applicability of our method on several models with simulated as well as real biological data.

We also compared the performance of the LF-NS scheme with the most widely used other likelihood-free inference schemes in the field, pMCMC and ABC-SMC. We have shown that for the considered example of the Lac-Gfp model, the ABC-SMC method failed to recover the desired posterior in any reasonable time, while the pMCMC algorithm did recover the true posterior, but only after extensive tuning and after a much longer run time than our proposed method. Further we have compared the LF-NS method with the performance of pMCMC and ABC-SMC on the Loktka-Voltera model and have demonstrated that on this example, the computational effort as well as the accuracy of the results compares very well with the other two state-of-the-art methods. Both the issues with ABC-SMC as well as the extensive tuning with pMCMC are well known and discussed in the literature. Further, we have introduced a clear termination criteria for the LF-NS method that allows to terminate the algorithm as soon as no improvement of accuracy can be expected.

We believe that our method, while in its individual elements similar to pMCMC and ABC-SMC, combines the strengths of each approach while circumventing the crucial tuning required by pMCMC and the issue of targeting an approximate rather than the true posterior distribution of the ABC-SMC.

Our LF-NS can, similarly to ABC, pMCMC or SMC models deal with stochastic models with intractable likelihoods and has all of the advantages of classic NS. We believe that particularly the variance estimation that can be performed from a single LF-NS run proves to be useful as well as the straight forward parallelization.

## Supporting information

S1 Appendix Nested sampling. (PDF)

S2 Appendix Parallelization schemes. (PDF)

S3 Appendix Sampling from the super-level set. (PDF)

S4 AppendixAccuracy of the likelihood approximation $\hat{l}$.(PDF)

S5 Appendix Estimating the variance for the parallel LF-NS scheme. (PDF)

S6 AppendixLower bound on the variance $\text{Var}(\eta_{L}^{m,r}+\eta_{D}^{m,r})$.(PDF)

S7 Appendix Examples used. (PDF)

S8 Appendix Comparison with other methods for likelihood-free Bayesian inference. (PDF)

S1 FigIllustration of the nested sampling approximation with a uniform prior on [0, 1].A: The integral over the parameter space ∫_Ω_ *l*(*θ*)*dθ*. B: The transformed integral $\int_{0}^{1}L\left(x\right)dx$ over the prior volume *x*.(PDF)

S2 FigVariance of parallel vs serial LF-NS scheme.A: Values for ${\tilde{Z}}_{D}^{i,j}$ for LF-NS run. The shaded areas indicate the standard error. B: The standard deviation of $\tilde{Z}$.(PDF)

S3 FigComparison of different sampling schemes for the super-level sets of the likelihood.A: Contour lines of the distribution of $\pi \left(\theta \right)p(\hat{l}\left(\theta \right)>\epsilon )$ for the birth death example, where *θ* = {*k*, *γ*}, the number of particle filter particles to approximate $\hat{l}\left(\theta \right)$ is *H* = 20 and log(*ϵ*) = −118.75. The density was approximated with 10^6^ samples. The red dots indicate 90 samples in L. B: The estimations of L as obtained through an ellipsoid estimation, kernel density estimation (KDE) and Dirichlet process Gaussian mixture models (DP-GMM) based on the samples in L. C: 2000 samples from the corresponding estimations of L where the samples for KDE were obtained according to (3.2) and the samples from DP-GMM were obtained using rejection sampling as described in S3 Appendix.(PDF)

S4 FigIllustration of likelihood approximation using particle filter.A: Top: Likelihood for different parameters k (red) and contour lines of the joint distribution Π(*k*, log(*k*) of the parameter *k* and its likelihood approximation $\hat{l}\left(k\right)$, based on 10^6^ samples of the likelihood approximation obtained with a particle filter with 100 particles. Bottom: The constrained priors *π*(*k*|*l*(*k*) > *ϵ*) and $\pi \left(k\right)p(\hat{l}\left(k\right)>\epsilon |k)$ for *ϵ* = 1*e* − 24. B: Acceptance rates *α*(*m*) and $Z_{D}^{m}$ from 10^6^ samples of $\Pi (k,\hat{l}\left(k\right)$ for the birth death model for different values of particle filter particles *H* and different iteration numbers *m*.(PDF)

S5 Fig The Lac-Gfp model. Schematic of the Lac-Gfp Model where the final measurement is the mature GFP (mGFP) and the input is IPTG (assumed to be constant 10*μ*M).
(PDF)

S6 Fig Lac-Gfp trajectories and likelihood approximation. A: 3 example trajectories of simulated Lac-Gfp data, measured at 29 time points. B: Log likelihood approximation for the real parameter θ* for the first trajectory with different number of particles *H* for the particle filter.
(PDF)

S7 FigEvidence and variance estimation for the transcription model.A: Development of the estimation of the Bayesian evidence using the estimation based solely on the dead points ${\hat{Z}}_{D}$, the estimate approximation from the live points ${\hat{Z}}_{L}$ and the estimation that uses both ${\hat{Z}}_{\text{tot}}$. The corresponding standard errors are indicated as the shaded areas. B: Estimate of the current variance estimate ${\hat{\sigma }}_{\text{tot}}^{2m}$ and the lower bounds for the lowest achievable variance ${\hat{\sigma }}_{\text{min}}^{2}$.(PDF)

S8 Fig Posteriors obtained from the pMCMC and ABC-SMC runs. The true parameter θ* is indicated as the blue line. The posterior obtained from the LF-NS run (as described in the main paper) is plotted as thick black line. The posteriors obtained from the two pMCMC runs are plotted in red (*θ*_0_ sampled from the posterior) and yellow (*θ*_0_ sampled from the prior).
(PDF)

S9 FigpMCMC runs for the Lac-Gfp model.pMCMC development of the individual parameters fro the two pMCMC runs with different initial samples *θ*_0_ **Left**: pMCMC run with $\theta_{0}∼P\left(\theta \right)$ **Right**: pMCMC run with *θ*_0_ ∼ *π*(*θ*).(PDF)

S10 Fig Distributions *p*(*θ*|*d*(*y*_*θ*_|*y*) < *ϵ*_*T*_) obtained from ABC-SMC. The true parameter θ* is indicated as the blue line. The marginal posterior obtained from the LF-NS run (as described in the main paper) is plotted as thick black line. The distributions *p*(*θ*|*d*(*y*_*θ*_|*y*) < *ϵ*_*F*_) are shown in thin lines.
(PDF)

S11 Fig Posteriors for the Lotka-Voltera model obtained from LF-NS, pMCMC and ABC-SMC. The obtained posteriors for all three algorithms, LF-NS, pMCMC and ABC-SMC are indicated. The posteriors were obtained using 12 minutes of computation for all three algorithms. The thick black line indicates the posterior as obtained with a long run of pMCMC.
(PDF)

S12 FigDetailed performance of each LF-NS run.Left: Evidence using the estimation based solely on the dead points ${\hat{Z}}_{D}$, the estimate approximation from the live points ${\hat{Z}}_{L}$ and the estimation based on both ${\hat{Z}}_{\text{tot}}$. The corresponding standard errors are indicated as the shaded areas. Right: Acceptance rate and cumulative runtime for each iteration.(PDF)

S1 Table Species and intial numbers of the Lac-Gfp model. (PDF)

S2 Table Prior distributions of the Lac-GFP parameters and the real values θ* used for the simulation of *y*. (PDF)

S3 Table Prior distributions of the parameters for the transcription model. (PDF)

S4 Table Species and initial numbers of the Lotka-Voltera model. (PDF)

S5 Table Prior distributions of the parameters for the Lotka-Voltera model. (PDF)
