# Supplementary material for: Likelihood-free nested sampling for parameter inference of biochemical reaction networks
Source: PLoS Comput Biol. 2020 Oct 9;16(10):e1008264. doi: 10.1371/journal.pcbi.1008264 (PMC7577508; doi:10.1371/journal.pcbi.1008264)
Supplement: S1 Appendix — (PDF) [file pcbi.1008264.s001.pdf]

S1 Nested Sampling

Nested sampling is a Bayesian inference technique that was originally introduced by John Skilling in [11] to compute the Bayesian evidence

$$Z = \int l(\theta) d\pi(\theta). \tag{1.1}$$

NS can be viewed as an importance sampling technique (as for instance discussed in [10]) as it approximates the evidence by generating samples θ_i, weights w_i and likelihoods $l_i = l(\theta_i)$ such that the weighted samples (θ_i, w_i) can be used to obtain numerical approximations of a function f over the prior π

$$\sum_i w_i f(\theta_i) \approx \int f(\theta) d\pi(\theta). \tag{1.2}$$

To compute an approximation $\widehat{Z}$ of the Bayesian evidence 1.1, f is chosen to be the likelihood function l

$$\widehat{Z} = \sum_i w_i l_i \approx \int l(\theta) d\pi(\theta). \tag{1.3}$$

The points θ_i are sampled from the prior distribution constrained to super level sets of the likelihood corresponding to an increasing sequence of thresholds. In this sense it can also be viewed as a sequential Monte Carlo method, where the intermediate distributions are the nested super level sets of the likelihood. This way, samples from NS are concentrated around the higher regions of the likelihood. One can also use the weights $l_i \times w_i$ instead of w_i to approximate functions over the posterior $\mathcal{P}(\theta)$

$$\frac{1}{\widehat{Z}} \sum f(\theta_i) l_i w_i \approx \int f(\theta) d\mathcal{P}(\theta).$$

S1.1 NS algorithm

In the following we briefly outline the NS algorithm. First, a set $\mathcal{L}_0$ of N "live" particles $\{\theta^i\}_{i=1,\dots,N}$ is sampled from the prior π

$$\theta^i \sim \pi(\theta)$$

and their likelihoods $l_i = l(\theta^i)$ are computed. Then the particle with the lowest likelihood

$$\theta_1 = \arg\min \{l(\theta) | \theta \in \mathcal{L}_0\}$$

gets removed from the set of live particles and saved together with its likelihood

$$\epsilon_1 := l(\theta_1)$$

in a set of "dead" particles $\mathcal{D}$. A new particle $\theta^\star$ is then sampled from the prior under the constraint that its likelihood is higher than ϵ_1

$$\theta^\star \sim \pi(\theta | l(\theta) > \epsilon_1). \tag{1.4}$$

This particle is combined with the remaining particles of $\mathcal{L}_0$ to form a new set of live particles $\mathcal{L}_1$ that are now distributed according to the constrained prior $\pi(\theta | l(\theta) > \epsilon_1)$, which we denote as

$$\mathcal{L}_1 \sim \pi(\theta | l(\theta) > \epsilon_1).$$

This procedure is repeated until a predefined termination criteria is satisfied. The result is a sequence of dead points θ_i with corresponding likelihoods ϵ_i that are concentrated in the regions of high likelihood. The Nested Sampling procedure is shown in Algorithm 1.

Given observations $\boldsymbol{y}$ and a prior $\pi(\theta)$ for θ.
Sample N particles θ^k from the prior π and save in the set $\mathcal{L}_0$, set $\mathcal{D} = \{\emptyset\}$
for i = 1, 2, ..., m **do**
  Set $\theta_i = \arg\min \{l(\theta)|\theta \in \mathcal{L}_{i-1}\}$ and $\epsilon_i = l(\theta_i)$
  Add $\{\theta_i, \epsilon_i\}$ to $\mathcal{D}$
  Set $\mathcal{L}_i = \mathcal{L}_{i-1} \backslash \theta_i$
  Sample $\theta^\star \sim \pi(\theta|l(\theta) > \epsilon_i)$ and add it to $\mathcal{L}_i$
end for

Algorithm 1: Nested sampling algorithm

S1.2 Approximating the Bayesian Evidence

Nested sampling exploits the fact that the Bayesian evidence 1.1 can also be written[1] (see [11]) as a one dimensional integral

$$Z = \int_0^1 L(x)dx,$$

over the prior volume

$$x(\epsilon) := \pi(l(\theta) > \epsilon) = \int_{l(\theta)>\epsilon} d\pi(\theta),$$

where $L(x)$ denotes the likelihood corresponding to the constrained prior with volume x

$$L(x) = \arg\inf_{\epsilon} \{x(\epsilon) \geq x\}. \tag{1.5}$$

We have visualized these quantities on a simple example with a uniform prior on $[0,1]$ in Figure S1.

The sampling scheme of nested sampling provides a sequence of likelihoods $\epsilon_1 < \epsilon_2 < \ldots < \epsilon_m$, but their corresponding prior volumes $x(\epsilon_i)$ are not known. However, since the ϵ_i are obtained by iteratively removing the lowest likelihood of N uniformly distributed points on the constrained prior $\pi(\theta|l(\theta) > \epsilon_{i-1})$, the prior volume $x(\epsilon_i)$ can be written as

$$x_i := x(\epsilon_i) = t^{(i)} x_{i-1},$$

where each $t^{(i)}$ is an independent sample of the random variable t which is distributed as the largest of N uniform random variables on the interval $[0,1]$ and $x_0 = 1$ (For further justification and discussion on this see [11, 4, 2] and the references within). The values $t^{(i)}$ are not known and need to be estimated. Since their distribution is known[2], they can be approximated by their means $\mathbb{E}(t) = \frac{N}{N+1}$ (or by the mean of their logs $\mathbb{E}(\log(t)) = -\frac{1}{N}$), and thus the ith prior volume can be approximated as

$$\widehat{x}_i = \left(\frac{N}{N+1}\right)^i \approx x_i. \tag{1.6}$$

With these prior volumes one can compute the importance weights w_i in equation 1.2 and 1.3 for each of the dead particles θ_i as

$$w_i = (\widehat{x}_{i-1} - \widehat{x}_i). \tag{1.7}$$

[1] for this to hold some weak conditions have to be satisfied, see for details [2] and [4]

[2] $t \sim \mathcal{B}(N, 1)$ with $\mathcal{B}(a, b)$ being the Beta distribution with parameters a and b.

These weights correct for the fact that the samples in $\mathcal{D}$ are not drawn uniformly from the prior, but are concentrated in areas of high likelihood. We note that to integrate a function on the parameter space Ω over the prior π, as in equations 1.2, only these weights are needed. To approximate Z, NS uses these weights to integrate the likelihood function $l(\theta)$ over the prior

$$Z = \int_0^1 L(x)dx \approx \sum_{i=1}^{m} L(x_i)(\widehat{x}_{i-1} - \widehat{x}_i) = \sum_{i=1}^{m} \epsilon_i w_i =: \widehat{Z}_{\mathcal{D}}^m \tag{1.8}$$

where m is the number of performed NS iterations and the subscript $\mathcal{D}$ in $\widehat{Z}_{\mathcal{D}}^m$ emphasizes that for NS the evidence estimate is obtained using only the dead points in $\mathcal{D}$. The justification for these weights as well as an in depth discussion and error approximation can be found in [2, 7, 9] and the references therein. This basic idea of nested sampling has seen several modifications and improvements over the years, along with in-depth discussions of various sampling schemes for the constrained prior [3, 5], parallel formulations [5, 6, 1] and several implementations [3, 5, 8].

S1.3 Termination of NS

Assuming that the distribution 1.4 can be efficiently sampled, each iteration of the NS scheme has the same computational complexity (the computationally most expensive step is usually to sample $\theta^\star \sim \pi(\theta | l(\theta) > \epsilon_i)$ and computing its likelihood). The NS algorithm is usually run until the remaining prior volume multiplied by the highest likelihood in this volume is smaller than a predefined fraction of the current BE estimate (see [11]). We write this quantity as

$$\Delta_{\max}^m := \widehat{x}_m \max_{\theta \in \mathcal{L}_m} (l(\theta)) \frac{1}{\widehat{Z}_{\mathcal{D}}^m}.$$

Some other termination criteria have been suggested (for instance in [5]), but since the prior volume decreases exponentially with the number of NS iterations and each iteration takes the same computational time, the choice of the particular termination criterion is not critical.

S1.4 Parallelization of NS

The parallelization of NS can be done in a very straight forward manner. Still several different parallelization schemes have been suggested in [5, 6, 1] (for a short overview see section S2). We use a parallelization scheme similar to the one presented in [6], where at each iteration not only the one particle with the lowest likelihood is resampled, but the r lowest particles. The resulting parallel scheme is outlined in Algorithm 2. With r parallel particles the final approximation 1.8 changes to

$$\widehat{Z}_{\mathcal{D}}^m = \sum_{i=1}^{m} \sum_{j=1}^{r} \epsilon_{i,j} (\widehat{x}_{i,j-1} - \widehat{x}_{i,j}), \tag{1.9}$$

with $x_{i,j} = t_j^{(i)} x_{i-1,r}$ and $t_j^{(i)}$ being i^{th} sample of t_j which is the jth largest number among N uniform numbers between 0 and 1 [3] (with the obvious boundary condition $x_{0,r} = 1$). We note that this is slightly different than the parallelization scheme presented in [5, 6, 1], for a brief discussion see S2.

[3]This means $t_j \sim \mathcal{B}(N - j + 1, j)$

1: Given observations $\boldsymbol{y}$ and a prior $\pi(\theta)$ for θ.
2: Sample N particles θ^k from the prior π and save them in the set $\mathcal{L}_0$, set $\mathcal{D} = \{\emptyset\}$
3: **for** i = 1, 2, . . . , m **do**
4:    **for** j = 1, 2, . . . , r **do**
5:       Set $\theta_{i,j} = \arg\min \{l(\theta)|\theta \in \mathcal{L}_{i-1}\}$ and $\epsilon_{i,j} = l(\theta_{i,j})$
6:       Add $\{\theta_{i,j}, \epsilon_{i,j}\}$ to $\mathcal{D}$
7:       remove $\theta_{i,j}$ from $\mathcal{L}_{i-1}$
8:    **end for**
9:    Set $\mathcal{L}_i = \mathcal{L}_{i-1}$
10:    **for** j = 1, 2, . . . , r **do**
11:       Sample $\theta^\star \sim \pi(\theta|l(\theta) > \epsilon_{i,r})$ and add it to $\mathcal{L}_i$
12:    **end for**
13: **end for**

Algorithm 2: Parallel nested sampling algorithm. The samples drawn in line 11 are all independent and thus can be drawn in parallel.

References

[1] Nikolas S Burkoff, Csilla Várnai, Stephen A Wells, and David L Wild. Exploring the energy landscapes of protein folding simulations with bayesian computation. *Biophysical journal*, 102(4):878–886, 2012.

[2] Nicolas Chopin and Christian P Robert. Properties of nested sampling. *Biometrika*, 97(3):741–755, 2010.

[3] F Feroz, MP Hobson, and M Bridges. Multinest: an efficient and robust bayesian inference tool for cosmology and particle physics. *Monthly Notices of the Royal Astronomical Society*, 398(4):1601–1614, 2009.

[4] F Feroz, MP Hobson, E Cameron, and AN Pettitt. Importance nested sampling and the multinest algorithm. *arXiv preprint arXiv:1306.2144*, 2013.

[5] WJ Handley, MP Hobson, and AN Lasenby. Polychord: next-generation nested sampling. *Monthly Notices of the Royal Astronomical Society*, 453(4):4384–4398, 2015.

[6] R Wesley Henderson and Paul M Goggans. Parallelized nested sampling. In *AIP Conference Proceedings*, volume 1636, pages 100–105. AIP, 2014.

[7] Edward Higson, Will Handley, Mike Hobson, Anthony Lasenby, et al. Sampling errors in nested sampling parameter estimation. *Bayesian Analysis*, 2018.

[8] Rob Johnson, Paul Kirk, and Michael PH Stumpf. Sysbions: nested sampling for systems biology. *Bioinformatics*, 31(4):604–605, 2015.

[9] Charles R Keeton. On statistical uncertainty in nested sampling. *Monthly Notices of the Royal Astronomical Society*, 414(2):1418–1426, 2011.

[10] Christian P Robert and Darren Wraith. Computational methods for bayesian model choice. In *AIP Conference Proceedings*, volume 1193, pages 251–262. AIP, 2009.

[11] John Skilling et al. Nested sampling for general bayesian computation. *Bayesian analysis*, 1(4):833–859, 2006.
