# Supplementary material for: Likelihood-free nested sampling for parameter inference of biochemical reaction networks
Source: PLoS Comput Biol. 2020 Oct 9;16(10):e1008264. doi: 10.1371/journal.pcbi.1008264 (PMC7577508; doi:10.1371/journal.pcbi.1008264)
Supplement: S2 Appendix — (PDF) [file pcbi.1008264.s002.pdf]

S2 Parallelization schemes

S2.1 Parallelization in [3, 6, 5]

We briefly discuss parallelization schemes for NS as they have been discussed in several other placeses like [3, 6, 5] to better illustrate the difference between our suggested scheme.

In [5] and [3] the parallel algorithm is in principle run like the sequential version, only that at each iteration i not one particle is sampled from $\pi(\theta|l(\theta) > \epsilon_i)$ but r. The key observation is that any particle $\theta^\star$ sampled from $\pi(\theta|l(\theta) > \epsilon_i)$ can also be accepted at iteration $j > i$ if $l(\theta^\star) > \epsilon_j$. Thus at each iteration i any particle for which a likelihood gets computed, beyond the first accepted particle, is used in a subsequent iteration if its likelihood is high enough.

While this provides an intuitive parallelization of the process, the r particles at each iteration i are sampled from $\pi(\theta|l(\theta) > \epsilon_i)$ but are accepted only if their respective likelihoods are higher than a sequence of increasing thresholds $\epsilon_i < \epsilon_{i+1} < \ldots < \epsilon_{i+r-1}$, which may result in discarding already sampled particles. Even despite this theoretical drawback, this method works in practice very well as demonstrated in [5]. However it seems to us wasteful to potetially discard already sample particles.

In [6] the authors suggest to parallelize NS by removing r particles with the lowest r likelihoods from the live set at each iteration i rather than just one particle. The new threshold ϵ_i is taken to be the largest likelihood of these removed r samples and since r new particles are sampled independently from the same distribution $\pi(\theta|l(\theta) > \epsilon_i)$, this is done in parallel. This results in a faster compression of the prior mass, the new particle $\theta^\star$ is sampled from the right distribution $\pi(\theta|l(\theta) > \epsilon_i)$ and the process is run in parallel. However, the authors in [6] argue that to achieve a similarly low variance of t_r as for t_1 one needs to use a higher number of NS samples N_r for the parallel NS algorithm with r parallel processes, which compares to the number of NS samples N_1 used for sequential NS through $N_r \approx \sqrt{r} N_1$ particles.

S2.2 Numerical parallelization example

In section 2.2 we described our parallelization scheme. The difference to the parallelization schemes in [6] is how we weight the particles for the evidence approximation.

We denote with t_j the random number that is distributed as the jth highest number among N uniform numbers on the interval $[0, 1]$

Assume at the beginning of iteration i the prior mass corresponding to the live particles is $x_{i-1,r}$. The prior volume shrinkage after removing $\theta_{i,1}$ is just the same as for regular nested sampling $x_{i,1} = t_1 x_{i-1,r}$, since $\theta_{i,1}$ is the particle with the lowest likelihood among N uniformly distributed particles over $\pi(\theta|l(\theta) > \epsilon_{i-1,r})$. After removing the next particle $\theta_{i,2}$ the remaining prior volume is $x_{i,2} = t_2 x_{i-1,r}$. Thus, each prior volume can be written as $x_{i,j} = t_j x_{i-1,r}$ (with the obvious boundary condition $x_{0,r} = 1$). The variance of t_r is monotonically increasing until $r = \frac{N+1}{2}$ and decreases then again, thus the variance of each t_j can be upper bound by the variance of t_r as long as $r \leq \frac{N+1}{2}$ (otherwise it can be upper bounded by the variance of $t_{r'}$ with $r' = \frac{N+1}{2}$).

We denote the Bayesian evidence approximation, using all samples $\epsilon_{i,j}$ up to $\epsilon_{m,k}$ with

$$\widetilde{Z}_{\mathcal{D}}^{m,k} = \sum_{i=1}^{m-1} \sum_{j=1}^{r} \epsilon_{i,j} (x_{i,j-1} - x_{i,j}) + \sum_{j=1}^{k} \epsilon_{m,j} (x_{m,j-1} - x_{m,j})$$

We illustrate the variance of the Bayesian evidence estimation on a small example. We assumed a likelihood function $l(\theta) = 100 \exp(-100\theta)$ with $\Omega = [0, 1]$, and ran the LF-NS algorithm for this example. In this example, the prior volume $x_{i,j}$ corresponding to the parameter $\theta_{i,j}$ is equal to this parameter $x_{i,j} = \theta_{i,j}$. In Figure S1 A we plotted the resulting values for $\widetilde{Z}_{\mathcal{D}}^{i,j}$ for each value $1 \leq i \leq m$ and $1 \leq j \leq r$. We ran $2000/r$

iterations for different values of N and r. The resulting estimations of $\widetilde{Z}_{\mathcal{D}}^{i,j}$ and the corresponding standard deviations are shown in Figure S1 A and B. As can be seen when taking the same value for N, the parallel version with $r = 20$ has a slighter higher variance. However, when increasing the number of NS particles to $N_r = N + r = 120$ the variance decreases compared to the sequential case ($r = 1$). Note that the speed up of the paralle version compared to the sequential version is a factor of r.

References

[1] Nikolas S Burkoff, Csilla Várnai, Stephen A Wells, and David L Wild. Exploring the energy landscapes of protein folding simulations with bayesian computation. *Biophysical journal*, 102(4):878–886, 2012.

[2] Nicolas Chopin and Christian P Robert. Properties of nested sampling. *Biometrika*, 97(3):741–755, 2010.

[3] F Feroz, MP Hobson, and M Bridges. Multinest: an efficient and robust bayesian inference tool for cosmology and particle physics. *Monthly Notices of the Royal Astronomical Society*, 398(4):1601–1614, 2009.

[4] F Feroz, MP Hobson, E Cameron, and AN Pettitt. Importance nested sampling and the multinest algorithm. *arXiv preprint arXiv:1306.2144*, 2013.

[5] WJ Handley, MP Hobson, and AN Lasenby. Polychord: next-generation nested sampling. *Monthly Notices of the Royal Astronomical Society*, 453(4):4384–4398, 2015.

[6] R Wesley Henderson and Paul M Goggans. Parallelized nested sampling. In *AIP Conference Proceedings*, volume 1636, pages 100–105. AIP, 2014.

[7] Edward Higson, Will Handley, Mike Hobson, Anthony Lasenby, et al. Sampling errors in nested sampling parameter estimation. *Bayesian Analysis*, 2018.

[8] Rob Johnson, Paul Kirk, and Michael PH Stumpf. Sysbions: nested sampling for systems biology. *Bioinformatics*, 31(4):604–605, 2015.

[9] Charles R Keeton. On statistical uncertainty in nested sampling. *Monthly Notices of the Royal Astronomical Society*, 414(2):1418–1426, 2011.

[10] Christian P Robert and Darren Wraith. Computational methods for bayesian model choice. In *AIP Conference Proceedings*, volume 1193, pages 251–262. AIP, 2009.

[11] John Skilling et al. Nested sampling for general bayesian computation. *Bayesian analysis*, 1(4):833–859, 2006.
