# Supplementary material for: Likelihood-free nested sampling for parameter inference of biochemical reaction networks
Source: PLoS Comput Biol. 2020 Oct 9;16(10):e1008264. doi: 10.1371/journal.pcbi.1008264 (PMC7577508; doi:10.1371/journal.pcbi.1008264)
Supplement: S3 Appendix — (PDF) [file pcbi.1008264.s003.pdf]

# S3 Sampling from the super-level set

In the course of the LF-NS algorithm the parameter-likelihood pairs $\{\theta^\star, \widehat{l}^\star\} \in \Omega \times \mathbb{R}_{\geq 0}$ need to be sampled from distributions of the form

$$\Pi(\theta, \widehat{l}(\theta)|\widehat{l}(\theta) > \epsilon) = \pi(\theta)p(\widehat{l}(\theta)|\theta, \widehat{l}(\theta) > \epsilon).$$

Since the constrained distribution of the likelihood approximation $p(\widehat{l}(\theta)|\theta, \widehat{l}(\theta) > \epsilon)$ cannot be sampled directly, we resort to rejection sampling. The parameter vector $\theta^\star$ is sampled from $\pi(\theta)$ and the likelihood approximation $\widehat{l}^\star$ is sampled from $p(\widehat{l}(\theta)|\theta)$. The pair $\{\theta^\star, \widehat{l}^\star\}$ is accepted if $\widehat{l}^\star > \epsilon$ otherwise a new $\theta^\star$ and $\widehat{l}^\star$ are being sampled. The marginal distribution of $\theta$ is

$$\theta \sim \int \Pi(\theta, \widehat{l}(\theta)|\widehat{l}(\theta) > \epsilon_i)d\widehat{l}(\theta) = \pi(\theta)p(\widehat{l}(\theta) > \epsilon). \tag{3.1}$$

Since $p(\widehat{l}(\theta) > \epsilon)$ will in general be almost zero for large areas of $\Omega$, it is important to sample $\theta^\star$ not from the full prior $\pi(\theta)$ but rather just from the prior where $p(\widehat{l}(\theta) > \epsilon)$ is larger than zero. At each iteration $i$ of the LF-NS scheme the distribution 3.1 is already given through the $N - r$ live samples in the set $\mathcal{L}$, thus the challenge is to use these samples to sample uniformly from the prior on the support of $\pi(\theta)p(\widehat{l}(\theta) > \epsilon)$. This is similar to the sampling task in standard NS where instead of distribution 3.1, the set $\pi(\theta|l(\theta) > \epsilon)$ needs to be sampled. For NS the most popular ways to sample from these distributions are slice sampling [4] and ellipsoidal sampling [1]. Unfortunately, slice sampling cannot be applied to the case of LF-NS since unlike the likelihood function $l(\theta)$ in the NS case, the function $p(\widehat{l}(\theta))$ cannot be evaluated. Also, unlike the distribution $\pi(\theta|l(\theta) > \epsilon)$, which has sharp boarders, the distribution $\pi(\theta)p(\widehat{l}(\theta) > \epsilon)$ has smooth boundaries, making density estimation techniques more appropriate than ellipsoidal sampling. In the following we illustrate three ways of performing this sampling on a small example.

- **Ellipsoid sampler** This sampler was suggested in [5] and improved upon in [2] and is used in those paper to sample the distribution $\pi(\theta|l(\theta) > \epsilon)$. The basic idea is to create an ellipsoid that encloses all of the points in $\mathcal{L}$ and then sample from this ellipsoid uniformly. This ellipsoid is usually taken to be the one being spanned by the eigenvectors of the covariance matrix of the samples in $\mathcal{L}$, scaled in a way that it encompasses all points in $\mathcal{L}$. In [2] this approach was extended by first clustering the points in $\mathcal{L}$ and only then constructing ellipsoids for each cluster. Then the samples $\theta^\star$ were sampled uniformly from the union of these ellipsoids (in this case one has to take care not to oversample the intersections of these ellipsoids).

- **Kernel density estimation (KDE)** This approach estimates the density $p(\widehat{l}(\theta) > \epsilon)$ by placing a kernel $\mathcal{K}(\cdot|\theta_i)$ on each of the points $\theta_i$ in $\mathcal{L}$. To make sure that $\theta^\star$ is sampled uniformly from the support of $p(\widehat{l}(\theta) > \epsilon)$ each kernel is weighted by $w_i = \left(\frac{1}{|\mathcal{L}|}\sum_{j=1}^{|\mathcal{L}|}\mathcal{K}(\theta_i|\theta_j)\right)^{-1}$ and thus each new particle is sampled from

$$\theta^\star \sim \left(\sum_{j=1}^{|\mathcal{L}|} w_i\right)^{-1} \sum_{j=1}^{|\mathcal{L}|} w_i \mathcal{K}(\cdot|\theta_j). \tag{3.2}$$

  With this approach the samples $\theta^\star$ are not actually sampled uniformly from the support of $p(\widehat{l}(\theta) > \epsilon)$ but as the number of points in $\mathcal{L}$ the distribution of $\theta^\star$ approaches the uniform distribution.

- **Dirichlet process Gaussian mixture models (DP-GMM)** The is the approach we are following in this paper. DP-GMM approximates the distribution of the points in $\mathcal{L}$ through a mixture of Normal distributions $\widetilde{\mathcal{L}}$

$$\mathcal{L} \sim \widetilde{\mathcal{L}} = \sum_{j=1}^{k} w_j \mathcal{N}(\cdot|\mu_j, \Sigma_j),$$

  where the number $k$, mean $\mu_j$ and shape $\Sigma_j$ of each Gaussian are estimated from the data. This is done by placing a prior distribution on the Gaussian mixture shape and form and inferring the posterior of these parameters from the data. This inference is done through iterative Gibbs sampling. The details of the DP-GMM algorithm can be found in [3]. For our algorithm runs we implemented the algorithm from [3] in C++. To sample uniformly from the estimated density we employ rejection sampling where each $\theta^\star$ gets discarded right after sampling (before we even sample $\widehat{l^\star}$) with a probability of $(1 - \frac{g}{\widetilde{\mathcal{L}}(\theta^\star)})$ and $g$ is chosen to be the 0.1% quantile of $\widetilde{\mathcal{L}}$ (if $\widetilde{\mathcal{L}}(\theta^\star)$ is below the 0.1%, $\theta^\star$ gets also rejected). While this approach is computationally more demanding than the other two approaches, in our experience it provides by far the most reliable results. The computational overhead from the inference of $\widetilde{\mathcal{L}}$ and the rejection sampling of $\theta^\star$ was in all our examples negligible compared to the total computational effort.

In Figure S1 we illustrated the different mentioned sampling schemes. As an example we took the birth death example from the main paper but this time inferred both, $k$ and $\gamma$ (thus in this case we have $\theta = \{k, \gamma\}$). We approximated the likelihood $\widehat{l}(\theta)$ using $H = 20$ particle filter particles. Figure S1 A shows the true density $\pi(\theta)p(\widehat{l}(\theta) > \epsilon)$ for $\log(\epsilon) = -118.75$. This distribution was obtained by sampling $10^6$ particles from $\pi(\theta)p(\widehat{l}(\theta) > \epsilon)$ (which was done by sampling particles from $\pi(\theta)$ and accepting them if their approximated likelihood was above $\epsilon$). The red dots indicate the 90 particles in $\mathcal{L}$. For Figure S1 B we used the Ellipsoid sampler, KDE (where each Kernel was chosen to be a Gaussian with a covariance matrix equal to the empirical covariance matrix over all points in $\mathcal{L}$) and DP-GMM to approximate the set $\mathcal{L}$. As expected, the estimation with the ellipsoid encompasses all points in $\mathcal{L}$ but is in general not very tight. The KDE and DP-GMM estimations both provide a density estimation of the set $\mathcal{L}$. Figure S1 C finally shows how the obtained candidate particles $\theta^\star$ are distributed for each of the sampling methods. For the ellipsoid these particles are just uniformly sampled from the ellipsoid, while for KDE and DP-GMM the particles were obtained as described above to guarantee that they are uniformly distributed. The red line indicates the support of the target distribution $\pi(\theta)p(\widehat{l}(\theta) > \epsilon)$ (which was approximated by plotting the envelop of the $10^6$ samples from $\pi(\theta)p(\widehat{l}(\theta) > \epsilon)$).

We also tried to run the LF-NS method with the ellipsoid sampler and the KDE sampler on the LacGfp example, but in both cases we were not able to obtain a meaningful solution. With the ellipsoid sampler the acceptance rate dropped quickly very low ($\sim 10^{-5}$ and the computational time for each iteration became unreasonably long). While the LF-NS run with the KDE sampler converged, the approximated Bayesian evidence was three orders of magnitude below our estimate with DP-GMM and inspecting the marginal posterior distributions showed that the parameter space was clearly not explored fully.

## S3.1 Final remarks regarding sampling for the contrained joint prior

We like to stress that each of the mentioned methods as well as other sampling methods such as MCMC samplers are suitable to sample from the constrained joint prior $\pi(\theta)p(\widehat{l}(\theta) > \epsilon)$. For each of these methods there exist plenty of variations that may prove particularly suitable for different cases. The brief outline in this section should be understood as a motivating illustration for the use of the DP-GMM sampler.

# References

[1] F Feroz, MP Hobson, and M Bridges. Multinest: an efficient and robust bayesian inference tool for cosmology and particle physics. *Monthly Notices of the Royal Astronomical Society*, 398(4):1601–1614, 2009.

[2] Farhan Feroz and MP Hobson. Multimodal nested sampling: an efficient and robust alternative to markov chain monte carlo methods for astronomical data analyses. *Monthly Notices of the Royal Astronomical Society*, 384(2):449–463, 2008.

[3] Dilan Görür and Carl Edward Rasmussen. Dirichlet process gaussian mixture models: Choice of the base distribution. *Journal of Computer Science and Technology*, 25(4):653–664, 2010.

[4] WJ Handley, MP Hobson, and AN Lasenby. Polychord: next-generation nested sampling. *Monthly Notices of the Royal Astronomical Society*, 453(4):4384–4398, 2015.

[5] Pia Mukherjee, David Parkinson, and Andrew R Liddle. A nested sampling algorithm for cosmological model selection. *The Astrophysical Journal Letters*, 638(2):L51, 2006.
