# Supplementary material for: Likelihood-free nested sampling for parameter inference of biochemical reaction networks
Source: PLoS Comput Biol. 2020 Oct 9;16(10):e1008264. doi: 10.1371/journal.pcbi.1008264 (PMC7577508; doi:10.1371/journal.pcbi.1008264)
Supplement: S4 Appendix — (PDF) [file pcbi.1008264.s004.pdf]

S4 Accuracy of the likelihood approximation $\widehat{l}$

As we have shown, LF-NS results in an unbiased estimation of the Bayesian evidence for any unbiased estimator of the likelihood $\widehat{l}(\theta)$. While the resulting Bayesian evidence does not depend on the particular likelihood approximation, the acceptance rate and thus the runtime of the LF-NS algorithm will in general depend on the variance of the likelihood approximation and the shape of the likelihood function. To illustrate this we used the birth-death example and sampled 10^6 particles from the distribution $\Pi(k, \widehat{l}(k))$. For each of the 10^6 samples $\{k_i, \widehat{l}_i\}$ we set $\epsilon_i = l_i$ and considered the particles $k_i^- = \min(k_j : l_j \geq \epsilon_i)$ and $k^+ = \max(k_j : l_j \geq \epsilon_i)$. We illustrated these quantities in Figure S4 A. We denote with S_i^+ all the particles between k_i^- and k_i^+ with a likelihood above ϵ_i and with S_i^- the particles with a likelihood below ϵ_i

$$S^+ = \{j : l_j > \epsilon_i, k_i^+ \geq k_j \geq k_i^-\} \quad \text{and} \quad S^- = \{j : l_j \leq \epsilon_i, k_i^+ \geq k_j \geq k_i^-\}$$

and computed the ratio of the number of their elements

$$\alpha(m) = \frac{|S_m^+|}{|S_m^-| + |S_m^+|}.$$

This quantity $\alpha(m)$ corresponds to the acceptance rate of the LF-NS algorithm with a perfect sampling scheme (a sampling scheme that samples from the support of the distribution $\pi(\theta)p(\widehat{l}(\theta) > \epsilon_i)$). We repeated this for four different values for the number of particle filter particels $H = 10$, 50, 100 and 1000 and plotted the acceptance rates in Figure S4 B. The higher the number of particles H, the lower the resulting likelihood approximation variance. As expected, as H increases, $\alpha(i)$ approaches the constant function 1. We also plotted the corresponding values for $Z_{\mathcal{D}}^m$ for different values of H as an indicator of how much information about the final Bayesian evidence the particles in the prior volumes corresponding to each ϵ_m contain. The acceptance rate will vary from problem to problem and it is in general not possible to know ahead of time what level of accuracy of the likelihood approximation is necessary for what acceptance rate. However, a low acceptance rate is a strong indicator that the variance of the likelihood approximation needs to be decreased.
