# Supplementary material for: Likelihood-free nested sampling for parameter inference of biochemical reaction networks
Source: PLoS Comput Biol. 2020 Oct 9;16(10):e1008264. doi: 10.1371/journal.pcbi.1008264 (PMC7577508; doi:10.1371/journal.pcbi.1008264)
Supplement: S5 Appendix — (PDF) [file pcbi.1008264.s005.pdf]

S5 Estimating the variance for the parallel LF-NS scheme

In the following we discuss in detail the approximation error for the Bayesian evidence

$$Z = \int_0^1 L(x)dx.$$

LF-NS approximates the above integral by approximating it on the final prior volume $x_{m,r}$ and the remaining volume separately

$$Z = \underbrace{\int_0^{x_{m,r}} L(x)dx}_{=:Z_{\mathcal{L}}^{m,r}} + \underbrace{\int_{x_{m,r}}^1 L(x)dx}_{=:Z_{\mathcal{D}}^{m,r}}.$$

The integral $Z_{\mathcal{D}}^{m,r}$ is approximated through a finite sum

$$\int_{x_{m,r}}^1 L(x)dx \approx \sum_{i=1}^m \sum_{j=1}^r L(x_{i,j})(x_{i,j-1} - x_{i,j}) = \sum_{i=1}^m \sum_{j=1}^r \epsilon_{i,j}(x_{i,j-1} - x_{i,j}) =: \widetilde{Z}_{\mathcal{D}}^{m,r}. \tag{5.1}$$

Since the prior volumes $x_{i,j}$ are in general not know, the numerical approximation $\widetilde{Z}_{\mathcal{D}}^{m,r}$ is approximated itself through the estimator

$$\widehat{Z}_{\mathcal{D}}^{m,r} = \sum_{i=1}^m \sum_{j=1}^r \epsilon_{i,j}(\widehat{x}_{i,j-1} - \widehat{x}_{i,j}) \approx \widetilde{Z}_{\mathcal{D}}^{m,r},$$

where the prior volumes $x_{i,j}$ are treated as random variables and approximated by their means $\widehat{x}_{i,j}$. The error in estimating $Z_{\mathcal{D}}$ through $\widetilde{Z}_{\mathcal{D}}^{m,r}$ is negligible compare to the error in estimating $\widetilde{Z}_{\mathcal{D}}^{m,r}$ through $\widehat{Z}_{\mathcal{D}}^{m,r}$ (see the discussion in [2] or [1]).

To emphasize its dependence on the final volume $x_{m,r}$, we rewrite the integral $Z_{\mathcal{L}}^{m,r}$ as an integral over the parameter space rather than the volume space

$$Z_{\mathcal{L}}^{m,r} = \int_0^{x_{m,r}} L(x)dx = x_{m,r} \underbrace{\int \widehat{l}(\theta) d\Pi(\theta, \widehat{l}(\theta) | \widehat{l}(\theta) \geq \epsilon_{mr})}_{=:L_{m,r}}$$

The quantity $L_{m,r}$ is the average of the likelihoods over the joint prior $\Pi(\theta, \widehat{l}(\theta))$ constraint to the likelihood regions above $\epsilon_{m,r}$. We approximate $L_{m,r}$ with a Monte Carlo estimator $\overline{L}_{m,r}$ and and the prior volume $x_{m,r}$ with its mean $\widehat{x}_{m,r}$

$$Z_{\mathcal{L}}^{m,r} \approx \widehat{x}_{m,r}\overline{L}_{m,r} =: \widehat{Z}_{\mathcal{L}}^{m,r}.$$

Thus, the final estimation error can be written as

$$\left\| Z - (\widehat{Z}_{\mathcal{D}}^{m,r} + \widehat{Z}_{\mathcal{L}}^{m,r}) \right\|$$

$$= \left\| x_{m,r} L_{m,r} - \widehat{Z}_{\mathcal{L}}^{m,r} + \int\limits_{x_{m,r}}^{1} L(x)dx - \widetilde{Z}_{\mathcal{D}}^{m,r} + \widetilde{Z}_{\mathcal{D}}^{m,r} - \widehat{Z}_{\mathcal{D}}^{m} \right\|$$

$$\leq \left\| \int\limits_{x_{m,r}}^{1} L(x)dx - \widetilde{Z}_{\mathcal{D}}^{m,r} \right\| + \left\| \underbrace{x_{m,r} L_{m,r} - \widehat{Z}_{\mathcal{L}}^{m,r}}_{=:\eta_{\mathcal{L}}^{m,r}} + \underbrace{\widetilde{Z}_{\mathcal{D}}^{m,r} - \widehat{Z}_{\mathcal{D}}^{m,r}}_{\eta_{\mathcal{D}}^{m,r}} \right\|.$$

The first part is the error from replacing the integral with a finite sum and, as mentioned before, is negligible. The errors $\eta_{\mathcal{L}}^{m,r}$ and $\eta_{\mathcal{D}}^{m,r}$ are all random variables where $\eta_{\mathcal{L}}^{m,r}$ represents the error of approximating $L_{m,r}$ with its Monte Carlo estimate $\overline{L}_{m,r}$ and replacing the final prior volume $x_{m,r}$ with its mean $\widehat{x}_{m,r}$ and the error $\eta_{\mathcal{D}}^{m,r}$ represents the error of estimating the random variables $x_{i,j}$ with their means $\widehat{x}_{i,j}$. Both errors have clearly mean 0.

As mentioned in the main paper, the prior volumes $x_{i,j}$ can be viewed as a product of the random variables t_j

$$x_{i,j} = t_j^{(i)} x_{i-1,r} = t_j^{(i)} \prod_{k=1}^{i-1} t_r^{(k)}.$$

The super script (i) in $t_j^{(i)}$ emphasizes that it is the ith sample of the random variable t_j. The random variable t_j is distributed as the jth highest number among N uniform numbers on the interval $[0,1]$, which is the Beta distribution

$$t_j \sim \mathcal{B}(N-j+1, j)$$

with

$$\widehat{t}_j := \mathbb{E}(t_j) = \frac{N-j+1}{N+1}, \quad \mathbb{E}(t_j^2) = \frac{(N-j+1)(N-j+2)}{(N+1)(N+2)}$$

and variances of

$$\mathrm{Var}\left(t_j\right) = \frac{(N-j+1)j}{(N+2)(N+1)^2}.$$

The corresponding values for the variables $x_{i,j}$ are

$$\widehat{x}_{i,j} := \mathbb{E}(x_{i,j}) = \widehat{t}_r^{i-1} \widehat{t}_j = \frac{(N-r+1)^{i-1}(N-j+1)}{(N+1)^i},$$

$$\mathbb{E}(x_{i,j}^2) = \mathbb{E}\left(t_j^{(i)^2} \prod_{k=1}^{i-1} t_r^{(k)^2} \right) = \mathbb{E}(t_j^2)\mathbb{E}(t_r^2)^{i-1} = \frac{(N-j+1)(N-j+2)(N-r+1)^{i-1}(N-r+2)^{i-1}}{(N+1)^i (N+2)^i}.$$

With this notation we compute the variances of the errors $\eta_{\mathcal{L}}^{m,r}$ and $\eta_{\mathcal{D}}^{m,r}$.

S5.1 Variance of $\eta_{\mathcal{L}}$

The error $\eta_{\mathcal{L}}$ is the error of approximating the final prior volume $x_{m,r}$ with its mean $\widehat{x}_{m,r}$ and replacing the integral L_m with its Monte Carlo estimate $\overline{L}_m$ when approximating the Bayesian evidence over the final prior volume. Its variance is

$$\mathrm{Var}(\eta_{\mathcal{L}}^{m,r}) = \mathrm{Var}(x_{m,r} L_{m,r} - \widehat{x}_{m,r} \overline{L}_{m,r}) = \mathrm{Var}\left(x_{m,r}\right) L_{m,r}^2 + \mathrm{Var}\left(\overline{L}_{m,r}\right) \widehat{x}_{m,r}^2.$$

The integral $L_{m,r}$ for the variance estimation can be estimated through the Monte Carlo estimate $\overline{L}_{m,r}$ and the variance of $\overline{L}_{m,r}$ is just

$$\mathrm{Var}\left(\overline{L}_{m,r}\right) = \frac{\sigma_L^2}{N}, \quad \sigma_L^2 = \frac{1}{N-1} \sum_{\{\theta,\widehat{l}\}\in\mathcal{L}_m} (\widehat{l} - \overline{L}_{m,r})^2.$$

The last average is taken over the points in the last live point set $\mathcal{L}_{m,r}$, which are all distributed according to $\Pi(\theta, \widehat{l}(\theta)|\widehat{l}(\theta) > \epsilon_{m,r})$.

S5.2 Variance of $\eta_{\mathcal{D}}$

Next, we estimate the variance of the estimation through the dead points. We have $\mathrm{Var}(\eta_{\mathcal{D}}) = \mathrm{Var}(\widetilde{Z}_{\mathcal{D}}^{m,r} - \widehat{Z}_{\mathcal{D}}^{m,r}) = \mathrm{Var}(\widetilde{Z}_{\mathcal{D}}^{m,r})$. We first rewrite

$$\widetilde{Z}_{\mathcal{D}}^{m,r} = \sum_{i=1}^{m}\sum_{j=1}^{r} \epsilon_{i,j}(x_{i,j-1} - x_{i,j}) = \sum_{i=1}^{m} x_{i-1,r} \underbrace{\sum_{j=1}^{r} \epsilon_{i,j}\left(t_{j-1}^{(i)} - t_j^{(i)}\right)}_{=:E_i}. \tag{5.2}$$

We point out that for each i, the samples $x_{i-1,r}$ and E_i are statistically independent since E_i only contain the ith samples of t and $x_{i-1,r} = \prod_{k=1}^{i-1} t_r^{(k)}$ contains only the samples up to $i-1$. For $m > 1$[1] we can write

$$\mathrm{Var}(\widetilde{Z}_{\mathcal{D}}^{m,r}) = \mathrm{Var}(\widetilde{Z}_{\mathcal{D}}^{m-1,r}) + 2\,\mathrm{Cov}(x_{m-1,r}E_m, \widetilde{Z}_{\mathcal{D}}^{m-1,r}) + \mathrm{Var}(x_{m-1,r}E_m). \tag{5.3}$$

We fist compute the general formula for $\mathrm{Cov}\left(x_m, \widetilde{Z}_{\mathcal{D}}^{m,r}\right)$. The product of $x_{m,r}$ and $\widetilde{Z}_{\mathcal{D}}^{m,r}$ is

$$x_{m,r}\widetilde{Z}_{\mathcal{D}}^{m,r} = \sum_{i=1}^{m} x_{m,r}x_{i-1,r}E_i$$

$$= \sum_{i=1}^{m}\prod_{l=1}^{i-1} t_r^{(l)^2} \prod_{k=i+1}^{m} t_r^{(k)} \sum_{j=1}^{r} \epsilon_{i,j}\left(t_r^{(i)}t_{j-1}^{(i)} - t_r^{(r)}t_j^{(i)}\right).$$

In the above formulation we used the convention that $\prod_{k=m}^{m-1} = 1$. With this and using that for random variables X, Y and Z we have $\mathbb{E}\left(X^2\right)\mathbb{E}\left(YZ\right) - \mathbb{E}\left(X\right)^2\mathbb{E}\left(Y\right)\mathbb{E}\left(Z\right) = \mathrm{Var}\left(X\right)\mathbb{E}(YZ) + \mathbb{E}(X)^2\,\mathrm{Cov}\left(Y,Z\right)$ we compute the covariance

$$\mathrm{Cov}(x_m, \widetilde{Z}_{\mathcal{D}}^{m}) = \sum_{i=1}^{m} \widehat{x}_{m-i,r} \sum_{j=1}^{r} \epsilon_{i,j}\left(\mathrm{Var}(x_{i-1,r})(\mathbb{E}(t_r t_{j-1}) - \mathbb{E}(t_r t_j)) + \widehat{x}_{i-1,r}^2(\mathrm{Cov}(t_r, t_{j-1}) - \mathrm{Cov}(t_r, t_j))\right) \tag{5.4}$$

[1] for $m = 1$ we obviously have $\mathrm{Var}\left(\widetilde{Z}_{\mathcal{D}}^{1}\right) = \mathrm{Var}\left(E_1\right)$.

$$= \sum_{i=1}^{m} \widehat{x}_{m-i,r} \sum_{j=1}^{r-1} \epsilon_{i,j} \operatorname{Var}\left(x_{i-1,r}\right) \left(\widehat{t}_r \widehat{t}_{j-1} - \widehat{t}_r \widehat{t}_j\right)\right)$$

$$+ \sum_{i=1}^{m} \widehat{x}_{m-i,r} \epsilon_{i,r} \left(\operatorname{Var}\left(x_{i-1,r}\right) \widehat{t}_r \widehat{t}_{r-1} - \operatorname{Var}\left(x_{i,r}\right)\right).$$

Next we compute the variance

$$\operatorname{Var}(x_{m-1,r} E_m) = \mathbb{E}\left(x_{m-1}^2\right) \operatorname{Var}(E_m) + \operatorname{Var}(x_{m-1,r}) \widehat{E}_m^2. \tag{5.5}$$

The square of E_m is

$$E_m^2 = \left(\sum_{j=1}^{r} \epsilon_{m,j} \left(t_{j-1}^{(m)} - t_j^{(m)} \right) \right)^2$$

$$= \sum_{j=1}^{r} \sum_{k=1}^{r} \epsilon_{m,j} \epsilon_{m,k} \left(t_{j-1}^{(m)} - t_j^{(m)} \right) \left(t_{k-1}^{(m)} - t_k^{(m)} \right)$$

and thus the variance of E_m is

$$\operatorname{Var}(E_m) = \sum_{j=1}^{r} \sum_{k=1}^{r} \epsilon_{m,j} \epsilon_{m,k} \operatorname{Cov}\left(t_{j-1} - t_j, t_{k-1} - t_k\right).$$

Putting this variance of E_m into formula 5.5 we obtain

$$\operatorname{Var}(x_{m-1,r} E_m) =$$

$$= \sum_{j=1}^{r} \sum_{k=1}^{r} \epsilon_{m,j} \epsilon_{m,k} \left(\mathbb{E}(x_{m-1,r}^2) \operatorname{Cov}\left(t_{j-1} - t_j, t_{k-1} - t_k\right) + \operatorname{Var}(x_{m-1,r}) \left(\widehat{t}_{j-1} - \widehat{t}_j\right) \left(\widehat{t}_{k-1} - \widehat{t}_k\right) \right)$$

$$= \sum_{j=1}^{r} \epsilon_{m,j}^2 \operatorname{Var}\left(x_{m-1,j-1} - x_{m-1,j}\right) + 2 \sum_{j=2}^{r} \sum_{k=1}^{j-1} \epsilon_{m,j} \epsilon_{m,k} (\operatorname{Var}\left(x_{m-1,r}\right) \left(1 - t_1\right))$$

$$-2 \sum_{j=2}^{r} \epsilon_{m,j} \epsilon_{m,j-1} \mathbb{E}\left(x_{m-1,r}^2\right) \operatorname{Var}\left(t_{j-1}\right)$$

Combing the variance and covariance term in 5.3 we get

$$\operatorname{Var}(\eta_{\mathcal{D}}^{m,r}) = \operatorname{Var}(\eta_{\mathcal{D}}^{m-1}) + 2 \widehat{E}_m \operatorname{Cov}(x_{m-1}, \widetilde{Z}_{\mathcal{D}}^{m-1}) + \operatorname{Var}(x_{m-1,r} E_m) \tag{5.6}$$

S5.3 Total variance

The total variance is

$$\mathrm{Var}(\eta_{\mathcal{L}}^{m,r} + \eta_{\mathcal{D}}^{m,r}) = \mathrm{Var}\left(\eta_{\mathrm{L}}^{m,r}\right) + \mathrm{Var}\left(\eta_{\mathcal{D}}^{m,r}\right) + 2\,\mathrm{Cov}\left(\eta_{\mathcal{L}}^{m,r}, \eta_{\mathcal{D}}^{m,r}\right).$$

Since $\eta_{\mathcal{L}}$ and $\eta_{\mathcal{D}}$ are not independent (they both share the random variable $x_{m,r}$), we have to account for their dependence.

$$\mathrm{Cov}(\eta_{\mathcal{L}}^{m,r}, \eta_{\mathcal{D}}^{m,r}) = \mathrm{Cov}\left(x_{m,r}L_{m,r}, \widetilde{Z}_{\mathcal{D}}^{m,r}\right) = L_{m,r}\,\mathrm{Cov}\left(x_{m,r}, \widetilde{Z}_{\mathcal{D}}^{m,r}\right)$$

This covariance can be computed using formula 5.4. The total variance is

$$\mathrm{Var}(\eta_{\mathcal{L}}^{m,r} + \eta_{\mathcal{D}}^{m,r})$$

$$= \mathrm{Var}(\eta_{\mathcal{L}}^{m,r}) + \mathrm{Var}(\eta_{\mathcal{D}}^{m,r}) + 2L_{m,r}\,\mathrm{Cov}\left(x_{m,r}, \widetilde{Z}_{\mathcal{D}}^{m,r}\right)$$

References

[1] Nicolas Chopin and Christian P Robert. Properties of nested sampling. *Biometrika*, 97(3):741–755, 2010.

[2] John Skilling et al. Nested sampling for general bayesian computation. *Bayesian analysis*, 1(4):833–859, 2006.
