# Supplementary material for: Likelihood-free nested sampling for parameter inference of biochemical reaction networks
Source: PLoS Comput Biol. 2020 Oct 9;16(10):e1008264. doi: 10.1371/journal.pcbi.1008264 (PMC7577508; doi:10.1371/journal.pcbi.1008264)
Supplement: S6 Appendix — (PDF) [file pcbi.1008264.s006.pdf]

S6 Lower bound on the variance $\mathrm{Var}(\eta_{\mathcal{L}}^{m,r} + \eta_{\mathcal{D}}^{m,r})$

The goal of this section is to establish for each iteration m a lower bound for the total variance of the NS estimator. We start by introducing some notation for the variation of the error estimate. The double index (m,k) indicates the corresponding quantities using all samples up until the (m,k) so for instance

$$\widetilde{Z}_{\mathcal{D}}^{(m,k)} = \sum_{i=1}^{m-1}\sum_{i=1}^{r} \epsilon_{i,j}(x_{i,j-1} - x_{i,j}) + \sum_{i=1}^{k} \epsilon_{m,j}(x_{m,j-1} - x_{m,j}).$$

We continue to write

$$\sigma_{\text{tot}}^{2m,j} := \mathrm{Var}(\eta_{\mathcal{L}}^{m,j} + \eta_{\mathcal{D}}^{m,j}).$$

Writing this formula out we get

$$\sigma_{\text{tot}}^{2m,j} := \ \mathrm{Var}\left(x_{m,j}\right) L_{m,j}^2 + \mathrm{Var}\left(\overline{L}_{m,j}\right) \widehat{x}_{m,j}^2 + \mathrm{Var}\left(\eta_{\mathcal{D}}^{m,j}\right) + 2L_{m,j}\,\mathrm{Cov}\left(x_{m,r}, \widetilde{Z}_{\mathcal{D}}^{m,j}\right).$$

We define with σ_{min}^{2m} the same variance, but where we replace $\mathrm{Var}\left(\overline{L}_{m,j}\right)$ with 0.

$$\sigma_{\text{min}}^{2m,j} := \ \mathrm{Var}\left(x_{m,j}\right) L_{m,j}^2 + \mathrm{Var}\left(\eta_{\mathcal{D}}^{m,j}\right) + 2L_{m,j}\,\mathrm{Cov}\left(x_{m,r}, \widetilde{Z}_{\mathcal{D}}^{m,j}\right).$$

Clearly, $\sigma_{\text{tot}}^{2m,j}$ is monotonically increasing in $\mathrm{Var}\left(\overline{L}_{m,j}\right)$ and we have for each m and each j

$$\sigma_{\text{tot}}^{2m,j} \geq \sigma_{\text{min}}^{2m,j}.$$

This is not surprising since this statement only says that the total variance at each iteration monotonically increases with the variance of the Monte Carlo estimator. As a second step we show that for all $m' \geq m$ we have

$$\sigma_{\text{min}}^{2m',j} \geq \sigma_{\text{min}}^{2m,j} \tag{6.1}$$

and for all $r \geq j' \geq j$ we have

$$\sigma_{\text{min}}^{m,j'} \geq \sigma_{\text{min}}^{m,j} \tag{6.2}$$

This statement means that assuming we know the integral of the likelihood function over the final prior volume (and thus the variance of the estimator $\overline{L}_m$ is 0), the variance of the LF-NS estimate only increases with further LF-NS iterations. Once we show this, we have shown that $\sigma_{\text{min}}^{2m,r}$ is a lower bound on the variance $\sigma_{\text{tot}}^{2m',r}$ for each iteration $m' \geq m$.

S6.1 Recursive formulations for variance terms

We show that $\sigma_{\text{min}}^{2m,j}$ is monotonically increasing in j. In the following it is understood that the double index $(m,0) = (m-1,r)$. The variance $\sigma_{\text{min}}^{2m,j}$ is

$$\sigma_{\text{min}}^{2m,j} = \ \mathrm{Var}\left(x_{m,j}L_{m,j}\right) + \mathrm{Var}\left(\eta_{\mathcal{D}}^{m,j}\right) + 2\,\mathrm{Cov}\left(x_{m,r}L_{m,j}, \widetilde{Z}_{\mathcal{D}}^{m,j}\right)$$

We first write down the recursive formulas for all three involved terms.

- $\operatorname{Var}\left(\eta_{\mathcal{D}}^{m,j}\right)$:

$$\operatorname{Var}\left(\widehat{Z}_{\mathcal{D}}^{m,j}\right) = \operatorname{Var}\left(\widetilde{Z}_{\mathcal{D}}^{m,j-1} + \epsilon_{m,j}(x_{m,j-1} - x_{m,j})\right)$$

$$= \operatorname{Var}\left(\eta_{\mathcal{D}}^{m,j-1}\right) + \epsilon_{m,j}^2 \operatorname{Var}\left(x_{m,j-1} - x_{m,j}\right) + 2\epsilon_{m,j} \operatorname{Cov}\left(x_{m,j-1} - x_{m,j}, \widetilde{Z}_{\mathcal{D}}^{m,j-1}\right).$$

- $\operatorname{Var}\left(x_{m,j}L_{m,j}\right)$: We write

$$x_{m,j}L_{m,j} = x_{m,j-1}L_{m,j-1} - (x_{m,j-1} - x_{m,j})l_{m,j},$$

where $l_{m,j}$ is the average of the likelihood over the areas where the likelihood is between $\epsilon_{m-1,j}$ and $\epsilon_{m,j}$

$$l_{m,j} = \int \widehat{l}(\theta) d\Pi(\theta, \widehat{l}(\theta) | \epsilon_{m,j} \geq \widehat{l}(\theta) \geq \epsilon_{m,j-1}).$$

This in particular implies $\epsilon_{m,j} \geq l_{m,j} \geq \epsilon_{m,j-1}$. We obtain the recursive formula

$$\operatorname{Var}(x_{m,j}L_{m,j}) = \operatorname{Var}\left(x_{m,j-1}L_{m,j-1} - (x_{m,j-1} - x_{m,j})l_{m,j}\right)$$

$$= \operatorname{Var}\left(x_{m,j-1}L_{m,j-1}\right) + \operatorname{Var}\left((x_{m,j-1} - x_{m,j})l_{m,j}\right) - 2L_{m,j-1}l_{m,j} \operatorname{Cov}\left(x_{m,j-1} - x_{m,j}, x_{m,j-1}\right)$$

- $\operatorname{Cov}\left(x_{m,j}L_{m,j}, \eta_{\mathcal{D}}^{m,j}\right)$:

$$\operatorname{Cov}\left(x_{m,j}L_{m,j}, \eta_{\mathcal{D}}^{m,j}\right) = \operatorname{Cov}\left(x_{m,j}L_{m,j}, \widehat{Z}_{\mathcal{D}}^{m,j}\right)$$

$$= \operatorname{Cov}\left(x_{m,j-1}L_{m,j-1}, \eta_{\mathcal{D}}^{m-1}\right) + \operatorname{Cov}\left(x_{m,j-1}L_{m,j-1}, \epsilon_{m,j}(x_{m,j-1} - x_{m,j})\right)$$

$$- \operatorname{Cov}\left((x_{m,j-1} - x_{m,j})l_m, \widetilde{Z}_{\mathcal{D}}^{m,j-1}\right) - \operatorname{Cov}\left((x_{m,j-1} - x_{m,j})l_m, \epsilon_{m,j}(x_{m,j-1} - x_{m,j})\right).$$

$$= \operatorname{Cov}\left(x_{m,j-1}L_{m,j-1}, \eta_{\mathcal{D}}^{m,j-1}\right)$$

$$+ L_{m,j-1}\epsilon_{m,j} \operatorname{Cov}\left(x_{m,j-1} - x_{m,j}, x_{m,j-1}\right) - l_{m,j} \operatorname{Cov}\left(x_{m,j-1} - x_{m,j}, \widetilde{Z}_{\mathcal{D}}^{m,j-1}\right)$$

$$- \epsilon_{m,j} l_m \operatorname{Var}\left(x_{m,j-1} - x_{m,j}\right).$$

S6.2 Lower bounding the residual

To obtain the full residue $\sigma_{\min}^{2m,j} - \sigma_{\min}^{2m,j-1}$ we combine the above computed residual terms:

$$\sigma_{\min}^{2m,j} - \sigma_{\min}^{2m,j-1}$$

$$= \epsilon_{m,j}^2 \operatorname{Var}\left(x_{m,j-1} - x_{m,j}\right) + 2\epsilon_{m,j} \operatorname{Cov}\left(x_{m,j-1} - x_{m,j}, \widetilde{Z}_{\mathcal{D}}^{m,j-1}\right)$$

$$+ l_{m,j}^2 \operatorname{Var}\left(x_{m,j-1} - x_{m,j}\right) - 2L_{m,j-1}l_{m,j} \operatorname{Cov}\left(x_{m,j-1} - x_{m,j}, x_{m,j-1}\right)$$

$$+2L_{m,j-1}\epsilon_{m,j}\operatorname{Cov}\left(x_{m,j-1}-x_{m,j},x_{m,j-1}\right)-2l_{m,j}\operatorname{Cov}\left(x_{m,j-1}-x_{m,j},\widetilde{Z}_{\mathcal{D}}^{m,j-1}\right)$$

$$-2\epsilon_{m,j}l_m\operatorname{Var}\left(x_{m,j-1}-x_{m,j}\right).$$

Regrouping the above terms gives us

$$\sigma_{\min}^{2m,j}-\sigma_{\min}^{2m,j-1}=2(\epsilon_{m,j}-l_{m,j})\operatorname{Cov}\left(x_{m,j-1}-x_{m,j},\widetilde{Z}_{\mathcal{D}}^{m,j-1}\right)$$

$$+2(\epsilon_{m,j}-l_{m,j})L_{m,j-1}\operatorname{Cov}\left(x_{m,j-1}-x_{m,j},x_{m,j-1}\right)$$

$$+(\epsilon_{m,j}-l_{m,j})^2\operatorname{Var}\left(x_{m,j-1}-x_{m,j}\right).$$

Since we are only interested in showing that the residual is larger than zero, it is enough to show that the sum of the first two terms is larger than zero. For ease of computation we note that for $i\leq m$ and $j<r$

$$\operatorname{Cov}\left(x_{m,j-1}-x_{m,j},x_{i-1,r}\right)=\operatorname{Cov}\left(x_{i-1,r}\prod_{k=i}^{m-1}t_r^{(k)}(t_{j-1}^{(m)}-t_j^{(m)}),x_{i-1,r}\right)=\widehat{x}_{m-i,r}\frac{1}{N+1}\operatorname{Var}\left(x_{i-1,r}\right)$$

and write for $j\leq r$

$$E_i^{(j)}=\sum_{k=1}^{j}\epsilon_{i,k}\left(t_{k-1}^{(i)}-t_k^{(i)}\right)$$

and similarly $\widehat{E}_i^{(j)}$ when we replace the $t_{k-1}^{(i)}$ and $t_k^{(i)}$with their means $\widehat{t}_{k-1}$ and $\widehat{t}_k$ respectively. We exploit the fact that $\epsilon_{m,j}\geq l_{m,j}$ and ignore the factor $2(\epsilon_{m,j}-l_{m,j})$. We proceed to write

$$\operatorname{Cov}\left(x_{m,j-1}-x_{m,j},\widetilde{Z}_{\mathcal{D}}^{m,j-1}\right)+L_{m,j-1}\operatorname{Cov}\left(x_{m,j-1}-x_{m,j},x_{m,j-1}\right)$$

$$=\sum_{i=1}^{m-1}\operatorname{Cov}\left(x_{m,j-1}-x_{m,j},x_{i-1,r}E_i^{r-1}\right)+\operatorname{Cov}\left(x_{m,j-1}-x_{m,j},x_{i-1,r}\epsilon_{i,r}t_{r-1}^{(i)}\right)-\operatorname{Cov}\left(x_{m,j-1}-x_{m,j},x_{i-1,r}\epsilon_{i,r}t_r^{(i)}\right)$$

$$+\operatorname{Cov}\left(x_{m,j-1}-x_{m,j},x_{m-1,r}E_m^{j-2}\right)+\operatorname{Cov}\left(x_{m,j-1}-x_{m,j},x_{m-1,r}\epsilon_{m,j-1}t_{j-2}^{(m)}\right)-\operatorname{Cov}\left(x_{m,j-1}-x_{m,j},x_{m,j-1}\epsilon_{m,j-1}\right)$$

$$+L_{m,j-1}\operatorname{Cov}\left(x_{m,j-1}-x_{m,j},x_{m,j-1}\right)$$

$$=\sum_{i=1}^{m-1}\widehat{x}_{m-i,r}\frac{1}{N+1}\operatorname{Var}\left(x_{i-1,r}\right)\left(\widehat{E}_i^{r-1}+\epsilon_{i,r}\widehat{t}_{r-1}\right)-\widehat{x}_{m-i-1,r}\frac{1}{N+1}\operatorname{Var}\left(x_{i,r}\right)\epsilon_{i,r}$$

$$+\frac{1}{N+1}\operatorname{Var}\left(x_{m-1,r}\right)\left(\widehat{E}_m^{j-2}+\epsilon_{m,j-1}\widehat{t}_{j-2}\right)$$

$$+\underbrace{\left(L_{m,j-1}-\epsilon_{m,j-1}\right)\operatorname{Cov}\left(x_{m,j-1}-x_{m,j},x_{m,j-1}\right)}_{\geq 0}$$

We use that $\operatorname{Var}\left(x_{0,r}\right)=0$ and $\widehat{E}_i^{(j)}+\epsilon_{i,j+1}\widehat{t}_j\geq\epsilon_{i,1}$ and write

$$\sigma_{\min}^{2m,j}-\sigma_{\min}^{2m,j-1}\geq\sum_{i=2}^{m}\widehat{x}_{m-i,r}\frac{1}{N+1}\operatorname{Var}\left(x_{i-1,r}\right)\epsilon_{i,1}-\widehat{x}_{m-i,r}\frac{1}{N+1}\operatorname{Var}\left(x_{i-1,r}\right)\epsilon_{i-1,r}\geq 0$$

With this we have shown that $\sigma_{\min}^{2m,j+1} \geq \sigma_{\min}^{2m,j}$, which means that assuming that at iteration m the integral $L_{m,r}$ over the final prior volume is known, each additional NS iteration only increases the variance. Thus $\sigma_{\min}^{2m,r}$ is a lower bound for the minimal achievable variance (using the same number N of LF-NS particles).
