# Supplementary material for: Likelihood-free nested sampling for parameter inference of biochemical reaction networks
Source: PLoS Comput Biol. 2020 Oct 9;16(10):e1008264. doi: 10.1371/journal.pcbi.1008264 (PMC7577508; doi:10.1371/journal.pcbi.1008264)
Supplement: S7 Appendix — (PDF) [file pcbi.1008264.s007.pdf]

S7 Examples used

S7.1 Birth Death Model

As described in the main paper, the birth death model consists of a single species (mRNA) that gets created at a rate k and degraded at a rate γ. This gives us two reactions

1. $\emptyset \xrightarrow{k} \text{mRNA}$ Transcription of mRNA.
2. $\text{mRNA} \xrightarrow{\gamma} \emptyset$ Degradation of mRNA.

We assume measurements $y_\tau = \mathcal{N}(\text{mRNA}(t_\tau), \sigma)$ with $\sigma = 2$. For the example in the paper we fixed the value for $\gamma = 0.1$.

S7.2 Lac-Gfp example

The second model we use for the demonstration of our algorithm is the rather large Lac-Gfp system with 9 species and 18 reactions. Table S1 shows the species involved and their initial distribution for the simulation.

The reactions of the model all follow mass action kinetics and take the following form:

1. $\emptyset \xrightarrow{\theta_1} lacI$ Transcription of lacI mRNA (constitutive).
2. $lacI \xrightarrow{\theta_2} \emptyset$ Degradation of lacI mRNA (constitutive).
3. $lacI \xrightarrow{\theta_3} lacI + LACI$ Translation of LACI protein.
4. $LACI \xrightarrow{\theta_u} \emptyset$ where $\theta_u = \theta_4 + \theta_5[IPTG]$. Degradation of LACI protein, increased by the input (IPTG).
5. $LACI + LACI \xrightarrow{\theta_6} LACI2$ Dimerization of LACI protein.
6. $LACI2 \xrightarrow{\theta_7} LACI + LACI$ Dissociation of LACI dimer.
7. $LACI2 + PLac \xrightarrow{\theta_8} O2Lac$ Binding of LACI dimer to Lac operator sequence.
8. $O2Lac \xrightarrow{\theta_9} LACI2 + PLac$ Dissociation of LACI dimer from operator sequence.
9. $O2Lac + O2Lac \xrightarrow{\theta_{10}} O4Lac$ Binding of two LacI/operator complexes and tetramerization.
10. $O4Lac \xrightarrow{\theta_{11}} O2Lac + O2Lac$ Dissociation of tetramer structure.
11. $PLac \xrightarrow{\theta_{12}} PLac + gfp$. Transcription of gfp mRNA from active Lac promoter.
12. $O2Lac \xrightarrow{\theta_{13}} O2Lac + gfp$ Transcription of gfp mRNA from Lac promoter bound to LacI dimer.
13. $O4Lac \xrightarrow{\theta_{14}} O4Lac + gfp$ Transcription of gfp mRNA from Lac promoter bound to LacI tetramer.
14. $gfp \xrightarrow{\theta_{15}} \emptyset$ Degradation of gfp mRNA.
15. $gfp \xrightarrow{\theta_{16}} gfp + GFP$ Translation of dark GFP protein.
16. $GFP \xrightarrow{\theta_{17}} \emptyset$ Degradation of dark GFP protein.
17. $GFP \xrightarrow{\theta_{18}} mGFP$ Maturation of GFP.
18. $mGFP \xrightarrow{\theta_{17}} \emptyset$ Degradation of mature GFP protein.

The effect of IPTG on the system is modelled as an increase in the degradation rate of $LACI$. The relationship between such rate and the inducer concentration, denoted $[IPTG]$, is assumed to be linear. We take the $[IPTG]$ concentration to be 10 μM.
The parameters used to simulate the dataset $\boldsymbol{y}$ as well as the priors used for the inference are shown in Table S2.

We assume that our observation y_τ at a certain time τ is distributed according to

$$y_\tau \sim \mathcal{N}(22x_\tau, 5\sqrt{x_\tau}) + \mathcal{B},$$

where x_τ is the number of GFP molecules at time t_τ, $\mathcal{N}(\mu, \sigma)$ is the normal distribution with mean μ and standard deviation σ and $\mathcal{B}$ is a known background fluorescence assumed to be $\mathcal{B} = \mathcal{N}(80, 40)$. This noise model implies a mean fluorescence of 22 for each protein and a standard deviation of 5. These model choices for the simulation are inspired by the inferred parameters for the real biological data from [1].

S7.2.1 Likelihood approximation for the Lac-Gfp system

Figure S6 A shows the first 3 simulated trajectories of the Lac-Gfp system measured on 29 timepoints. As can be seen, most trajectories exhibit switch like behaviour. This is indeed one of the properties of the considered example that make it particularly hard to perform likelihood approximation for the single trajectories. Figure S6 B shows the distribution of 100 runs of particle filter for the estimation of the log-likelihood using different numbers H of particles for the particle filter. As can be seen, the likelihood approximations vary over many thousands of orders of magnitude even with as many as $H = 5000$ particles. Observe that while the likelihood approximation is unbiased, this will usually not hold for the log-likelihood approximation.

S7.3 Transcriptional model

The third example was taken from [3] and consists of a random promoter binding at a rate k_{on} to a gene and turning it "on" and randomly unbinding from that same gene turning it "off" at a rate k_{off}. While in the "on" state, mRNA gets transcribed from that gene and can be measured as it is being transcribed. Due to the length of the gene the authors assume that the transcription takes about 2 minutes. Our model thus consists of the two species "g_{on}" and ''g_{off}" that switch between each other with the rates k_{on} and k_{off}. To account for the fact that each nascant RNA gets observed as long as it is being transcribed, we introduce 8 virtual RNA species that transform from one to another at rate λ. As initial state, we pick $g_{\text{off}} = 1$ and all other species as 0. The reactions are

1.	$g_{\text{off}} \xrightarrow{k_{\text{on}}} g_{\text{on}}$	Promoter binding to gene.
2.	$g_{\text{on}} \xrightarrow{k_{\text{off}}} g_{\text{off}}$	Promoter unbinding from gene.
3.	$g_{\text{on}} \xrightarrow{k_r} g_{\text{on}} + mRNA_1$	Transcription process
4.	$RNA_1 \xrightarrow{\lambda} RNA_2$	Transcription process
5.	$RNA_2 \xrightarrow{\lambda} RNA_3$	Transcription process
6.	$RNA_3 \xrightarrow{\lambda} RNA_4$	Transcription process
7.	$RNA_4 \xrightarrow{\lambda} RNA_5$	Transcription process
8.	$RNA_5 \xrightarrow{\lambda} RNA_6$	Transcription process
9.	$RNA_6 \xrightarrow{\lambda} RNA_7$	Transcription process
10.	$RNA_7 \xrightarrow{\lambda} RNA_8$	Transcription process
10.	$RNA_8 \xrightarrow{\lambda} \emptyset$	Transcription process

The parameters are shown in table S3. Note that the expected total transcription time is $\frac{8}{\lambda}$, this is why the prior for λ was chosen between 2 and 8.

The measurements are assumed to be the noisy read out of the total number of $mRNA$, where each $mRNA$ molecule emits fluourescence with mean $\mu = 1$ and variance $\sigma^2 = 1/8$. We also assume a background fluourescence of $\mathcal{B} = \mathcal{N}(0, 4)$. The final measurement thus reads

$$y(t) = \mathcal{N}\left(\mu \sum_{i=1}^{7} mRNA_i, \sigma^2 \sum_{i=1}^{7} mRNA_i\right) + \mathcal{B}.$$

S7.4 The Lotka-Voltera example

The Lotka-Voltera model is used to compare the LF-NS algorithm with the pMCMC and ABC-SMC methods (see S8). The model definition and settings are taken from [2] and the generated synthetic data follows the same settings as in that publication. The model describes a basic predator-prey dynamics, in which the prey species, X_1, grows with the rate c_1, a prey species gets consumed by the predator species, X_2, at rate c_2 resulting in the growth of the predator population and a predator death reaction that happens at rate c_3. The species and their initial distributions are denoted in Table S4, where Poisson$(\cdot)$ denotes the Poisson distribution.

The involved parameters, their prios as well as the simulation value has been taken from [2] and is shown in table S5.

The reaction are

1. $\emptyset \xrightarrow{c_1} X_1$ Prey birth.
2. $X_1 + X_2 \xrightarrow{c_2} X_2 + X_2$ Predatur consumtion of Prey
3. $X2 \xrightarrow{c_3} \emptyset$ Predator death

The measurement was taken to be

$$y(t) = \mathcal{N}\left(\begin{pmatrix} X_1 \\ X_2 \end{pmatrix}, \begin{bmatrix} 10^2 & 0 \\ 0 & 10^2 \end{bmatrix}\right)$$

and the dataset was simulated using 16 measuremnts at each timepoint. The initial state for the simulation as well as the true parameters are also taken from [2] and are shown in Tables S4 and S5.

References

[1] Gabriele Lillacci and Mustafa Khammash. The signal within the noise: efficient inference of stochastic gene regulation models using fluorescence histograms and stochastic simulations. *Bioinformatics*, 29(18):2311–2319, 2013.

[2] Jamie Owen, Darren J Wilkinson, and Colin S Gillespie. Likelihood free inference for markov processes: a comparison. *Statistical applications in genetics and molecular biology*, 14(2):189–209, 2015.

[3] Marc Rullan, Dirk Benzinger, Gregor W Schmidt, Andreas Milias-Argeitis, and Mustafa Khammash. An optogenetic platform for real-time, single-cell interrogation of stochastic transcriptional regulation. *Molecular cell*, 70(4):745–756, 2018.
