# Supplementary material for: Likelihood-free nested sampling for parameter inference of biochemical reaction networks
Source: PLoS Comput Biol. 2020 Oct 9;16(10):e1008264. doi: 10.1371/journal.pcbi.1008264 (PMC7577508; doi:10.1371/journal.pcbi.1008264)
Supplement: S8 Appendix — (PDF) [file pcbi.1008264.s008.pdf]

S8 Comparison with other methods for likelihood-free Bayesian inference

In order to put our LF-NS method into perspective of other approaches that tackle similar kind of problems, we follow the recent review papers on topics around likelihood-free inference ([8, 7, 3]), and compare the LF-NS method to the two most widely used likelihood-free inference methods: Approximate Bayesian Computation (ABC) and particle MCMC (pMCMC). We will briefly outline each of these methods and apply them to the previously discussed Lac-Gfp model. We also applied our LF-NS method to the Lotka-Voltera model that has also been used in [3] and compare the results with the ones obtained for pMCMC and ABC-SMC.

S8.1 pMCMC

We follow [8] and [7] and use the particle MCMC method (pMCMC) as described in [1] and [9] as our first benchmark method. The pMCMC algorithm runs the standard Metropoliton-Hastings MCMC method, but replaces the likelihood in the acceptance ratio with a particle filter approximation. Starting from an initial parameter vector (particle) θ_0, in each iteration $i = 1, \dots, N$ a new particle θ^* is proposed by sampling from a perturbation kernel q centered around the previous particle

$$\theta^\star \sim q(\cdot|\theta_{i-1}).$$

Then the likelihood $l(y|\theta^\star)$ is approximated (usually using a particle filter) with $\widehat{l}(y|\theta^\star)$ and the acceptance ratio

$$A = \frac{\pi(\theta^\star)\widehat{l}(y|\theta^\star)q(\theta_{i-1}|\theta^\star)}{\pi(\theta_{i-1})\widehat{l}(y|\theta_{i-1})q(\theta^\star|\theta_{i-1})}$$

is computed, where $\pi(\theta)$ denotes the prior distribution. With probability $\min(1, A)$ the next particle θ_i is set to $\theta^\star$ and otherwise to θ_{i-1}. The resulting pseudocode is shown in Algorithm 1

INPUT: Observations y, prior $\pi(\theta)$, proposal kernel $q(\cdot|\cdot)$, initial particle θ_0, number of required samples N
OUTPUT: samples $\{\theta_i\}_{i=1,\dots,N}$ from the posterior $\mathcal{P}(\theta)$

---

for $i = 1, \dots, N$ **do**
  Sample $\theta^\star \sim q(\cdot|\theta_{i-1})$
  Compute $\widehat{l}(y|\theta^\star)$
  Compute $A = \frac{\pi(\theta^\star)\widehat{l}(y|\theta^\star)q(\theta_{i-1}|\theta^\star)}{\pi(\theta_{i-1})\widehat{l}(y|\theta_{i-1})q(\theta^\star|\theta_{i-1})}$
  Draw uniform number $u \sim \mathcal{U}(\cdot|0,1)$
  if $u \leq \min(1, A)$ **then**
    set $\theta_i = \theta^\star$
  else
    set $\theta_i = \theta_{i-1}$
  end if
end for

Algorithm 1: pMCMC algorithm for parameter inference

In the above algorithm we denote with $\mathcal{U}(\cdot|0,1)$ the uniform distribution on the interval $[0,1]$. As has been shown in [1] and [9], under the condition that the estimator $\widehat{l}(y|\theta)$ is unbiased ($\mathbb{E}(\widehat{l}(y|\theta)) = l(y|\theta)$), the pMCMC method targets the true posterior.

S8.1.1 Comparison with LF-NS

Both the pMCMC and LF-NS algorithms rely on the same basic idea of replacing the likelihood computation in the respective underlying inference method (MH-MCMC and nested sampling) with a particle filter approximation of the likelihood and thus making the resulting method likelihood free. Both methods target the true posterior distribution under the condition that the likelihood approximation is unbiased (which is given when a particle filter is used). The comparison of the remaining features of the pMCMC and LF-NS algorithm then boil down to the comparison between standard MCMC methods and the nested sampling method. This comparison can be found in detail in other places such as [4, 2] and we will only briefly mention the main distinguishing features of the two algorithms. MCMC methods have the advantage that they are easy to implement and are guaranteed to converge to the true posterior. Further there is a wide variety of different MCMC methods available and the research on these methods is rather extensive. The commonly mentioned downsides of MCMC methods are that, as they produce a chain of dependent samples, they can get stuck in local regions of high likelihood and can usually not be parallelized in a straight forward manner. This makes it often difficult to apply MCMC methods to high-dimensional, and computationally intensive problems. Further, MCMC methods usually require the setting of various parameters, such as the perturbation kernel q, number of burn-in iterations and choice of the initial sample θ_0.

Nested sampling overcomes several of these issues, as the samples drawn are independent of each other and therefore, nested sampling methods can easily be parallelized (see section S1.4). Nested sampling algorithms also don't get stuck in local regions of high likelihood and are therefore particularly suited for multimodal target distributions. They also have the advantage that they provide a variance estimate for the estimator of the Bayesian evidence and have very few parameters that need to be set by the modeller. The main disadvantage is that they rely on being able to sample from the constrained prior

$$\pi(\theta | l(\theta) > \epsilon)$$

which indeed provides the main difficulty for nested sampling approaches and is the topic of most of the publications about nested sampling.

S8.1.2 LF-NS and pMCMC for the Lac-Gfp model

We ran a simple pMCMC algorithm on the same Lac-Gfp example that we used in the main paper (see S7.2). We used the same data and the same number of particle filter particles $H = 500$. As the perturbation kernel q, we picked the covariance matrix of the posterior obtained with the LF-NS algorithm. We found that the performance of the pMCMC method highly depends on the choice of the initial sample. We performed two runs of the pMCMC method, one where the initial sample θ_0 was randomly picked from the prior and one where we it was picked from the posterior distribution obtained with LF-NS. We ran the pMCMC algorithm for 48 hours on the same cluster that we ran the LF-NS algorithm. The sampled points for each parameter and each of the three runs are plotted in Figure S9 and the resulting posteriors in S8.

As we see in Figure S8, only the pMCMC runs with θ_0 sampled from the LF-NS posterior seems to result in a stationary distribution that resembles the one we obtained from LF-NS. When sampling θ_0 from the prior (yellow), pMCMC does not seem to be able to reach the stationary distribution and spends its entire runtime in parameter regions with very low likelihood.

This can be better seem in Figure S9, where we can also observe that the log-likelihoods from the pMCMC run with θ_0 picked from the prior get stuck at the around -2000. This is very consistend with other discussions of the pMCMC method, where it has been pointed out that some effort needs to be provided into picking the initial sample (see for instance [3]). We point out that while our LF-NS run took only 13 hours, compared to the 48 hours of pMCMC runs, this is mainly due to the easy parallelization of the LF-NS method, and the runtime of the pMCMC methods could be improved by trying to parallelize the particle filter or optimizing our simple pMCMC implementation even further. To run the pMCMC algorithm we

used already the available posterior obtained from the LF-NS runs, by picking the perturbation kernel to be the posterior covariance matrix and the θ_0 to be sampled from the posterior. In realistic scenarios, where one does not have the posterior already available, this needs to be accounted for. In [8] the authors pick the initial sample by running 4 additional MCMC chains with random initial samples before running the actual MCMC algorithm and in [3] the authors suggest to run a ABC-SMC algorithm before the actual pMCMC run. For a thorough discussion of these challenges see [3].

S8.2 Approximate Bayesian Computation

The second popular likelihood-free algorithm is the Approximate Baysian Computation (ABC), which approximate the posterior $p(\theta|y)$ with the approximate posterior $p(\theta|d(y_\theta, y) < \epsilon)$ for some $\epsilon > 0$, where y_θ denotes a dataset obtained through simulation of the system using the parameter θ and $d(\cdot,\cdot)$ defines a distance metric between the simulated dataset and the experimental data y. For our comparison we follow again [3] and [8] and use the ABC-SMC algorithm [5]. The ABC-SMC algorithm constructs a sequence of distributions $p(\theta|d(y_\theta, y) < \epsilon_k)$ for a decreasing sequence of ϵ_k.

In the first iteration the ABC-SMC algorithm picks samples from the prior $\theta^* \sim \pi(\cdot)$, simulates the system to obtain a realization y_{θ^*} and computes the distance $d(y_{\theta^*}, y)$. If this distance is lower than the initial threshold ϵ_0 the particle θ^* is accepted. This sampling process is repeated until N samples $\{\theta_i^0\}_{i=1,\dots,N}$ are accepted. In each successive iteration i this procedure is repeated, only that the new particles θ^* is not samples from the prior, but is obtained by picking a random particle from the previous population and perturbing it through a perturbation kernel q. In each iteration i a new threshold $\epsilon_i < \epsilon_{i-1}$ is used. The algorithm is often terminated after reaching a final threshold ϵ_F. The particular form of the perturbation kernel q, the metric d as well as the construction of the threshold sequence ϵ_k are left to the modeler and several different choices have been discussed in the literature (see for instance [6, 5, 8]). We illustrated the pseudocode of a ABC-SMC scheme in Algorithm 2

The weights w_k^i in Algorithm 2 are used to avoid to propagate the sampling bias introduced by the use of the kernel $q(\cdot|\cdot)$. We point out the samples θ^* in iteration i are effectively sampled from a kernel-density estimation (KDE) of the previous particles set $\{\theta_k^{i-1}\}_{k=1,\dots,N}$ as described in S3.

S8.2.1 Comparison with LF-NS

The ABC-SMC algorithm is in many aspects very similar to our proposed LF-NS algorithm. Both algorithms propagate particles through a sequence of decreasing subspaces of the parameter space, by using each previous subspace to sample proposal particles for the next. As ABC-SMC also produces independent samples it is also straight forward to parallelize. Indeed, we can recover the LF-NS algorithm by making small changes in the Algorithm 2, by replacing the computation of the distance metric $d(y_{\theta^*}, y)$ with the particle filter approximation of $\widehat{l}(\theta^*)$ and replacing the acceptance criteria $d(y_{\theta^*}, y) < \epsilon_i$ with $\widehat{l}(\theta^*) > \epsilon_i$, where for the LF-NS case the sequence $\epsilon_1, \dots, \epsilon_F$ is just the sequence of increasing likelihood thresholds. This similarity between the ABC-SMC and LF-NS algorithm results in very similar computational demands, and in general we assume the runtime of LF-NS and ABC-SMC to be very similar on similar problems. In many cases the ABC-SMC method will be computationally even cheaper, as the computation of the distance $d(y_\theta, y)$ is often cheaper than computing the likelihood approximation $\widehat{l}(\theta)$.

However, we would like to point out several downsides of the ABC-SMC algorithm that we believe our proposed LF-NS algorithm can improve on. The targeted distribution for ABC-SMC methods is not the posterior distribution $p(\theta|y)$, but rather an approximation $p(\theta|d(y_\theta, y) < \epsilon_F)$. There is in general no way of telling how close this approximation is to the true posterior. Further, the choices of distance d as well as the perturbation kernel q need to be done by the modeler and it is not always straight forward how to make these choices, especially when dealing with stochastic systems. It is also difficult to decide when to terminate ABC-SMC as the final threshold ϵ_F needs to be chosen depending on each problem and the

INPUT: Observations y, prior $\pi(\theta)$, proposal kernel $q(\cdot|\cdot)$, sequence of decreasing thresholds $\epsilon_0 > \ldots > \epsilon_F$
OUTPUT: samples $\{\theta_i^F\}_{i=1,\ldots,N}$ from the approximate posterior $p(\theta|d(y_\theta), y) < \epsilon_F)$

---

for $i = 1, \ldots, F$ **do**
  set $j = 1$
  while $j \leq N$ **do**
    if $i = 0$ **then**
      Sample $\theta^* \sim \pi(\cdot)$
    else
      Sample c according to weights $\{w_n^{i-1}\}_{n=1,\ldots,N}$
      Sample $\theta^* \sim q(\cdot|\theta_c^{i-1})$
    end if
    Simulate y_{θ^*}
    if $d(y_{\theta^*}, y) < \epsilon_i$ **then**
      set $\theta_j^i = \theta^*$
      set $j = j + 1$
    end if
  end while
  for $n = 1, \ldots, N$ compute the weights $w_n^i = \frac{\pi(\theta_n^i)}{\sum_{l=1}^{N} q(\theta_n^i|\theta_l^{i-1})}$
  for $n = 1, \ldots, N$ set $w_n^i = \frac{w_n^i}{\sum_{l=1}^{N} w_l^i}$
end for

Algorithm 2: ABC-SMC algorithm for parameter inference

chosen distance d. In practice this often means that the ABC-SMC algorithm is run for a fixed time after which the last value for ϵ_i is chosen to be the final one.

The LF-NS approach overcomes these issues by using an unbiased likelihood approximation $\widehat{l}$ instead of the distance d. While the computation of $\widehat{l}$ might often be computationally more demanding than the simulation of y_θ and computation of $d(y_\theta, d)$, it allows the LF-NS algorithm to target the true posterior $p(\theta|y)$. Further, while in the ABC-SMC algorithm the intermediate particle sets are only used to sample the next particle set more efficiently, the LF-NS algorithm uses the information about the intermediate sets and the decrease in their volume to approximate the posterior. We note that the sampling procedure for ABC-SMC resembles a KDE approximation of each particle set and is comparable with the challenge of nested sampling methods to sample from the intermediate super level sets.

S8.2.2 LF-NS and ABC-SMC for the Lac-Gfp model

We ran our own implementation of a ABC-SMC method on the Lac-Gfp model used in the main paper. To compute the distance measure $d(y_\theta|y)$, we simulate the system once to obtain the simulated trajectory y_θ and computed d to be the mean square distance between the simulation and the data

$$d(y_\theta, y) = \sum_{i=1}^{T} (y_\theta^i - y^i)^2,$$

where y^i denotes the ith measurement in the time-series y. As the perturbation kernel q we picked a Gaussian kernel, with covariance matrix being the sample covariance matrix of the previous particle set. The thresholds ϵ_i were chosen to be the the 30%-quantile of the computed distances in each iteration. We ran the ABC-SMC method on the same cluster as the LF-NS method in parallel with 48 cores. We plotted the obtained marginal approximate distributions $p(\theta|d(y_\theta|y) < \epsilon_F)$ for different iterations F in Figure S10.

As we see, several parameters like θ_{15}, θ_{16}, θ_{17} and θ_{18} have been identified correctly. Also the obtained marginal distributions for parameters θ_1 to θ_7 seem to be identified correctly. However, the obtained approximative marginal distributions for several parameters do not seem to have been identified correctly. This can be due to several reasons. It could simply be that we did not let the algorithm run long enough and that the true posterior is approximated better at even smaller levels of ϵ_F. The other reason might be that our distance measure $d(y_\theta|y)$ is not informative enough to target the true posterior. We note that this issue of not targeting the true posterior is a well-known and expected feature of ABC-SMC methods and are discussed in many other places as well (see for instance the above mentioned review papers [3]).

S8.3 Compiaring LF-NS with pMCMC and ABC-SMC on the Lotka-Voltera model

It is generally difficult to provide an adequate comparison of computational effort for different methods, as the runtime of any algorithm will always depend on the particular algorithm settings, the considered problem, the particular implementational details (and optimization) of the algorithms and also on the used hardware used to run the examples. To give an idea of the relative performance of the LF-NS algorithm compared to ABC-SMC and pMCMC methods, we therefore ran it on the same Lotka-Voltera model that was used in [3] to compare pMCMC and ABC-SMC methods. We used the model definition as in [3] and created simulated data using the same parameters and simulation protocol that was used in that paper. The detail of the model can be found in **??**. To stay as true as possible to the conditions used in [3], we ran the LF-NS algorithm sequentially (rather than in parallel). For the particle filter we used $H = 100$. In Figure S11 we show the obtained posterior for different number of live points N, where we picked $r = 0.1N$.

We plotted the posterior distribution that we obtained by running the pMCMC algorithm for the same problem, picking θ_0 randomly from the posterior obtained with LF-NS and using the sample covariance matrix of the posterior as the perturbation kernel q. We see that the LF-NS algorithm targets the same

posterior distribution as the pMCMC method. The true value is indicated in blue, while the thick black line indicates the shape of the true posterior as obtained from a single long ($\sim$12 hours) pMCMC run. As expected, the true value θ^* lies in the support of the posterior. To illustrate the development of runtime for the LF-NS algorithm, we performed 7 different runs of LF-NS, picking N = 10, 20, 40, 60, 80, 100, 200,. In Figure S12 we plotted the developments of the Bayesian evidence estimation and its variance for each of the inference runs, as well as the computational time needed and the acceptance rates for each run.

We see that the LF-NS algorithm estimates the same Bayesian evidence for each run and the variance is clearly decreasing for increasing N.

References

[1] Christophe Andrieu, Arnaud Doucet, and Roman Holenstein. Particle markov chain monte carlo methods. *Journal of the Royal Statistical Society: Series B (Statistical Methodology)*, 72(3):269–342, 2010.

[2] Iain Murray. *Advances in Markov chain Monte Carlo methods.* PhD thesis, Citeseer, 2007.

[3] Jamie Owen, Darren J Wilkinson, and Colin S Gillespie. Likelihood free inference for markov processes: a comparison. *Statistical applications in genetics and molecular biology*, 14(2):189–209, 2015.

[4] Nick Pullen and Richard J Morris. Bayesian model comparison and parameter inference in systems biology using nested sampling. *PloS one*, 9(2):e88419, 2014.

[5] Scott A Sisson, Yanan Fan, and Mark M Tanaka. Sequential monte carlo without likelihoods. *Proceedings of the National Academy of Sciences*, 104(6):1760–1765, 2007.

[6] Tina Toni, David Welch, Natalja Strelkowa, Andreas Ipsen, and Michael PH Stumpf. Approximate bayesian computation scheme for parameter inference and model selection in dynamical systems. *Journal of the Royal Society Interface*, 6(31):187–202, 2009.

[7] David J Warne, Ruth E Baker, and Matthew J Simpson. Simulation and inference algorithms for stochastic biochemical reaction networks: from basic concepts to state-of-the-art. *Journal of the Royal Society Interface*, 16(151):20180943, 2019.

[8] David J Warne, Ruth E Baker, and Matthew J Simpson. A practical guide to pseudo-marginal methods for computational inference in systems biology. *Journal of theoretical biology*, page 110255, 2020.

[9] Darren J Wilkinson. Parameter inference for stochastic kinetic models of bacterial gene regulation: a bayesian approach to systems biology. In *Proceedings of 9th Valencia International Meeting on Bayesian Statistics*, pages 679–705, 2010.
