# Supplementary figures and images for: Likelihood-free nested sampling for parameter inference of biochemical reaction networks
Source: PLoS Comput Biol. 2020 Oct 9;16(10):e1008264. doi: 10.1371/journal.pcbi.1008264 (PMC7577508; doi:10.1371/journal.pcbi.1008264)

**A**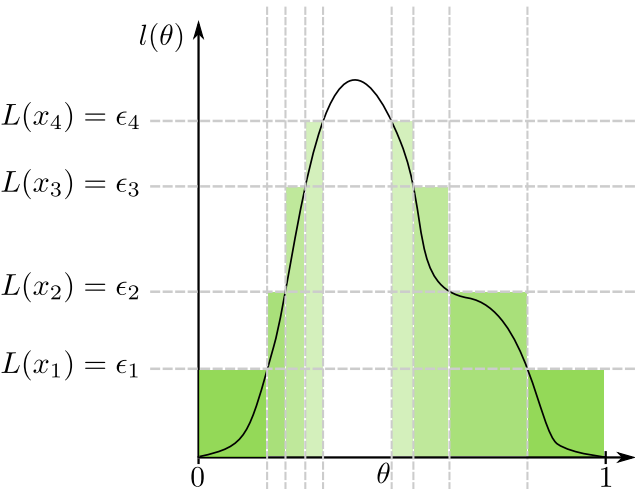

$$x_0 = 1$$

$$x_1 = x(\epsilon_1)$$

$$x_2 = x(\epsilon_2)$$

$$x_3 = x(\epsilon_3)$$

$$x_4 = x(\epsilon_4)$$

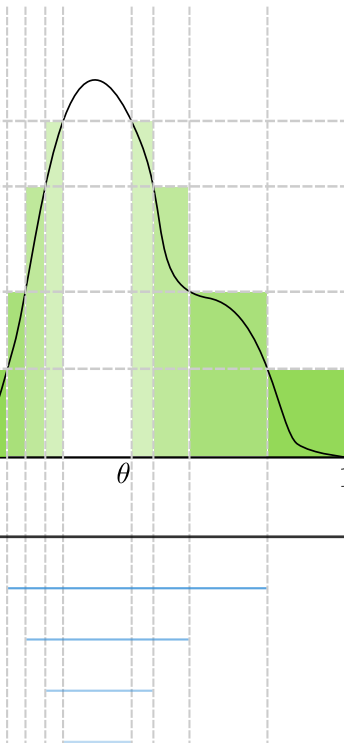**B**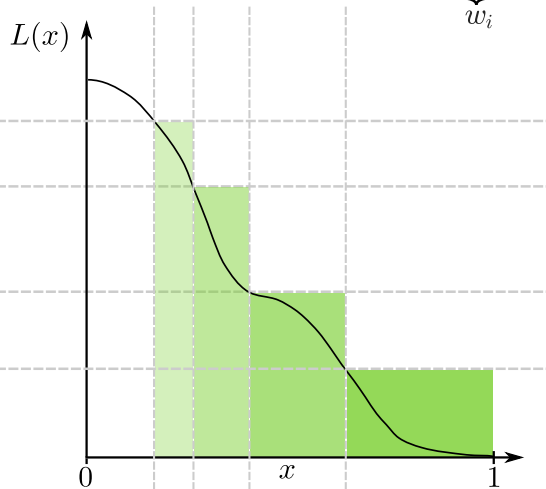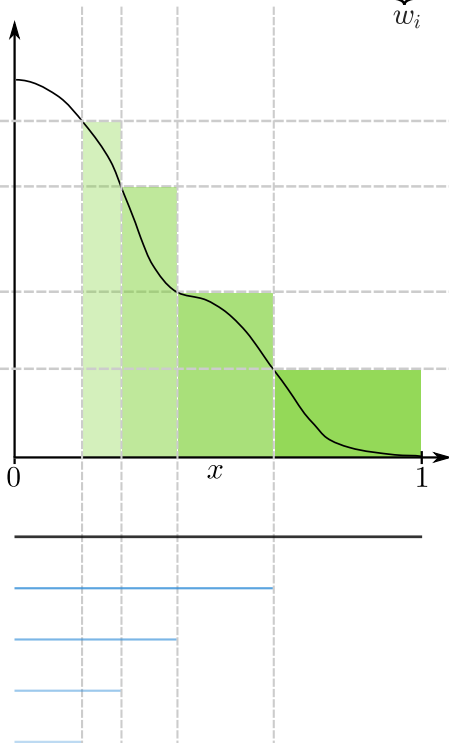

Supplement: S1 Fig — A: The integral over the parameter space ∫Ω l(θ)dθ. B: The transformed integral ∫01L(x)dx over the prior volume x. (PDF) [file pcbi.1008264.s009.pdf]

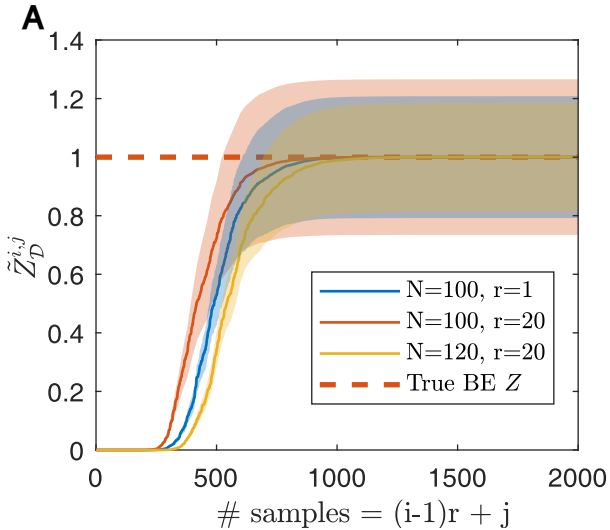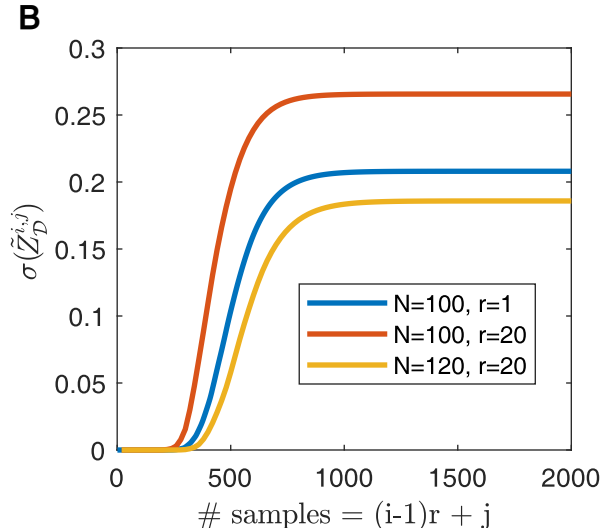

Supplement: S2 Fig — A: Values for Z˜Di,j for LF-NS run. The shaded areas indicate the standard error. B: The standard deviation of Z˜. (PDF) [file pcbi.1008264.s010.pdf]

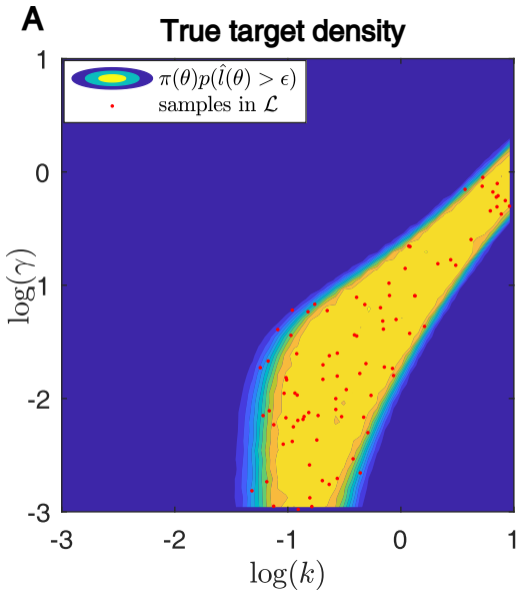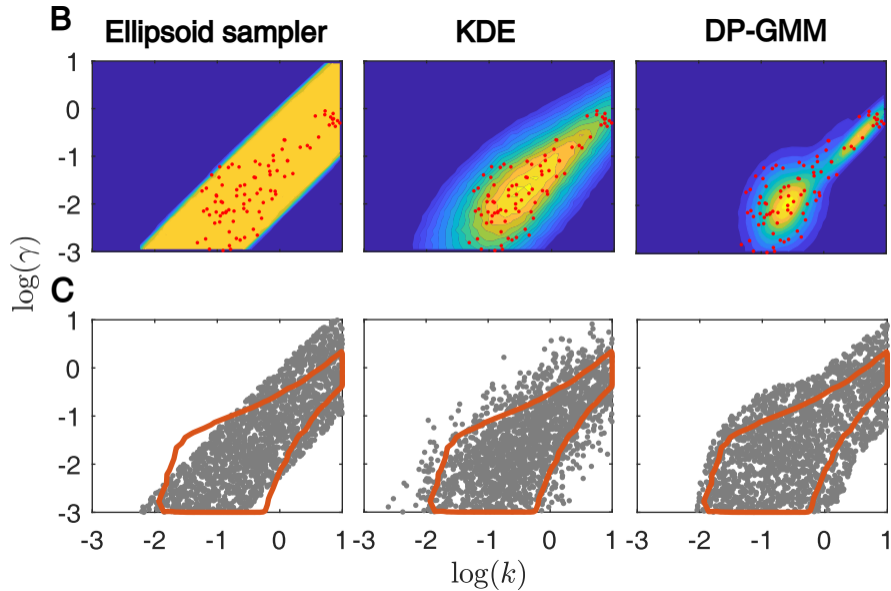

Supplement: S3 Fig — A: Contour lines of the distribution of π(θ)p(l^(θ)>ϵ) for the birth death example, where θ = {k, γ}, the number of particle filter particles to approximate l^(θ) is H = 20 and log(ϵ) = −118.75. The density was approximated with 106 samples. The red dots indicate 90 samples in L. B: The estimations of L as obtained through an ellipsoid estimation, kernel density estimation (KDE) and Dirichlet process Gaussian mixture models (DP-GMM) based on the samples in L. C: 2000 samples from the corresponding estimations of L where the samples for KDE were obtained according to (3.2) and the samples from DP-GMM were obtained using rejection sampling as described in S3 Appendix. (PDF) [file pcbi.1008264.s011.pdf]

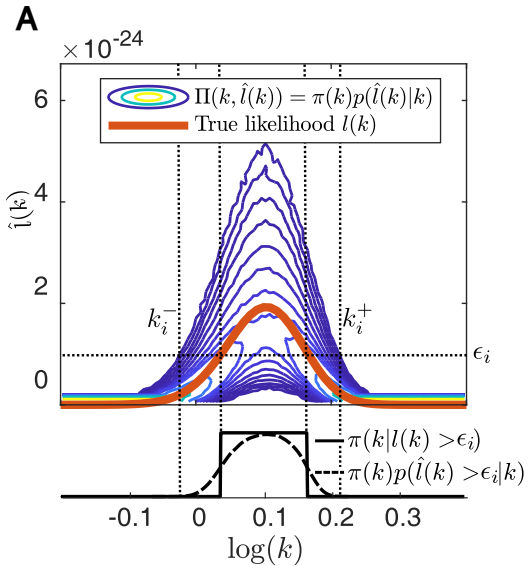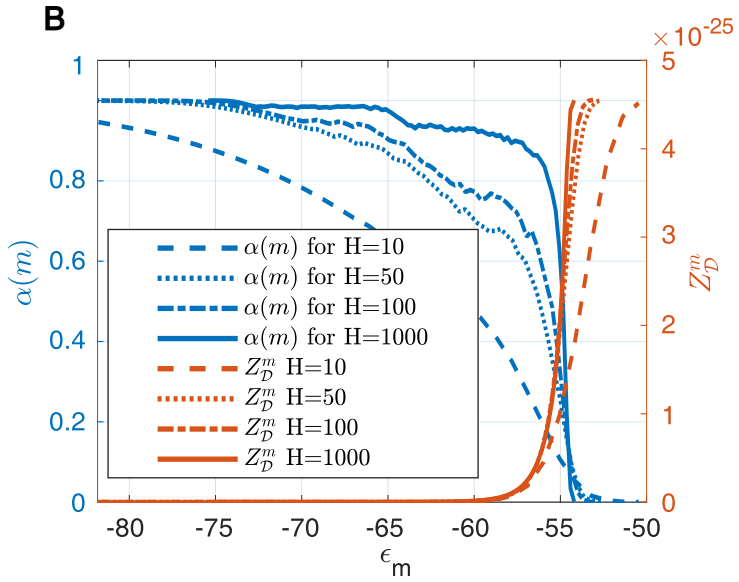

Supplement: S4 Fig — A: Top: Likelihood for different parameters k (red) and contour lines of the joint distribution Π(k, log(k) of the parameter k and its likelihood approximation l^(k), based on 106 samples of the likelihood approximation obtained with a particle filter with 100 particles. Bottom: The constrained priors π(k|l(k) > ϵ) and π(k)p(l^(k)>ϵ|k) for ϵ = 1e − 24. B: Acceptance rates α(m) and ZDm from 106 samples of Π(k,l^(k) for the birth death model for different values of particle filter particles H and different iteration numbers m. (PDF) [file pcbi.1008264.s012.pdf]

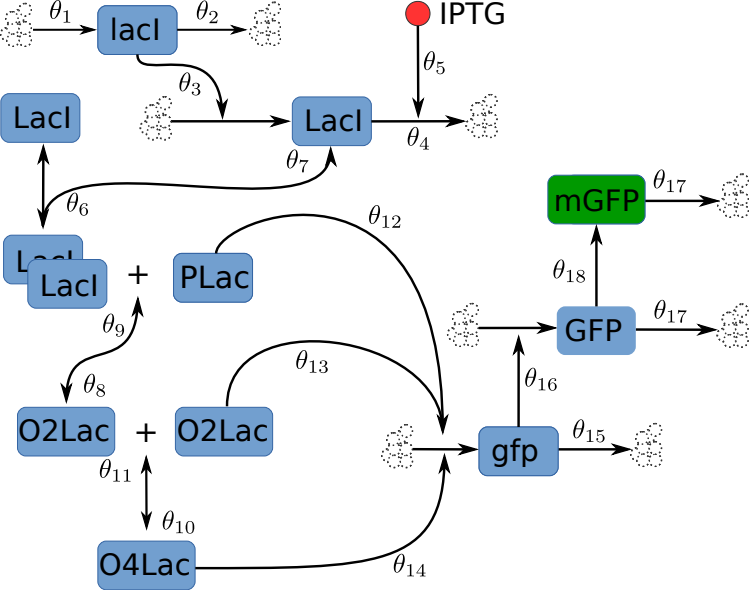

Supplement: S5 Fig — Schematic of the Lac-Gfp Model where the final measurement is the mature GFP (mGFP) and the input is IPTG (assumed to be constant 10μM). (PDF) [file pcbi.1008264.s013.pdf]

**A**

Simulated Fluorescence

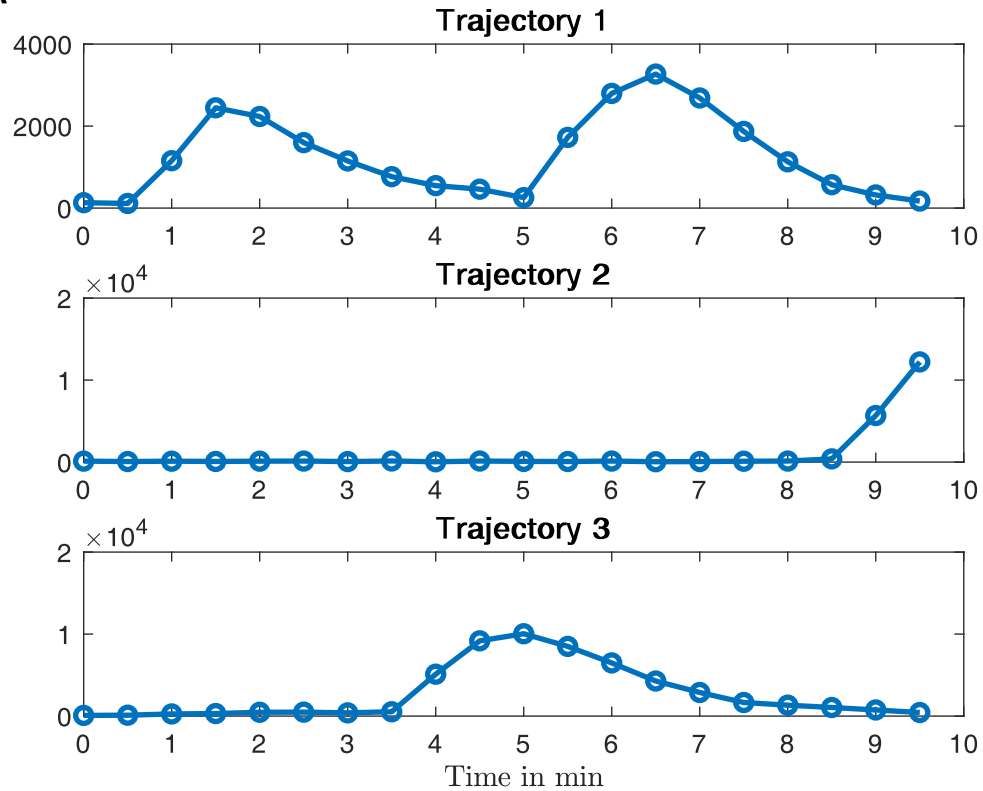**B**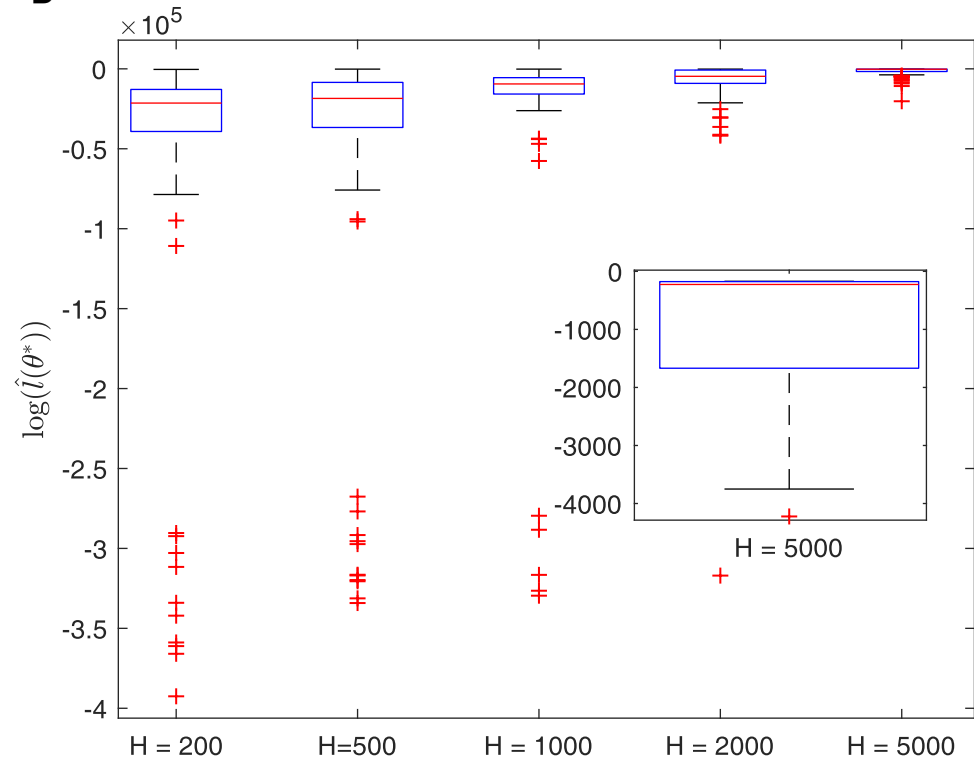

Supplement: S6 Fig — A: 3 example trajectories of simulated Lac-Gfp data, measured at 29 time points. B: Log likelihood approximation for the real parameter θ* for the first trajectory with different number of particles H for the particle filter. (PDF) [file pcbi.1008264.s014.pdf]

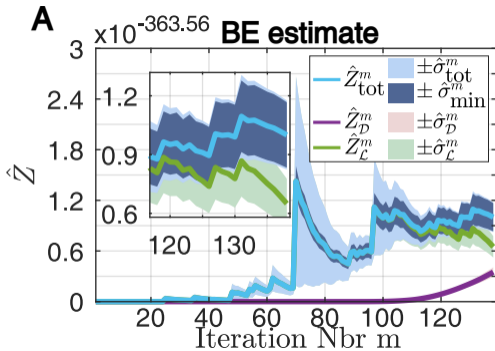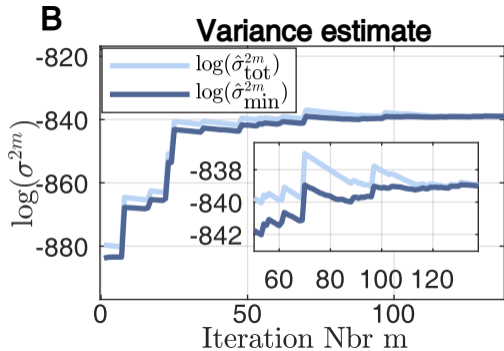

Supplement: S7 Fig — A: Development of the estimation of the Bayesian evidence using the estimation based solely on the dead points Z^D, the estimate approximation from the live points Z^L and the estimation that uses both Z^tot. The corresponding standard errors are indicated as the shaded areas. B: Estimate of the current variance estimate σ^tot2m and the lower bounds for the lowest achievable variance σ^min2. (PDF) [file pcbi.1008264.s015.pdf]

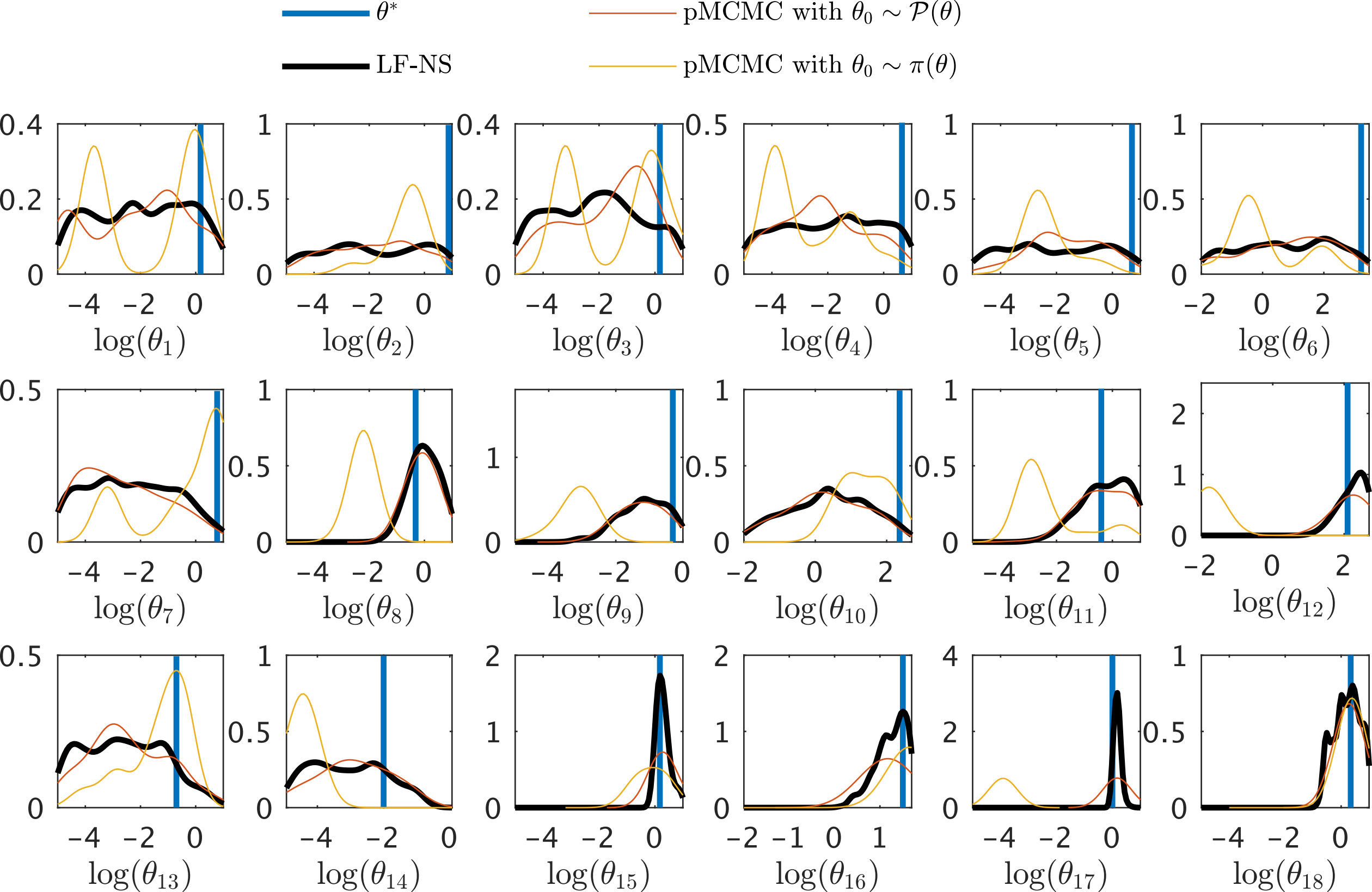

Supplement: S8 Fig — The true parameter θ* is indicated as the blue line. The posterior obtained from the LF-NS run (as described in the main paper) is plotted as thick black line. The posteriors obtained from the two pMCMC runs are plotted in red (θ0 sampled from the posterior) and yellow (θ0 sampled from the prior). (PDF) [file pcbi.1008264.s016.pdf]

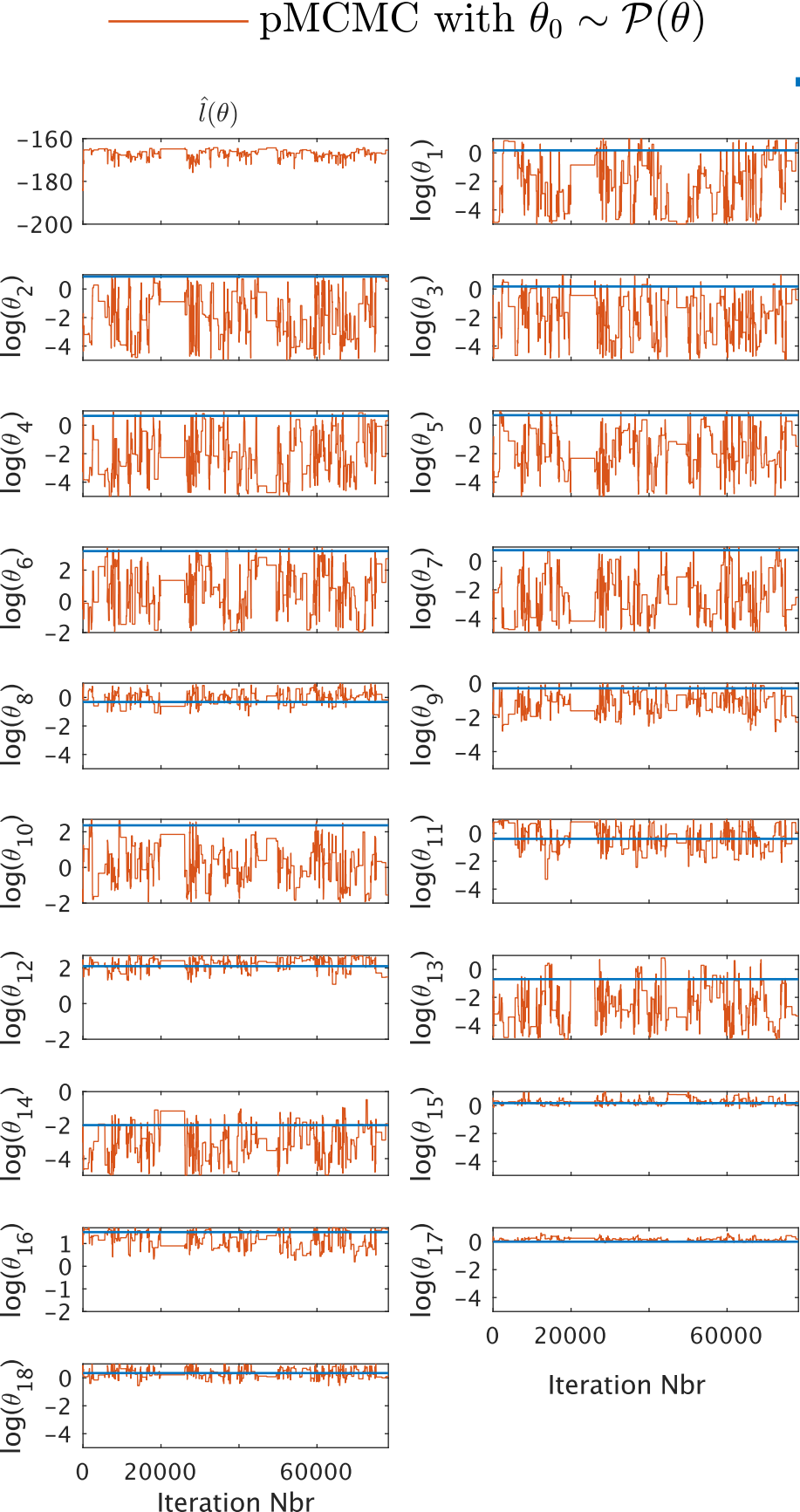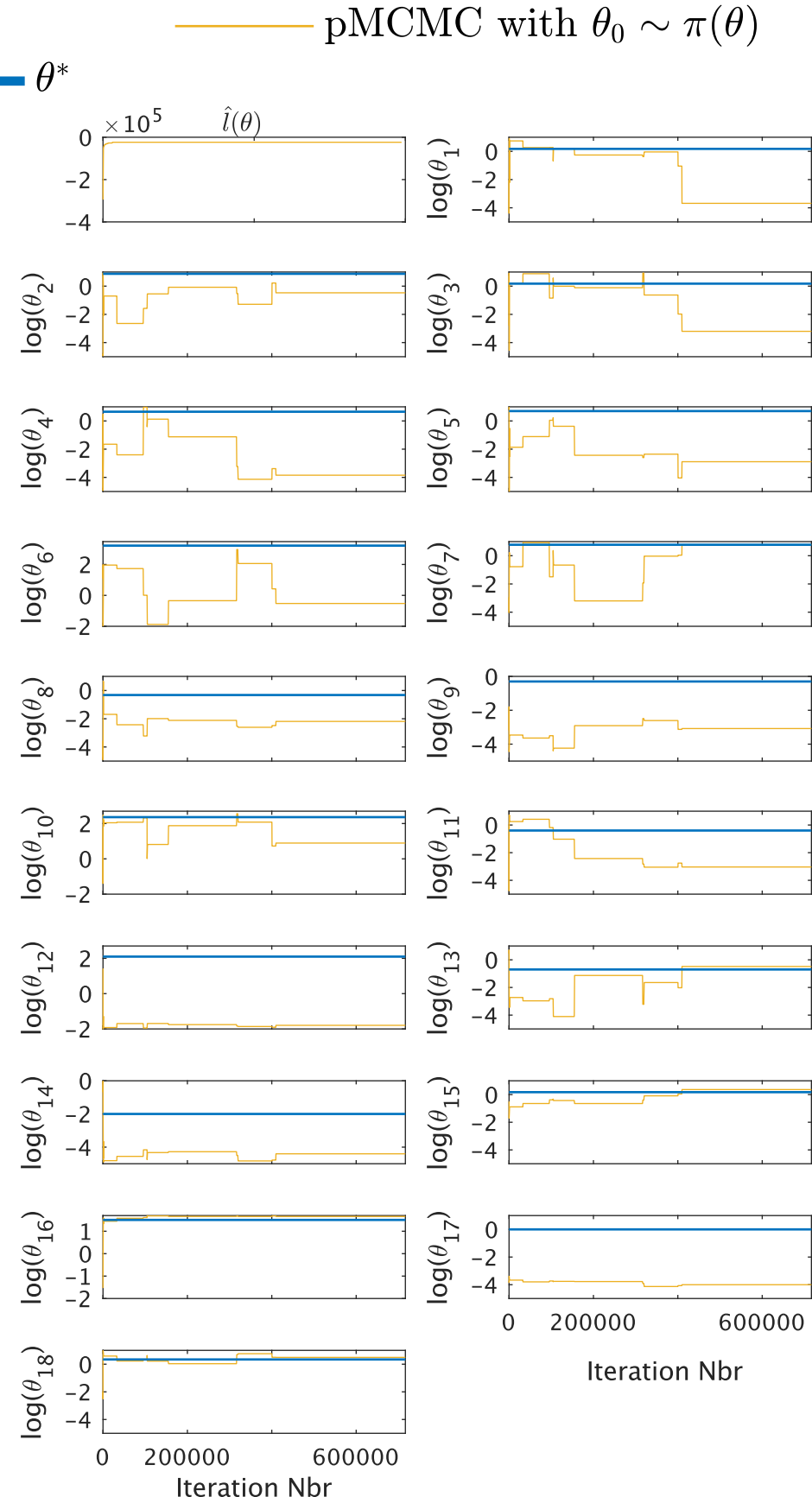

Supplement: S9 Fig — pMCMC development of the individual parameters fro the two pMCMC runs with different initial samples θ0 Left: pMCMC run with θ0∼P(θ) Right: pMCMC run with θ0 ∼ π(θ). (PDF) [file pcbi.1008264.s017.pdf]

$\theta^*$     LF-NS posterior     $F = 0$      $F = 38$      $F = 76$      $F = 114$      $F = 152$      $F = 190$

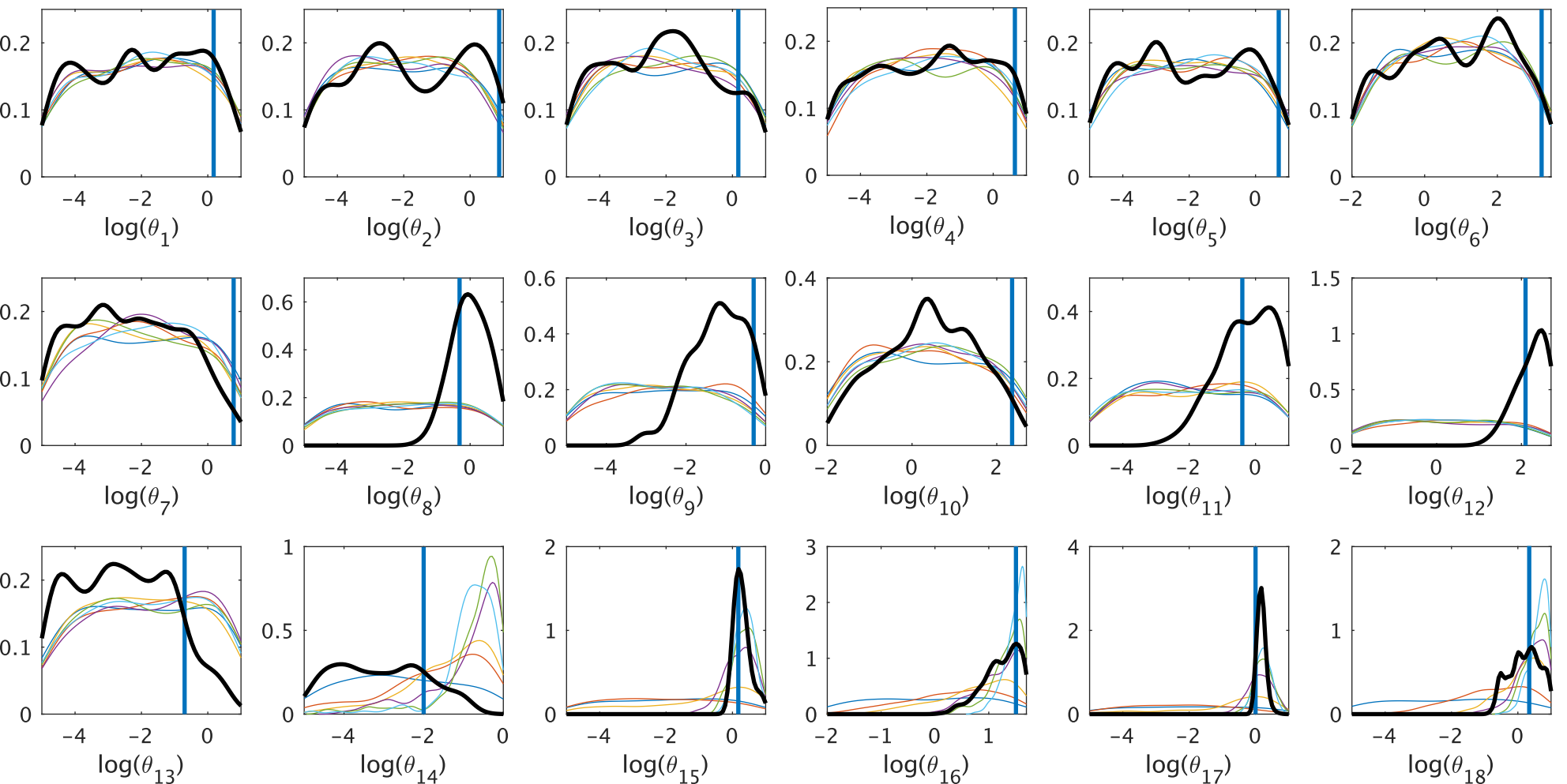

Supplement: S10 Fig — The true parameter θ* is indicated as the blue line. The marginal posterior obtained from the LF-NS run (as described in the main paper) is plotted as thick black line. The distributions p(θ|d(yθ|y) < ϵF) are shown in thin lines. (PDF) [file pcbi.1008264.s018.pdf]

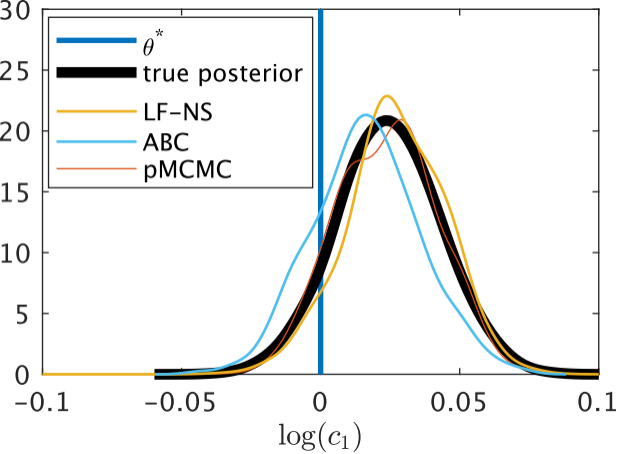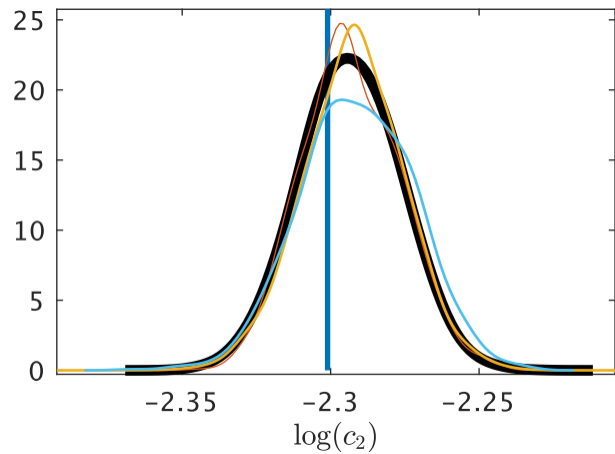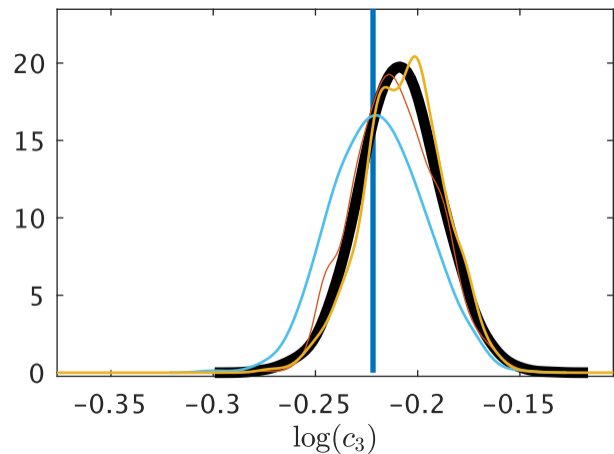

Supplement: S11 Fig — The obtained posteriors for all three algorithms, LF-NS, pMCMC and ABC-SMC are indicated. The posteriors were obtained using 12 minutes of computation for all three algorithms. The thick black line indicates the posterior as obtained with a long run of pMCMC. (PDF) [file pcbi.1008264.s019.pdf]

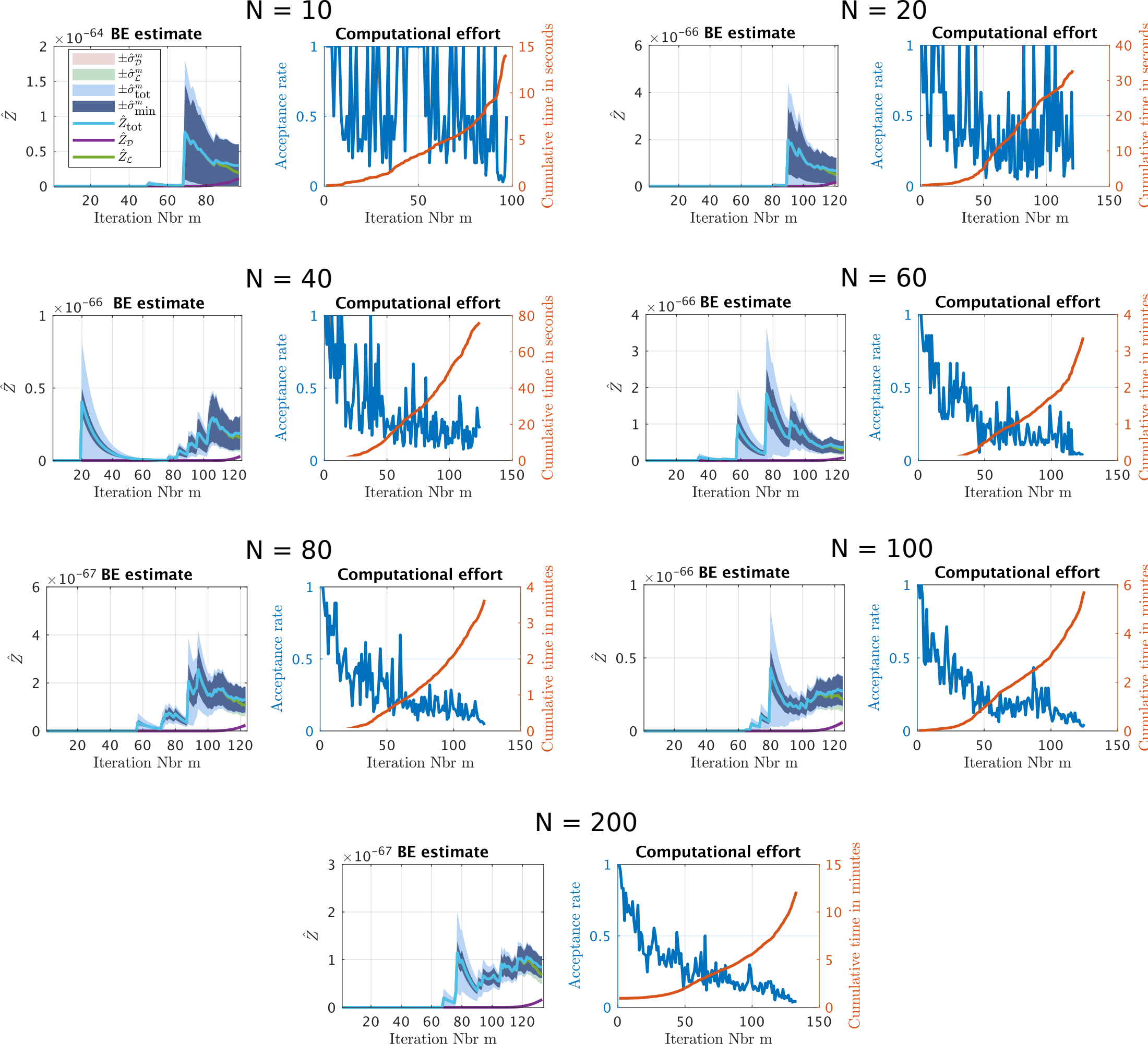

Supplement: S12 Fig — Left: Evidence using the estimation based solely on the dead points Z^D, the estimate approximation from the live points Z^L and the estimation based on both Z^tot. The corresponding standard errors are indicated as the shaded areas. Right: Acceptance rate and cumulative runtime for each iteration. (PDF) [file pcbi.1008264.s020.pdf]
