# Supplementary material for: Likelihood-free nested sampling for parameter inference of biochemical reaction networks
Source: PLoS Comput Biol. 2020 Oct 9;16(10):e1008264. doi: 10.1371/journal.pcbi.1008264 (PMC7577508; doi:10.1371/journal.pcbi.1008264)
Supplement: S1 Table — (PDF) [file pcbi.1008264.s021.pdf]

Table S1: Species and intial numbers of the Lac-Gfp model

Species	Notation	Initial Distribution
LacI mRNA	$lacI$	U([0, 5])
LacI protein monomer	$LACI$	U([0, 10])
LacI dimer	$LACI2$	fixed to 0
Unoccupied (active) Lac promoter	$PLac$	fixed to 0
Occupied Lac promoter with 2 repressor molecules bound	$O2Lac$	fixed to 0
Occupied Lac promoter with 4 repressor molecules bound	$O4Lac$	U([50, 70])
GFP mRNA	gfp	fixed to 0
"Dark" GFP protein	GFP	fixed to 0
Mature GFP protein	$mGFP$	fixed to 0

U($[a, b]$) denotes the uniform distribution on the interval $[a, b]$.
