# Supplementary material for: Likelihood-free nested sampling for parameter inference of biochemical reaction networks
Source: PLoS Comput Biol. 2020 Oct 9;16(10):e1008264. doi: 10.1371/journal.pcbi.1008264 (PMC7577508; doi:10.1371/journal.pcbi.1008264)
Supplement: S2 Table — (PDF) [file pcbi.1008264.s022.pdf]

Table S2: Prior distributions of the Lac-GFP parameters and the real values $\boldsymbol{\theta}^*$ used for the simulation of $\boldsymbol{y}$.

Parameter	Meaning	Prior interval	$\boldsymbol{\theta}^*$
θ_1	lacI transcription rate	$[10^{-5}, 10]$	1.5
θ_2	lacI degradation rate	$[10^{-5}, 10]$	7.5
θ_3	LACI translation rate	$[10^{-5}, 10]$	1.5
θ_4	IPTG-independent LACI degradation rate	$[10^{-5}, 10]$	4.5
θ_5	IPTG-induced increase in LACI degradation rate	$[10^{-5}, 10]$	5
θ_6	Dimerization rate of LACI	[0.1, 3000]	1650
θ_7	Dissociation rate of LACI dimers	$[10^{-5}, 10]$	6
θ_8	Binding rate of LACI dimers to Lac promoter	$[10^{-5}, 10]$	0.48
θ_9	Dissociation rate of LACI dimers from Lac promoter	$[10^{-5}, 1]$	0.5
θ_{10}	Tetramerization rate of LACI	[0.01, 500]	230
θ_{11}	Dissociation rate of LACI tetramers	$[10^{-5}, 10]$	0.4
θ_{12}	gfp transcription rate from free PLac promoter	[0.01, 500]	125
θ_{13}	gfp transcription rate from LACI dimer-bound PLac	$[10^{-5}, 10]$	0.2
θ_{14}	gfp transcription rate from LACI tetramer-bound PLac	$[10^{-5}, 1]$	0.01
θ_{15}	gfp degradation rate	$[10^{-5}, 10]$	1.5
θ_{16}	GFP translation rate	[0.01, 50]	32
θ_{17}	GFP degradation rate	$[10^{-5}, 10]$	1
θ_{18}	GFP maturation rate	$[10^{-5}, 10]$	2.2

For all the Lac-Gfp inference problems presented in this paper, each parameter was assigned an independent uniform log prior distribution in the interval listed in the table.
