# Supplementary material for: Likelihood-free nested sampling for parameter inference of biochemical reaction networks
Source: PLoS Comput Biol. 2020 Oct 9;16(10):e1008264. doi: 10.1371/journal.pcbi.1008264 (PMC7577508; doi:10.1371/journal.pcbi.1008264)
Supplement: S3 Table — (PDF) [file pcbi.1008264.s023.pdf]

Table S3: Prior distributions of the parameters for the transcription model.

Parameter	Meaning	Prior interval
k_{on}	promoter binding rate	$[10^{-5}, 100]$
k_{off}	promoter unbinding rate	$[10^{-5}, 100]$
k_{r}	mRNA transcpription	$[0.1, 500]$
λ	transcription rate	$[2, 8]$

For the transcription model inference problem presented in this paper, each parameter was assigned an independent uniform log prior distribution in the interval listed in the table.
