# Supplementary material for: Likelihood-free nested sampling for parameter inference of biochemical reaction networks
Source: PLoS Comput Biol. 2020 Oct 9;16(10):e1008264. doi: 10.1371/journal.pcbi.1008264 (PMC7577508; doi:10.1371/journal.pcbi.1008264)
Supplement: S4 Table — (PDF) [file pcbi.1008264.s024.pdf]

Table S4: Species and intial numbers of the Lotka-Voltera model

Species	Notation	Initial Distribution	Value used for data simulation
Prey	X_1	Poisson(50)	50
Predator	X_2	Poisson(100)	100
