# Supplementary material for: Likelihood-free nested sampling for parameter inference of biochemical reaction networks
Source: PLoS Comput Biol. 2020 Oct 9;16(10):e1008264. doi: 10.1371/journal.pcbi.1008264 (PMC7577508; doi:10.1371/journal.pcbi.1008264)
Supplement: S5 Table — (PDF) [file pcbi.1008264.s025.pdf]

Table S5: Prior distributions of the parameters for the Lotka-Voltera model.

Parameter	Meaning	Prior interval	θ^*
c_1	Prey birth	$[\exp(-6.2)\,\exp(2)]$	1
c_2	Predator consumptin of Prey	$[\exp(-6.2)\,\exp(2)]$	0.005
c_3	Predator death	$[\exp(-6.2)\,\exp(2)]$	0.6

For the Lotka-Voltera model inference problem presented in this paper, each parameter was assigned an independent uniform log prior distribution in the interval listed in the table.
